# miR‐322 treatment rescues cell apoptosis and neural tube defect formation through silencing NADPH oxidase 4

**DOI:** 10.1111/cns.13383

**Published:** 2020-04-24

**Authors:** Yu‐si Liu, Hui Gu, Tian‐chu Huang, Xiao‐wei Wei, Wei Ma, Dan Liu, Yi‐wen He, Wen‐ting Luo, Jie‐ting Huang, Duan Zhao, Shan‐shan Jia, Fang Wang, Ting Zhang, Yu‐zuo Bai, Wei‐lin Wang, Zheng‐wei Yuan

**Affiliations:** ^1^ Key Laboratory of Health Ministry for Congenital Malformation Shengjing Hospital China Medical University Shenyang China; ^2^ Department of Pediatric Surgery Shengjing Hospital China Medical University Shenyang China; ^3^ Beijing Municipal Key Laboratory of Child Development and Nutriomics Capital Institute of Pediatrics Beijing China

**Keywords:** all‐trans‐retinoic acid, apoptosis, miR‐322, NADPH oxidase 4, neural tube defects

## Abstract

**Aims:**

Failure of neural tube closure resulting from excessive apoptosis leads to neural tube defects (NTDs). NADPH oxidase 4 (NOX4) is a critical mediator of cell growth and death, yet its role in NTDs has never been characterized. NOX4 is a potential target of miR‐322, and we have previously demonstrated that miR‐322 was involved in high glucose‐induced NTDs. In this study, we investigated the effect of NOX4 on the embryonic neuroepithelium in NTDs and reveal a new regulatory mechanism for miR‐322 that disrupts neurulation by ameliorating cell apoptosis.

**Methods:**

All‐trans‐retinoic acid (ATRA)‐induced mouse model was utilized to study NTDs. RNA pull‐down and dual‐luciferase reporter assays were used to confirm the interaction between NOX4 and miR‐322. In mouse neural stem cells and whole‐embryo culture, Western blot and TUNEL were carried out to investigate the effects of miR‐322 and NOX4 on neuroepithelium apoptosis in NTD formation.

**Results:**

NOX4, as a novel target of miR‐322, was upregulated in ATRA‐induced mouse model of NTDs. In mouse neural stem cells, the expression of NOX4 was inhibited by miR‐322; still further, NOX4‐triggered apoptosis was also suppressed by miR‐322. Moreover, in whole‐embryo culture, injection of the miR‐322 mimic into the amniotic cavity attenuated cell apoptosis in NTD formation by silencing NOX4.

**Conclusion:**

miR‐322/NOX4 plays a crucial role in apoptosis‐induced NTD formation, which may provide a new understanding of the mechanism of embryonic NTDs and a basis for potential therapeutic target against NTDs.

## 1 INTRODUCTION

Neural tube defects (NTDs) are severe congenital malformations caused by the failure of neural tube closure, a morphogenetic process originating during embryogenesis. In NTDs, failure of closure in the spinal cord and brain leads to spina bifida and anencephaly.^1^ NTDs are a significant cause of fetal mortality and morbidity; NTD prevalence is about 6.0 per 10,000 births, worldwide.^2^ Apoptosis appears to be indispensable during the development of the neural tube; however, excessive apoptosis may lead to an insufficient number of cells within the neural folds, or destroy the continuity of the dorsal midline, ultimately causes NTDs.^3^ Likewise, increased cellular apoptosis contributes to the formation of all‐trans retinoic acid (ATRA)‐induced NTDs.^4^, ^5^ Therefore, we used ATRA to construct NTD animal models, allowing us to identify potential risk factors and to investigate novel preventive therapies.

MicroRNAs (miRNAs) are naturally occurring, noncoding small RNA molecules that degrade the target mRNA or target the 3’‐untranslated region (UTR) of mRNA molecules to repress their translation.^6^, ^7^ miRNAs are highly expressed in the nervous system and have been studied recently.^8^, ^9^ For instance, our group has reported that miRNAs in maternal serum such as miR‐765, miR‐144, and miR‐720 can be useful as biomarkers for noninvasive prenatal diagnosis of NTDs.^10^ In addition, we found that the miR‐200b‐CITED2 circuit mediated the effect of high glucose on unfolded protein response activation and endoplasmic reticulum stress leading to apoptosis in neural stem cells.^11^ Wu et al^12^ reported the alteration of miRNAs in NTDs and concluded that overexpression of miR‐451 and miR‐375 along with the consequent upregulation of p53 may further promote apoptosis in NTDs.

miR‐322, located on the X chromosome, is differentially expressed in various diseases and is highly conserved in various cell types.^13^ It has previously been investigated as a vital regulator of the cell cycle, proliferation, apoptosis and differentiation. For instance, miR‐322 promotes CDK2 inhibition and induces a G1 phase cell cycle arrest in myoblasts during their differentiation into myotubes.^14^ Kim et al^15^ reported that miR‐322 had an antiproliferative effect in pulmonary hypertension, which was mediated by the regulation of its targets FGF2 and FGFR1. miR‐322 can also promote osteoblast differentiation by downregulating TOB2‐ and TOB2‐regulated osteogenic genes.^16^ Furthermore, our previous studies have found that miR‐322 was underexpressed in NTDs caused by diabetes and inhibited apoptosis through TRAF3 in high glucose‐treated neural stem cells.^17^ However, the precise role of miR‐322 on NTDs and the underlying mechanism remain far from understood.

The NADPH oxidase (NOX) family are well‐known generators of superoxide anion; NOX4 is the most widely expressed NOX isoform among the members of the NOX family.^18^, ^19^ The majority of recent studies have shown that genetic and pharmacological inhibition of NOX4 is neuroprotective. Furthermore, NOX4 plays a key role in demyelination after peripheral nerve injury and inducible deletion of NOX4 can attenuate neuropathic pain behavior.^20^ A decrease in NOX4 expression or activity can mitigate cognitive impairment in a transgenic mouse model of Parkinson's disease.^21^ Taken together, these data show that NOX4 plays a critical role in the nervous system; however, no data exist on the function of the NOX4 in NTDs.

Therefore, the aim of the present study is to define the function of miR‐322 and NOX4 in NTDs. According to our bioinformatic analysis, NOX4 is a novel target for miR‐322. RNA pull‐down and dual‐luciferase assays were performed to demonstrate the interaction between miR‐322 and NOX4. Furthermore, in neural stem cells and ex vivo embryos, we investigated the relationship between miR‐322 and NOX4, as well as their effect on apoptosis. Especially, we injected the miR‐322 mimic into the amniotic cavity of ex vivo embryos to confirm the effect of miR‐322 on apoptosis in ATRA‐induced mouse model of NTDs. These findings may provide a new understanding of the mechanism of NTDs and a basis for potential therapeutic target against NTDs.

## 2 METHODS

### 2.1 Animal models

We obtained adult female C57B1/6J mice, aged 8‐10 weeks, from the Animal Center of Shengjing Hospital associated with China Medical University (Shenyang, Liaoning, China). On embryonic day 8.5 (E8.5), pregnant mice were treated with ATRA (70 mg/kg body weight; Sigma‐Aldrich) or control treatment (olive oil only).^22^, ^23^ Mice were euthanized via cervical dislocation 24 hour after treatment, on E9.5. Using a dissecting microscope (Olympus Szhillb), embryos were removed, and images obtained. The animal ethics committee of Shengjing Hospital of China Medical University (Approval No. 2016PS113K) approved all experimental protocols; every effort was made to minimize pain and suffering of the mice.

### 2.2 Cell culture and transfection

Mouse neural stem cells (C17.2) were obtained from Beina Chuanglian Biology Research Institute. Cells were cultured in Minimum Essential Medium (MEM, Gibco) supplemented with 10% fetal bovine serum (Gibco), 1% MEM nonessential amino acids (MEM NEAA, Gibco), and 1% penicillin/streptomycin (100 U/mL and 100 μg/mL, respectively; Life Technology Inc). Cells were cultured under 5% carbon dioxide at 37°C in a humidified incubator. Cells were seeded into six‐well plate at 1 × 10^5^ cells per well and cultured for 24 hour before transfection. JetPRIME (Polyplus Transfection) was used as transfection reagent. Transfections were carried out according to manufacturers’ protocols.

### 2.3 Reagents and constructs

Oligonucleotides including the miRNA mimic, the miRNA inhibitor of miR‐322, and appropriate negative control oligos were purchased from GenePharma. Biotin‐labeled miRNA‐322 was custom‐designed and purchased from Dharmacon. We amplified and subcloned the 3’‐UTR of NOX4 and a portion of the predicted miR‐322 binding site (BS) in its 3’‐UTR and inserted these sequences into the pmirGLO Dual‐Luciferase miRNA Target Expression Vector (Promega), thus generating the 3’UTR‐Luc and BS‐Luc constructs (Liaoning Bai Hao biological Technology Co. Ltd.). Then, NOX4 3’‐UTR binding site was subsequently removed by generating an internal mutation to generate the Mut‐Luc construct (Liaoning Bai Hao biological Technology Co. Ltd). The primer of miR‐322, U6, NOX4, and β‐actin was made by Sangong Biotech. Short hairpin RNAs (shRNAs) for NOX4, plasmids for overexpression of NOX4, and negative controls were purchased from GeneChem. The Table S1 provides all primer sequences used to generate these constructs.

### 2.4 Biotin‐labeled miR‐322 pull‐down assay

Mouse neural stem cells (C17.2) were transfected with biotin‐labeled miR‐322 or biotin‐labeled negative control (*C elegans* miR‐67); cell lysate was collected 48 hour after transfection. Dynabeads coupled to streptavidin (Invitrogen were added to the lysates and incubated overnight at 4°C. Beads were washed, and then, bound RNA was eluted and used for quantitative real‐time PCR (qRT‐PCR) analyses.

### 2.5 Dual‐luciferase reporter assay

Forty‐eight hours after transfection with indicated NOX4 luciferase reporter, together with miR‐322 mimic or negative control, the dual‐luciferase reporter assay system (Promega) was utilized according to the instructions provided by the manufacturer. The ratio of firefly luciferase activity to *Renilla* luciferase activity was calculated and used as relative luciferase activity.

### 2.6 Whole‐embryo culture

After euthanasia at E9.5, mouse embryos were separated and the parietal yolk sac was removed, leaving the visceral yolk sac was left intact. Next, miR‐322‐5p agomir (1 nmol per embryo, RiboBio) or its negative control was injected into the amniotic cavity, and then, the embryo was transferred to rat serum with ATRA (0.01 M/L) or control (dimethyl sulfoxide, DMSO). Using a roller bottle system, embryos (4/bottle) were cultured in 4 mL of rat serum containing 2 mg/mL glucose at 37°C at a 30‐rpm rotation. Culture bottles were cultured under 20% O_2_/5% CO_2_/90% N_2_ for 24 hour; then, O_2_ was increased to 40% for the next 24 hour. Finally, the embryos were checked for survival and were harvested and photographed.

### 2.7 Histology and immunohistochemistry

After experimentation and harvest as described above, mouse embryos were fixed in 4% paraformaldehyde, embedded in paraffin, and then cut into 4‐μm‐thick sections using a microtome. Sections were mounted on slides, deparaffinized in xylene, and hydrated with a graded series of ethanol.

To analyze spinal cord morphology, conventional hematoxylin‐eosin (HE) staining was performed. Each section was stained for three minutes with hematoxylin solution and then rinsed with distilled water. The sections were next stained with eosin solution for one minute, dehydrated with graded alcohol, and cleared with xylene. Images were acquired using a fluorescence microscope (Nikon ECLIPSE 80i, Japan).

An UltraSensitive SP IHC Kit (MaiXin‐Bio) was used to perform immunohistochemistry (IHC) analysis. A rabbit polyclonal anti‐NOX4 antibody (1:800; ROCKLAMD) was used for analyzing of NOX4 protein in NTDs tissues. A DAB plus from Maixin (Maixin‐Bio) was used to visualize the detected markers, and results were imaged using a fluorescence microscope (Nikon ECLIPSE 80i). NOX4 expression was semi‐quantitatively scored based on the percentage of expression and signal intensity. The intensity staining score was indicated as 0 (no staining), 1 (weak staining), or 2 (strong staining). Staining percentage was scored as 0 (0%), 1 (1%‐25%), 2 (26%‐50%), 3 (51%‐75%), and 4 (76%‐100%). Final scores were calculated as the multiplication of intensity staining times staining percentage. All sections were randomly analyzed by two independent investigators.

### 2.8 RNA extraction and qRT‐PCR

An miRNeasy Mini Kit (Qiagen) was used to extract total RNA, containing miRNA, from mouse embryos or mouse neural stem cells (C17.2). Extracted RNA was reverse‐transcribed using a miRNA First Strand cDNA Synthesis (Sangon). qRT‐PCR was performed as follows: pre‐denaturation at 95°C for 5 minutes; followed by 45 cycles of 95°C for 15 s and 60°C for 45 s using a SYBR Premix Ex Taq kit (Takara) on a 7500 Real‐time PCR system (ABI). The relative expression of miRNAs and cDNA was quantified using the 2^−ΔΔCt^ method.

### 2.9 Western blotting

Protein was extracted from mouse embryos or mouse neural stem cells (C17.2). Approximately 40µg of protein from each sample was separated by sodium dodecyl sulfate‐polyacrylamide gel electrophoresis (SDS‐PAGE) followed by electro‐transferred to polyvinylidene difluoride (PVDF) membranes. A nonfat milk solution (5%) was used for blocking before incubation with primary antibodies against NOX4 (1:1000, ROCKLAND), Bax (1:1000, Cell Signaling Technology), cleaved caspase‐3 (1:500, Cell Signaling Technology), or Bcl‐2 (1:1000, Sigma‐Aldrich) overnight at 4°C. Membranes were washed three times for 10 minutes with TBST (tris‐buffered saline plus Tween‐20) and then incubated with secondary antibody (1:5000, Invitrogen) at room temperature for 2 hour. Enhanced chemiluminescent (ECL) reagent (Millipore) was used to detect protein‐antibody interactions.

### 2.10 Immunofluorescence staining

Sections were deparaffinized in xylene, hydrated with a graded series of ethanol, and treated with 0.1% Triton X‐100 to permeabilize the cell membrane. The tissue were then blocked with nonimmunized animal serum and incubated with antibodies against NOX4 (1:1000, ROCKLAND) at 4°C overnight. The sections were rinsed with PBS three times and incubated with a fluorescently labeled secondary antibody (1:200; Cell Signaling Technology) for 2 hours at room temperature. DAPI was applied to stain the nuclei, and results were imaged using a fluorescence microscope.

### 2.11 TUNEL assay

An in situ cell death assay kit, TMR red (Rocho), was used to conduct TUNEL stain. Cells were seeded onto 8‐well glass slide (Thermo Fisher Scientific) and transfected. Tissues were cut into 4‐μm sections. Then, cells or tissue were incubated with TUNEL reagent. DAPI staining was used for enumeration and identification of nuclei. A fluorescence microscope (Nikon ECLIPSE 80i) was used to images samples. The number of TUNEL‐positive cells was counted.

### 2.12 Statistics

Statistical analyses and graph generation were performed using GraphPad Prism 7.0 software (GraphPad Software). All results were expressed as the mean ± standard error of the mean (SEM) without special instructions. For two groups, if data were in normal distribution, Student's *t* test (two‐tailed) was used. Otherwise, the Mann‐Whitney *U* test was used. One‐way ANOVA, followed by a Dunnett post hoc test for multiple comparisons, was used to analyze the data from multiple groups. The ratio of NTDs in whole‐embryo culture was analyzed by X^2^ test. *P* < .05 was considered statistically significant.

## 3 RESULTS

### 3.1 miR‐322 and NOX4 expression are changed in ATRA‐induced mouse model of NTDs

**Figure 1:**
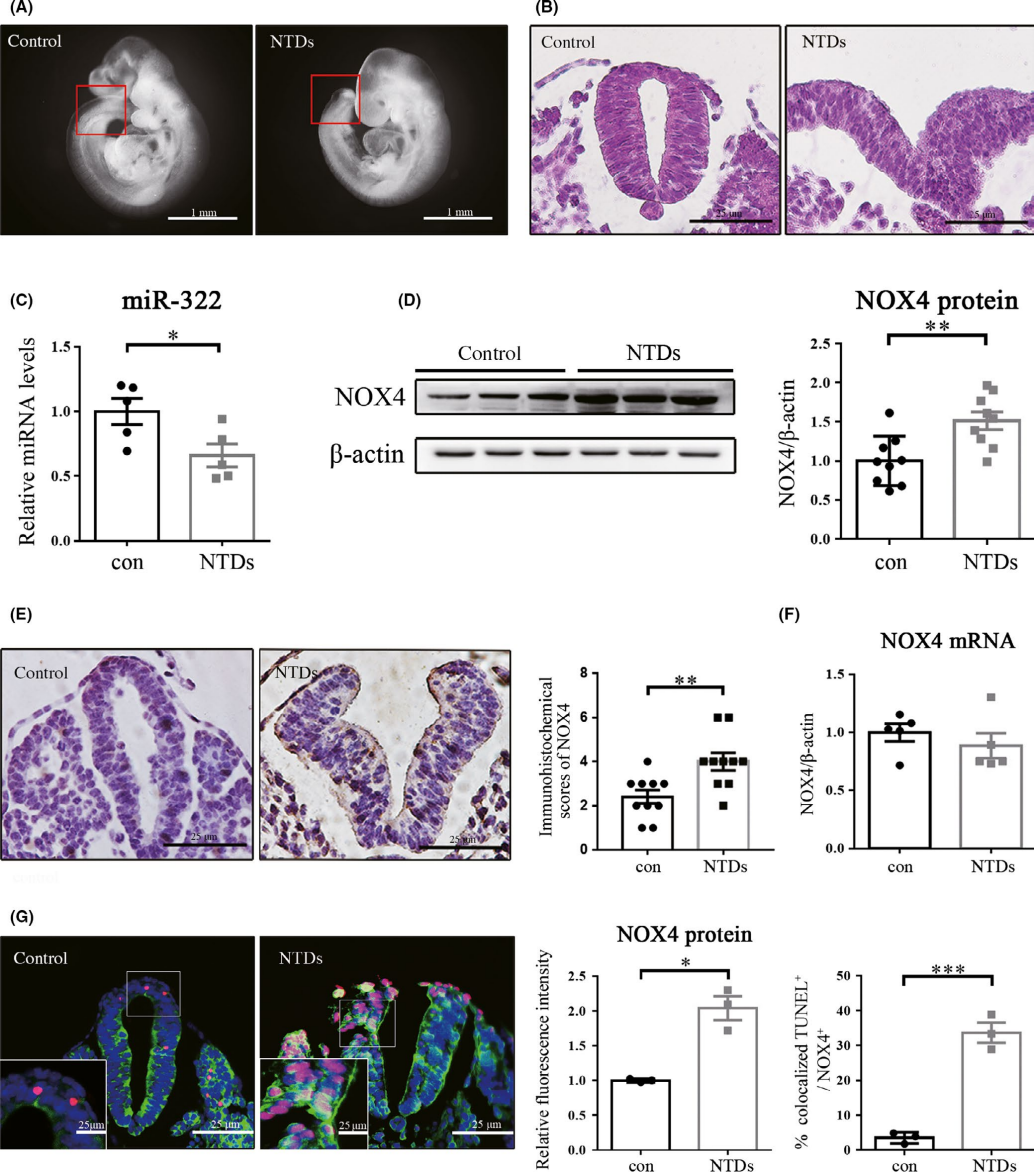
Expression of miR‐322 and NOX4 in NTDs caused by ATRA. (A) Morphology of normal and NTDs embryos at E9.5. (B) HE staining images of the neural tube transverse sections of normal and NTD embryos at E9.5. (C) Relative expression of miR‐322 was measured by qRT‐PCR using U6 as an internal control. (D) Western blot was performed to examine NOX4 levels using β‐actin as an internal control. (E) Representative IHC images of NOX4 expression in neural tube at E9.5 and semi‐quantitative analysis of the IHC results. (F) The relative expression of NOX4 mRNA was tested by qRT‐PCR using β‐actin as an internal control. (G) Representative immunofluorescence results of NOX4 (green) and the TUNEL signal (red) in E9.5 samples. DAPI (blue) was counterstained to reveal the nucleus. Statistical analysis of the fluorescence intensity and the percentage of TUNEL positive signal colocalized with strong NOX4 expression. The result represents three repeated experiments. “*,” “**,” and “***” represented *P* < .05, *P* < .01, and *P* < .001, respectively

At E9.5, the tissue morphology of the embryo was observed. As shown in Figure 1A, the embryos in the control group developed normally, with plump and round tails and closed neural tubes. Malformed embryos showed delayed development and failed closure of the neural tube. HE staining revealed the tissue morphology of neural tube parts in normal and NTD mice (Figure 1B). Next, the expression of miR‐322 and NOX4 was investigated in NTD models. Levels of miR‐322 were lower in NTD group as compared with controls (Figure 1C). NOX4 protein expression was higher (Figure 1D), yet there were no changes in NOX4 mRNA levels (Figure 1F). IHC also revealed that total NOX4 levels was increased in NTD samples compared with control tissues (Figure 1E). To determine the possible relationship between NOX4 and apoptosis, immunofluorescence of NOX4 was colocalized with the TUNEL signal in E9.5 tissues. As shown in Figure 1G, NOX4 was observed to be generally expressed in neural tubes, but the expression of NOX4 in NTD group was more evident. In control group, only a few TUNEL‐positive cells were found. In contrast, a large number of TUNEL‐positive cells were found in NTDs, and the apoptotic TUNEL signal was frequently colocalized with the dots of strong NOX4 expression.

### 3.2 miR‐322 interacts with its putative target gene NOX4

**Figure 2:**
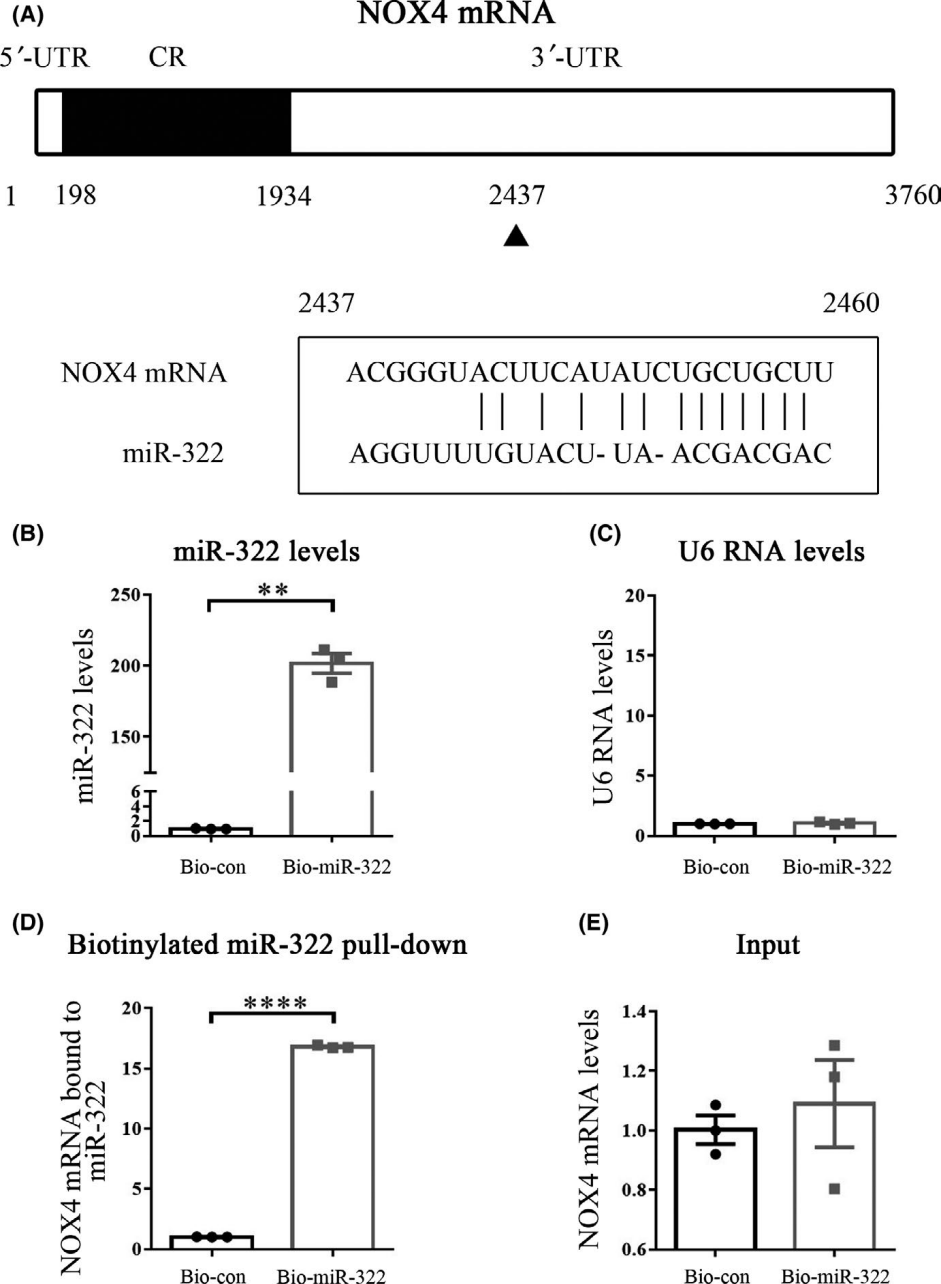
miR‐322 interacts with its target gene, NOX4. (A) Schematic representation of the NOX4 mRNA depicting miR‐322 binding sites in its 3’‐UTR. One predicted miR‐322 binding site (position 2437‐2460) is located in the 3’‐UTR of NOX4 mRNA. The effect of miR‐322 (B) and U6 (C) on the mRNA expression level at 48 hour after biotin‐miR‐322 transfection was detected using qRT‐PCR. (D) NOX4 mRNA level in the precipitates pulled down by biotin‐miR‐322 and biotin‐labeled negative control was detected using qRT‐PCR. (E) qRT‐PCR was used to detect the expression level of input NOX4 mRNA. All experiments were performed in triplicate. “**” and “****,” respectively, represented *P* < .01 and *P* < .0001, compared with the Bio‐con group (transfected with biotin‐labeled scrambled control miRNA)

NOX4 that appeared to be the most likely target of miR‐322 among three databases (ie, microRNA.org, TargetScan, starBase) was identified as a candidate gene. One potential binding site for miR‐322 is located in the 3’‐UTR of NOX4 mRNA (2437‐2460) (Figure 2A). In order to verify whether NOX4 is a true target of miR‐322, we used a pull‐down assay. The expression levels of miR‐322 and small nuclear RNA U6 were measured after 48‐hour transfection. We found that cells transfected with biotin‐labeled miR‐322 had higher levels of miR‐322 (Figure 2B) but no changes in RNA U6 expression (Figure 2C). The levels of NOX4 mRNA displayed a marked increase in the cells transfected with the biotin‐labeled miR‐322 compared with those transfected with biotin‐labeled scrambled control miRNA (Figure 2D). However, the level of NOX4 mRNA was similar between input samples from the two groups (Figure 2E).

### 3.3 miR‐322 suppresses NOX4 translation

**Figure 3:**
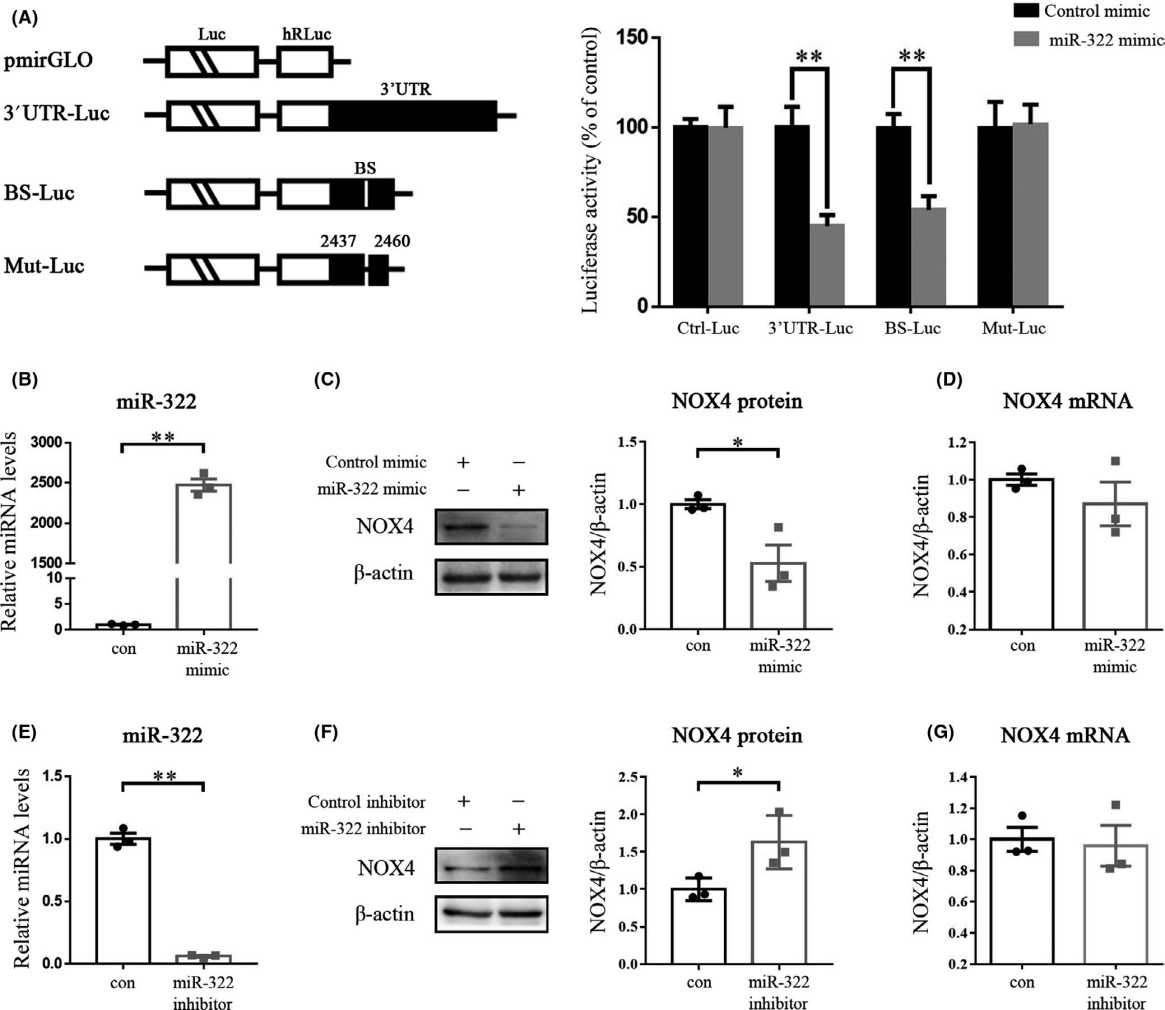
miR‐322 suppresses NOX4 translation. (A) Levels of reporter activity as measured by analysis of the 3’UTR‐Luc, BS‐Luc, and Mut‐Luc luciferase reporters after overexpression of miR‐322. Left, plasmid schematic of different chimeric firefly luciferase‐NOX4 reporters. Luc, luciferase; BS, miR‐322‐binding site; Mut, mutation. Right, the luciferase activities levels of 3’UTR‐Luc, BS‐Luc, and Mut‐Luc reporters. C17.2 cells were transfected with different NOX4‐luciferase reporter plasmids, together with miR‐322 mimic or negative control. Levels of firefly and *Renilla* luciferase activities were assayed 48 hour later. Results were normalized to the *Renilla* luciferase activity. (B) miR‐322 levels in cells transfected with the miR‐322 mimic. Protein abundance (C) and mRNA levels (D) of NOX4 after miR‐322 mimic transfection. (E) miR‐322 levels in cells transfected with the miR‐322 inhibitor. Effect of the miR‐322 inhibitor on NOX4 protein (F) and mRNA (G) expression. The assay was repeated for three times. “*” and “**” represented *P* < .05 and *P* < .01, respectively

We performed a luciferase reporter assay to further verify the ability of miR‐322 on NOX4 expression. We subcloned the 3’UTR of NOX4 mRNA (contains a miR‐322 binding site) or just its predicted miR‐322 BS into the pmirGLO dual‐luciferase miRNA target expression vector, thus generating the 3’UTR‐Luc reporter and the BS‐Luc reporter (Figure 3A). Additionally, we generated a Mut‐Luc reporter by internally mutating nucleotides at positions 2437‐2460, which comprise the putative miR‐322 binding site (Figure 3A). The luciferase activity of the 3’UTR‐Luc and the BS‐Luc reporter was decreased evidently when miR‐322 was overexpressed. However, the luciferase activity of Mut‐Luc was unchanged when miR‐322 overexpressed, likely due to the lack of the putative miR‐322 binding site (Figure 3A). Altogether, these results indicate that the reduction in luciferase activity was caused by miR‐322 and that there was an interaction between miR‐322 and NOX4 through the binding site in the NOX4 3’‐UTR.

Next, we used the miR‐322 mimic or miR‐322 inhibitor to transfect C17.2 mouse neural stem cells to study the effect of miR‐322 on NOX4. The levels of miR‐322 were upregulated, and the protein expression of NOX4 was correspondingly downregulated when the miR‐322 mimic was used (Figure 3B,C). However, the NOX4 mRNA levels did not change significantly (Figure 3D). And vice versa, miR‐322 inhibitor repressed miR‐322 levels (Figure 3E) and increased NOX4 protein levels (Figure 3F), but not mRNA levels (Figure 3G).

### 3.4 miR‐322 reverses cell apoptosis induced by NOX4

**Figure 4:**
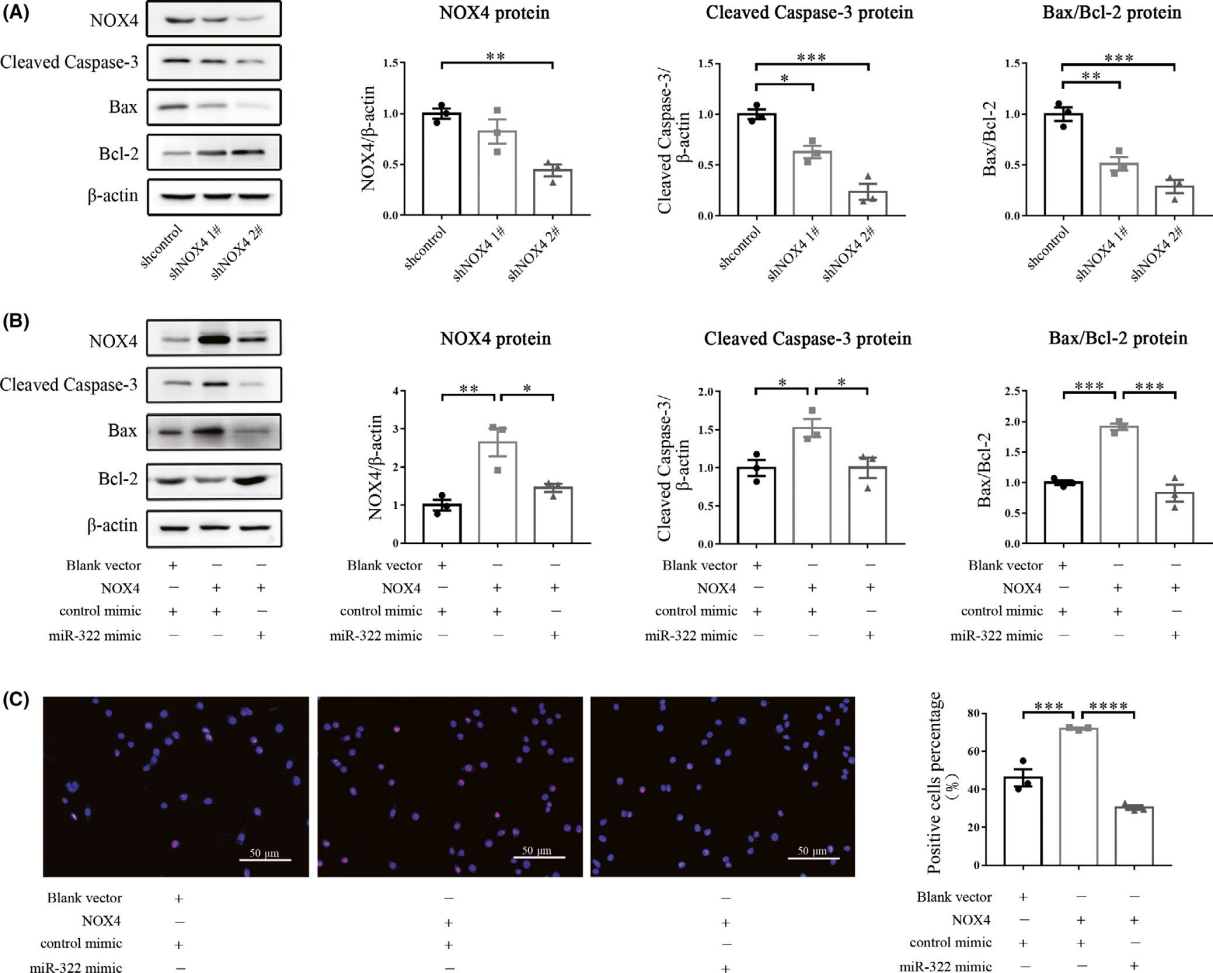
miR‐322 attenuates NOX4‐triggered cell apoptosis. (A) The protein expression of cleaved caspase‐3, Bax, Bcl‐2, and NOX4 was determined by Western blot using β‐actin as an internal control. (B) Cleaved caspase‐3, Bax, Bcl‐2, and NOX4 protein levels were determined by Western blot after cells were transfected with NOX4 and/or the miR‐322 mimic. (C) Representative images of the TUNEL assay. Cells were transfected with the NOX4 full‐length expression plasmid, and cotransected with miR‐322 mimic or control mimic. Apoptotic cells were labeled by the TUNEL reagents and all cell nuclei were stained by DAPI. These assays were repeated three times. *P < .05, **P < .01, ***P < .001, ****P < .0001

To investigate the effects of miR‐322/NOX4 on apoptosis, we examined apoptosis‐related proteins and performed a TUNEL assay. We constructed both the short hairpin plasmid and the full‐length plasmid containing NOX4 coding sequences. shNOX4 2#, which reduced NOX4 protein expression more effectively than shNOX4 1#, inhibited the expression of pro‐apoptotic proteins cleaved caspase‐3 and Bax, while upregulating the anti‐apoptotic protein Bcl‐2 (Figure 4A). Conversely, overexpression of NOX4 upregulated cleaved caspase‐3 and Bax, while downregulated Bcl‐2 (Figure 4B). Moreover, the expression of NOX4 and NOX4‐induced apoptotic markers were reversed when the miR‐322 mimic was added to the cell (Figure 4B). In Figure 4C, compared with the control, transfection of NOX4 increased TUNEL‐positive cells, however, when a miR‐322 mimic was added, the number of TUNEL‐positive cells reduced robustly. These data suggest that miR‐322 protected cells from NOX4‐triggered apoptosis.

### 3.5 miR‐322 treatment rescues cell apoptosis in embryos with NTDs

**Figure 5:**
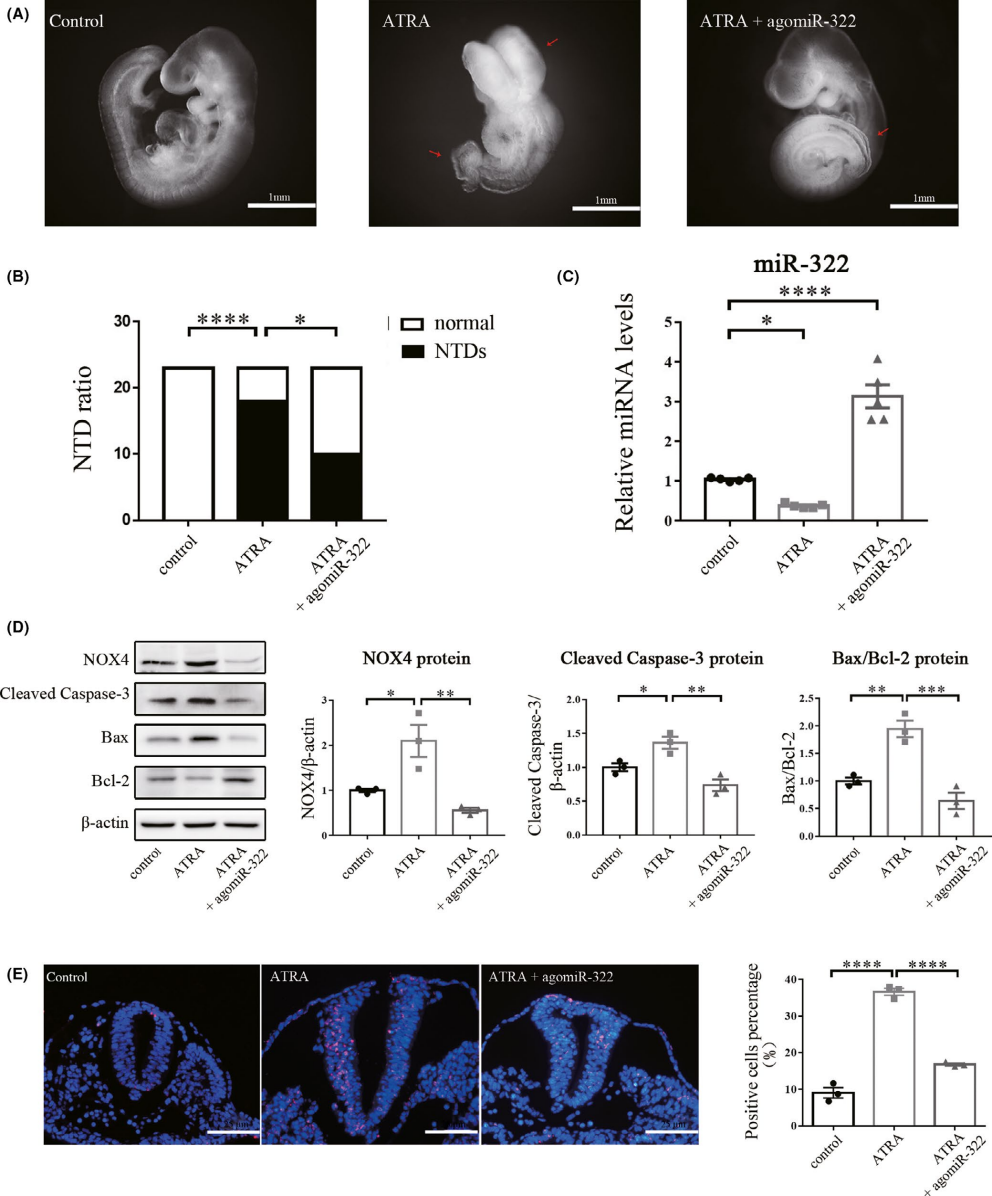
miR‐322 treatment alleviates cell apoptosis in ATRA‐induced NTD embryos. (A) Morphology of embryos from different groups after 48h of culture. (B) The number of NTDs and normally developed (normal) embryos. (C) qRT‐PCR was used to detect the expression level of miR‐322 using U6 as an internal control (n = 5). (D) Relative expression of NOX4, cleaved caspase‐3, Bax, and Bcl‐2 proteins was determined by Western blot using β‐actin as an internal control. (E) Representative images of the TUNEL assay. Apoptotic cells were labeled with TUNEL reagents, and all cell nuclei were stained by DAPI. agomiR‐322: miR‐322‐5p agomir. These assays were repeated three times. *P < .05, **P < .01, ***P < .001, ****P < .0001

To confirm the function of miR‐322/NOX4, we cultured mouse embryos at E9.5 ex vivo and then induced NTDs by adding ATRA to the culture media. Embryos were randomly divided into three groups: The first two groups were injected with negative control and cultured in serum either with or without ATRA at the same time; the last group was injected with the miR‐322‐5p agomir into the amniotic cavity and cultured in serum containing ATRA. After 48h of treatment, although obvious NTDs were observed in the groups exposed to ATRA (Figure 5A), the incidence of NTDs decreased significantly after treatment with miR‐322‐5p agomir (Figure 5B). To evaluate the effect of miR‐322‐5p agomir, we detected the expression of miR‐322 in three groups. As shown in Figure 5C, the expression of miR‐322 was decreased in NTD embryos exposed to ATRA; however, when treated with miR‐322‐5p agomir, the level of miR‐322 in the embryos was significantly increased. As a downstream target of miR‐322, we detected the protein expression of NOX4 and apoptosis‐related proteins in each group. As shown in Figure 5D, NOX4 expression was increased after addition of ATRA; expression of proapoptotic proteins including cleaved caspase‐3 and Bax was increased accordingly, while the antiapoptotic protein Bcl‐2 was reduced. However, after treatment with the miR‐322‐5p agomir, expression of NOX4, cleaved caspase‐3, and Bax decreased, while Bcl‐2 expression increased (Figure 5D). In the TUNEL assay, the addition of ATRA induced apoptosis remarkably, but after treatment with the miR‐322‐5p agomir, the number of TUNEL‐positive cells reduced obviously (Figure 5E). Summarily, these data indicate miR‐322 rescues NOX4‐induced apoptosis in NTDs caused by ATRA.

## 4 DISCUSSION

Despite advances in the mechanism and treatment of NTDs, current investment is still limited. In the present study, we provide the evidence that NOX4 is involved in apoptosis‐induced NTD formation by acting as a novel target for miR‐322. miR‐322 interacts with the 3’‐UTR of NOX4 and regulates NOX4 translation. In mouse neural stem cells, silencing NOX4 or overexpression of miR‐322 reduced apoptosis. Meanwhile, in whole‐embryo culture, miR‐322 treatment rescued cell apoptosis in NTDs by downregulating NOX4 and pro‐apoptosis markers. These results highlight the importance of miR‐322/NOX4 interplay on apoptosis in NTDs.

Apoptosis plays a vital role in biological processes, including the immune response, cell signaling, cell death, and normal embryonic development.^24^ In NTDs, cells at the neural folds and the midline of the closed neural tube undergo characteristic apoptosis.^25^, ^26^ These apoptotic cells could inhibit closure by impairing the mechanical integrity and/or functional of the neuroepithelium.^1^ The incidence of NTDs is reduced after deletion of pro‐apoptosis kinase genes, suggesting that neural tube closure failure may be associated with excessive apoptosis.^27^ Therefore, reducing apoptosis may be the key to improving the therapeutic effect of NTDs. Yang et al^27^ reported that diabetic mice treated with the ASK1 inhibitor thioredoxin, which can suppress the ASK1‐initiated apoptotic pathway, had a reduced incidence of NTDs in their embryos. Furthermore, our previous study have shown that downregulation of CITED2 mRNA and protein expression can be reversed by the miR‐200b inhibitor, which also blocked caspase‐3 cleavage and ameliorated NTDs in developing embryo.^11^ However, the mechanism of apoptosis in NTDs remains poorly understood.

miR‐322 has been reported to be associated with apoptosis. In Alzheimer's disease, miR‐322 may attenuate the apoptosis induced by aluminum maltolate, by downregulating the expression of c‐Myc.^28^ Moreover, miR‐322 plays a key role in apoptosis by directly regulating the expression of DDX3X in spermatogenesis regulation.^29^ In pancreatic cancer, miR‐322 was significantly upregulated and inhibited apoptosis by suppressing the expression of SOCS6.^30^ In our previous studies, we found that miR‐322 inhibited apoptosis via TRAF3 in high glucose‐induced neural stem cells. However, the treatment effect of miR‐322 on apoptosis in NTD embryos is lacking. Here, we injected the miR‐322 mimic into the amniotic cavity of ATRA‐induced mouse model of NTDs and found a significant reduction in apoptosis.

To explore the mechanism of miR‐322 on apoptosis in NTDs, we looked for novel targets of miR‐322. Through bioinformatic analysis, we identified NOX4 as a potential target of miR‐322. Then, we performed RNA pull‐down and dual‐luciferase assays that demonstrated miR‐322 can interacted with the 3’‐UTR of NOX4. It has been extensively reported that NOX4 is inextricably linked to apoptosis. For instance, overexpression of NOX4 significantly increased apoptosis in cultured cardiac myocytes in an age‐dependent manner, thus indicating that NOX4 has cell‐autonomous pro‐apoptotic effects.^31^ Another study showed that NOX4‐deficient mice can be protected from bleomycin‐induced pulmonary fibrosis by regulating epithelial cell death.^32^ Similarly, in mouse models of fibrosis induced by bile duct ligation or treatment with carbon tetrachloride, reduced expression of NOX4 decreased hepatocyte apoptosis.^33^, ^34^ These studies suggest that an increase in NOX4 expression contributes to apoptosis. However, whether NOX4 is involved in the occurrence of NTDs remains unknown. In our study, we demonstrate that expression of NOX4 in NTDs was significantly increased and the elevated apoptosis in mouse neural stem cell by NOX4 was also observed. These data suggest that NOX4 upregulation can promote apoptosis in NTDs, yet the mechanism by which NOX4 causes apoptosis remains unclear.

There is increasing evidence that oxidative stress can promote apoptosis.^35^, ^36^, ^37^ The central nervous system is easily damaged by oxidative attacks.^38^ For neurons with diminished levels of antioxidants, reactive oxygen species (ROS) are major contributors to disease and cell death.^39^, ^40^ In addition to the passive production of ROS by the mitochondria, NOX has been proposed to be the main source of ROS in many cell types.^41^ Thus, we hypothesize that high expression of NOX4 results in excessive ROS production, leading to oxidative stress and contributing to apoptosis in NTDs. Meanwhile, miR‐322 can prevent apoptosis by negatively regulating NOX4. Hence, we speculate that miR‐322/NOX4 reduces ROS production; then, the reduced oxidative stress in turn reduces apoptosis in NTDs. Nevertheless, more work will be required to confirm the relationship among NOX4, oxidative stress, and apoptosis in NTDs. In addition, further studies on the function of miR‐322/NOX4 in vivo in NTDs are needed.

## 5 CONCLUSIONS

Our results identified NOX4 as a direct target gene of miR‐322, investigated the role of miR‐322 and NOX4 on apoptosis, and elucidated the teratogenic effect of miR‐322/NOX4 in ATRA‐induced NTDs. Our study provides new insights into the understanding of the mechanisms of NTD formations: The deregulation of miR‐322/NOX4 triggers apoptosis, resulting in insufficient cell numbers to participate in neural tube development. Moreover, based on the treatment effect of miR‐322 on apoptosis in NTD embryos, miR‐322 may have potential as a novel target for the treatment of NTDs.

## CONFLICT OF INTEREST

The authors declare that there are no conflict of interests.

## Supporting information

Table S1

Supplementary Material
