# Supplementary material for: miR‐322 treatment rescues cell apoptosis and neural tube defect formation through silencing NADPH oxidase 4
Source: CNS Neurosci Ther. 2020 Apr 24;26(9):902–12. doi: 10.1111/cns.13383 (PMC7415201; doi:10.1111/cns.13383)
Supplement: Supplementary file 1 — Table S1 [file CNS-26-902-s001.doc]

SUPPLEMENTAL DATA

**Supplementary Table**

| Primers name | Primer sequences |  |
| --- | --- | --- |
| NOX4 F  NOX4 R | GAAGGGGTTAAACACCTCTGC  ATGCTCTGCTTAAACACAATCCT |  |
| β-actin F  β-actin R | GGAGATTACTGCCCTGGCTCCTA  GACTCATCGTACTCCTGCTTGCTG |  |
| mmu-mir-322-5p F  mmu-mir-322-5p R | AGCAGCAATTCATGTTTTGGAA  GCTGTCAACGATACGCTACGTAAC |  |
| U6 F  U6 R | CTCGCTTCGGCAGCACA  AACGCTTCACGAATTTGCGT |  |

F: forward; R: reverse.
