# Supplementary material for: miR‐322 treatment rescues cell apoptosis and neural tube defect formation through silencing NADPH oxidase 4
Source: CNS Neurosci Ther. 2020 Apr 24;26(9):902–12. doi: 10.1111/cns.13383 (PMC7415201; doi:10.1111/cns.13383)
Supplement: Supplementary file 2 — Supplementary Material [file CNS-26-902-s002.pdf]

Original western blot images:

Marker Figure

β-actin

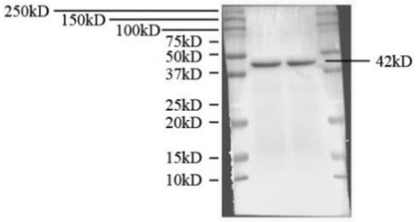

NOX4

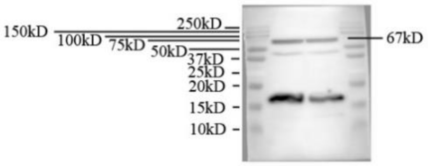

Cleaved Caspase-3

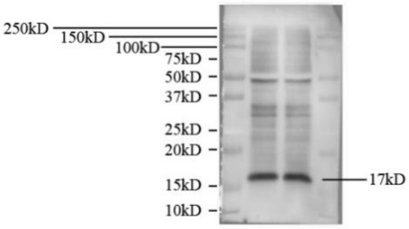

Bcl-2

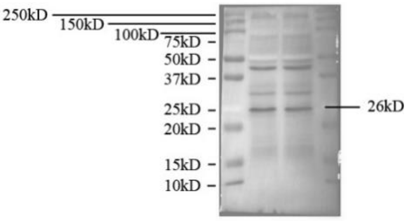

Bax

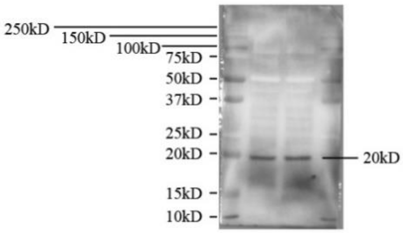

Figure1.D

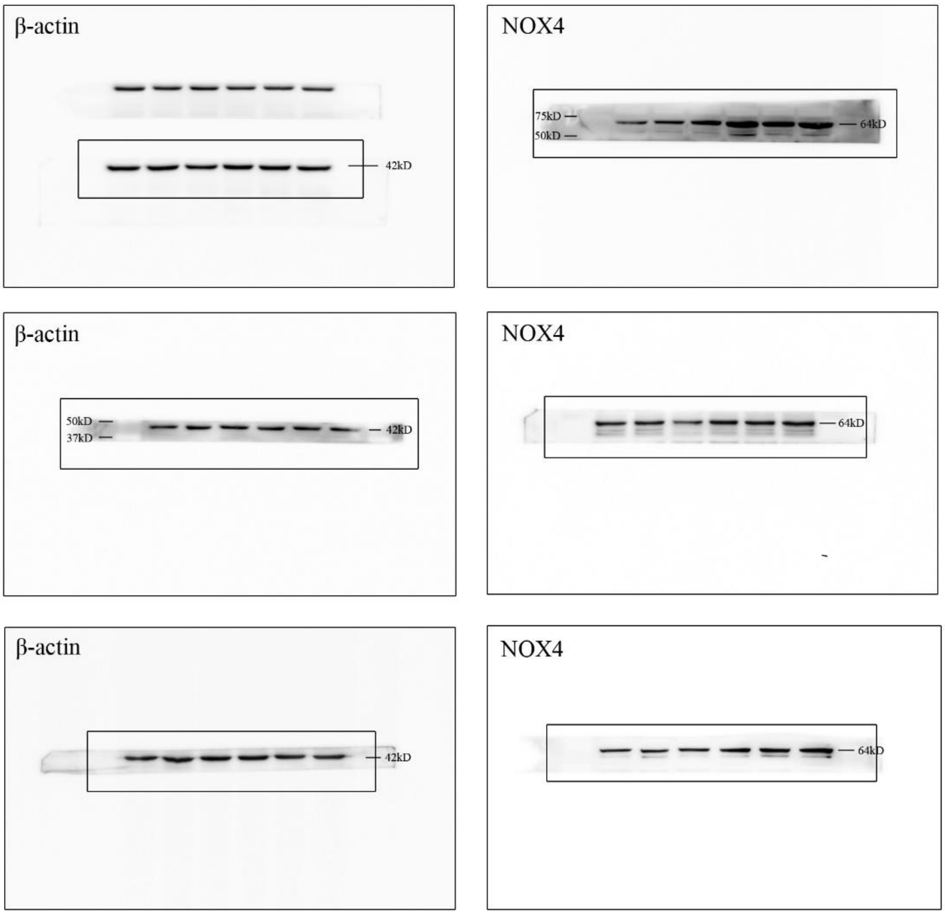
β-actin
42kD
NOX4
75kD
50kD
64kD
β-actin
50kD
37kD
42kD
NOX4
64kD
β-actin
42kD
NOX4
64kD

Figure.3.C

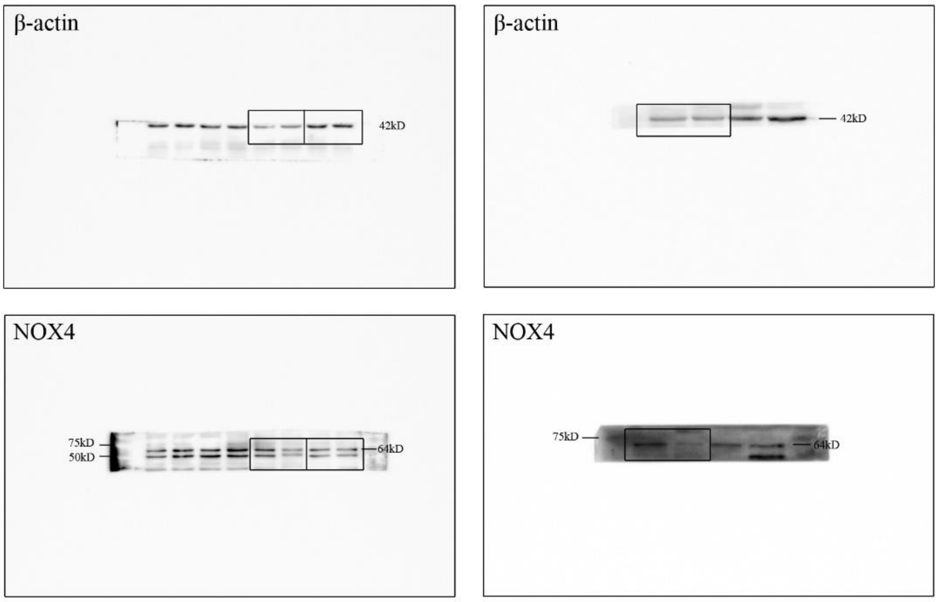

Figure.3.F

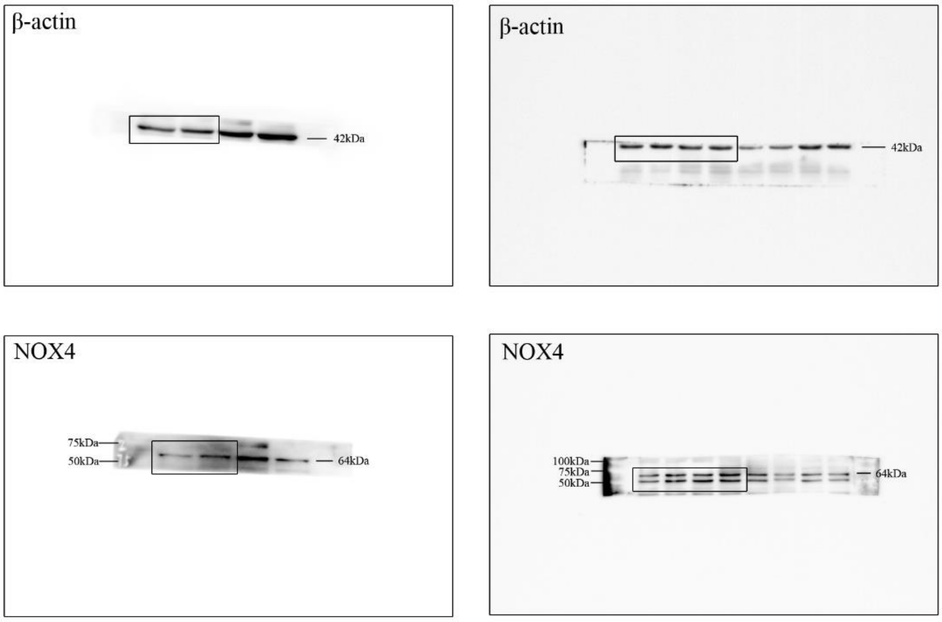
β-actin
42kDa
β-actin
42kDa
NOX4
75kDa
50kDa
64kDa
NOX4
100kDa
75kDa
50kDa
64kDa

Figure.4.A

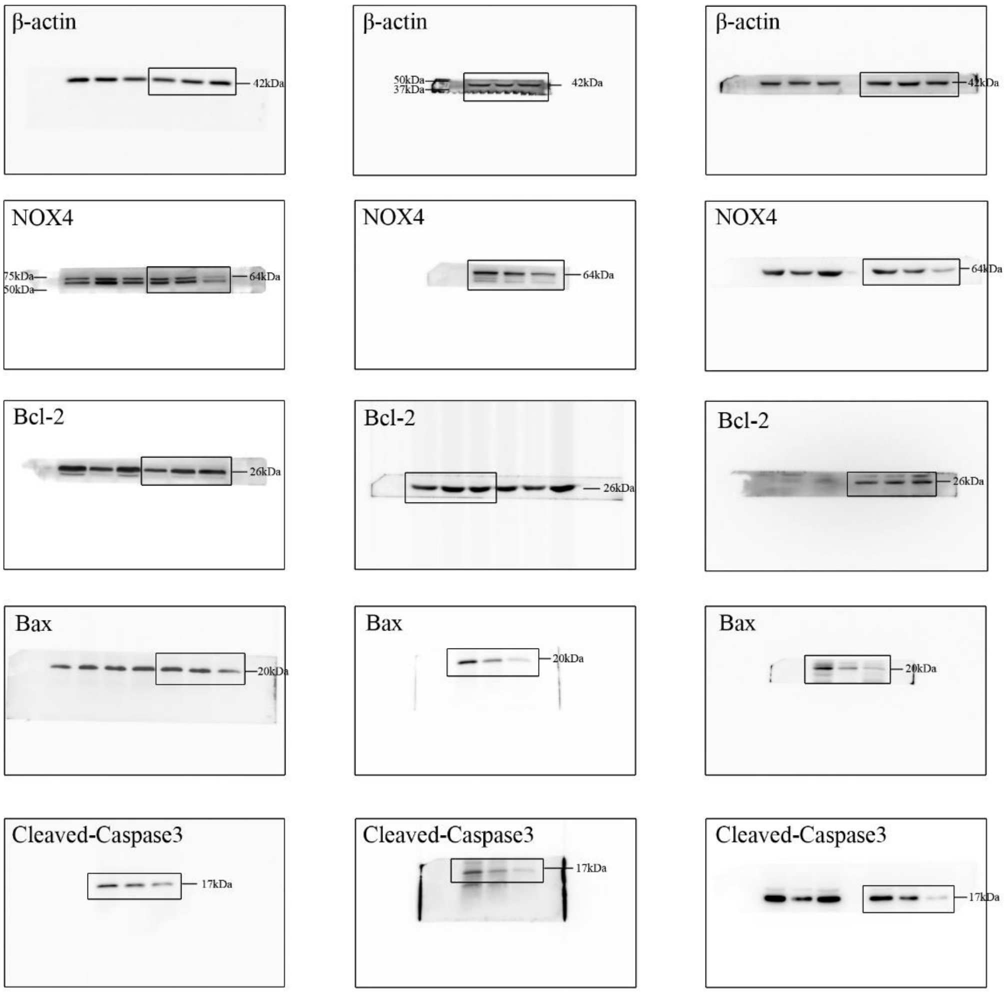
β-actin
42kDa
β-actin
50kDa
37kDa
42kDa
β-actin
42kDa
NOX4
75kDa
50kDa
64kDa
NOX4
64kDa
NOX4
64kDa
Bcl-2
26kDa
Bcl-2
26kDa
Bcl-2
26kDa
Bax
20kDa
Bax
20kDa
Bax
20kDa
Cleaved-Caspase3
17kDa
Cleaved-Caspase3
17kDa
Cleaved-Caspase3
17kDa

Figure.4.B

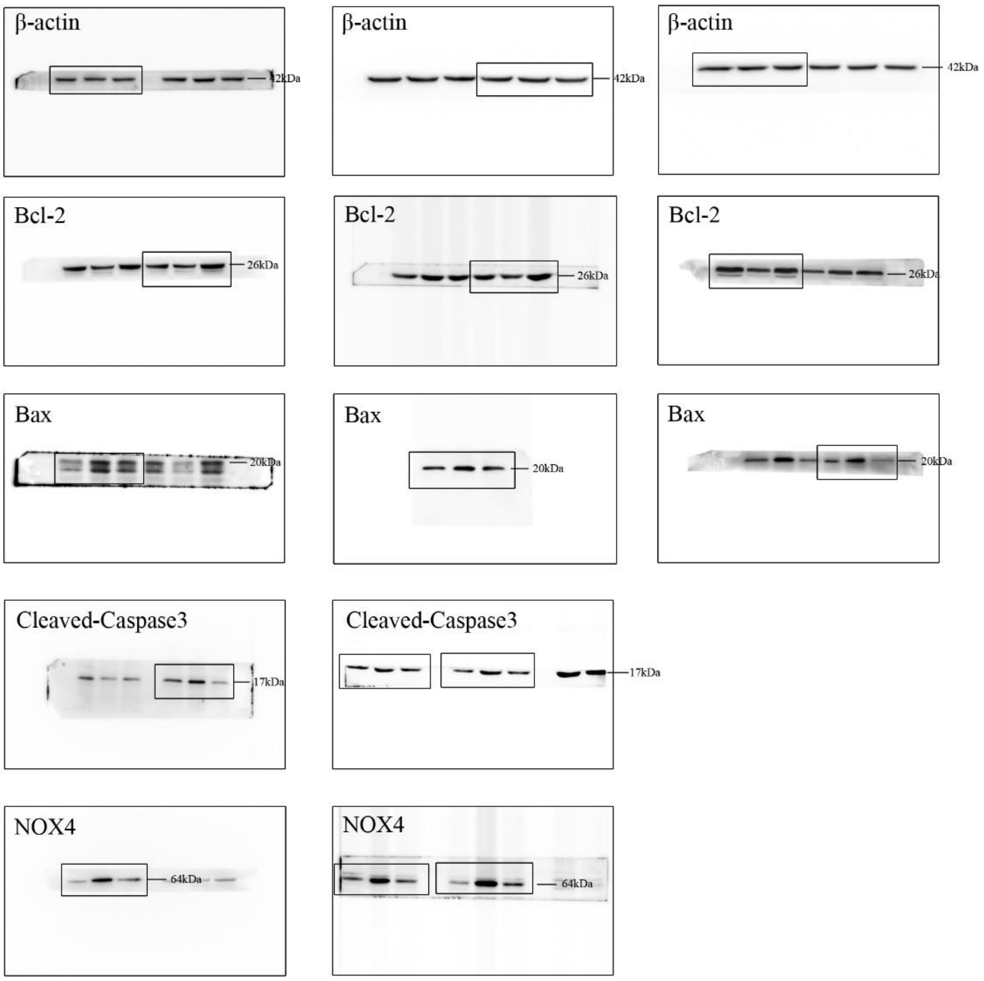
β-actin
42kDa
β-actin
42kDa
β-actin
42kDa
Bcl-2
26kDa
Bcl-2
26kDa
Bcl-2
26kDa
Bax
20kDa
Bax
20kDa
Bax
20kDa
Cleaved-Caspase3
17kDa
Cleaved-Caspase3
17kDa
NOX4
64kDa
NOX4
64kDa

Figure. 5. D

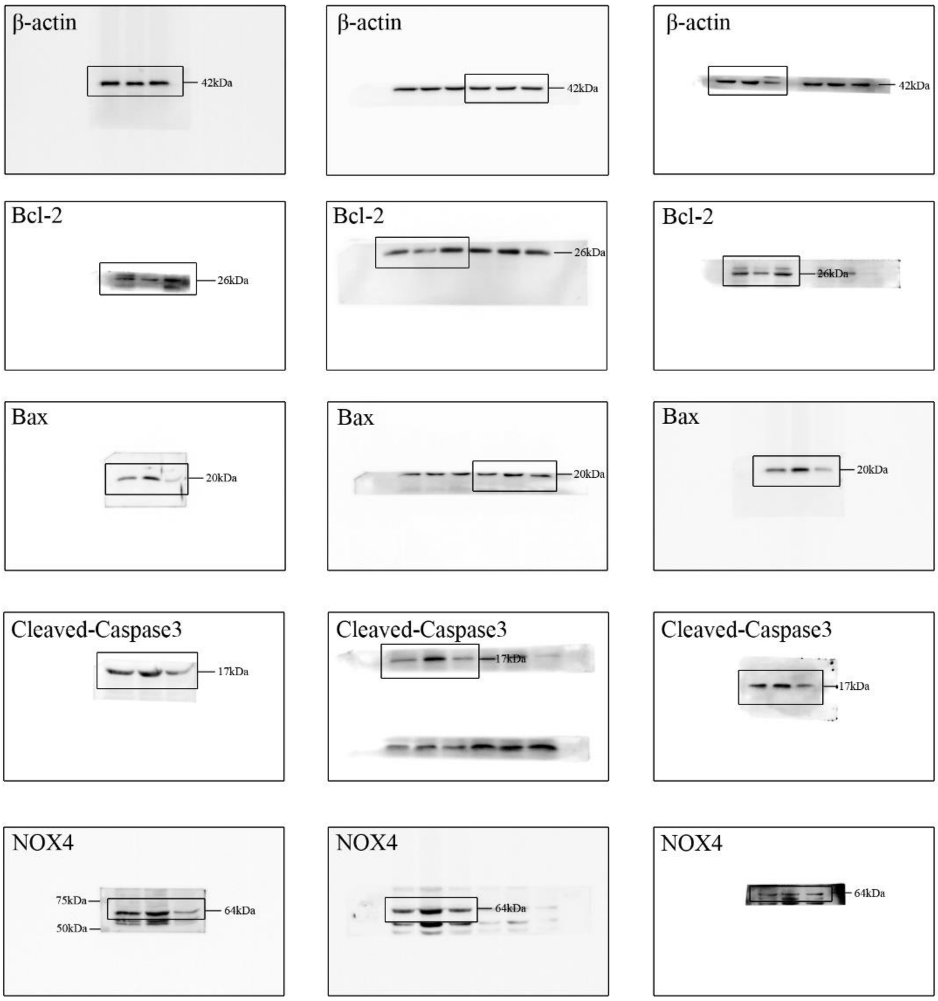
β-actin
42kDa
β-actin
42kDa
β-actin
42kDa
Bcl-2
26kDa
Bcl-2
26kDa
Bcl-2
26kDa
Bax
20kDa
Bax
20kDa
Bax
20kDa
Cleaved-Caspase3
17kDa
Cleaved-Caspase3
17kDa
Cleaved-Caspase3
17kDa
NOX4
75kDa
64kDa
50kDa
NOX4
64kDa
NOX4
64kDa

Statistic report:

Figure 1C

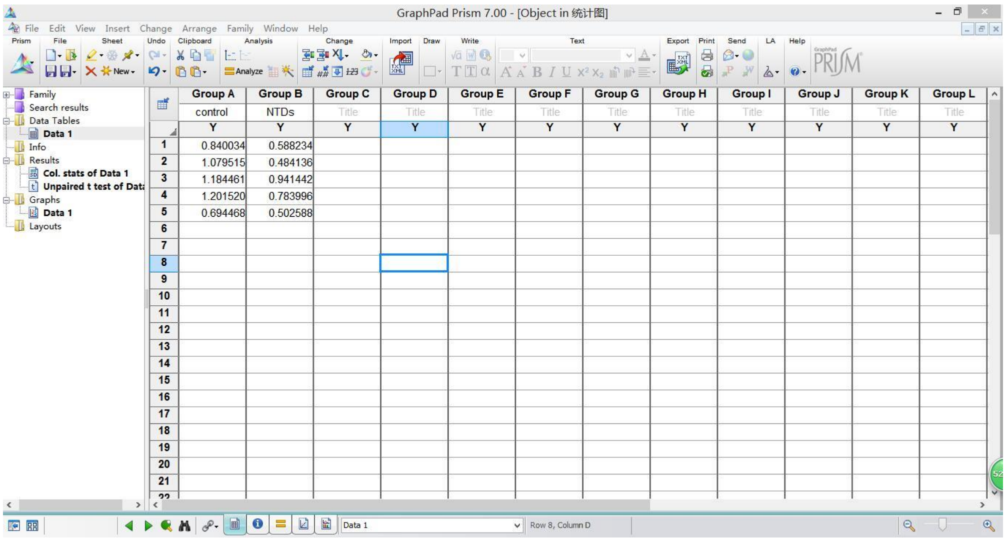

	Group A	Group B
	control	NTDs
	Y	Y
1	0.840034	0.588234
2	1.079515	0.484136
3	1.184461	0.941442
4	1.201520	0.783996
5	0.694468	0.502588

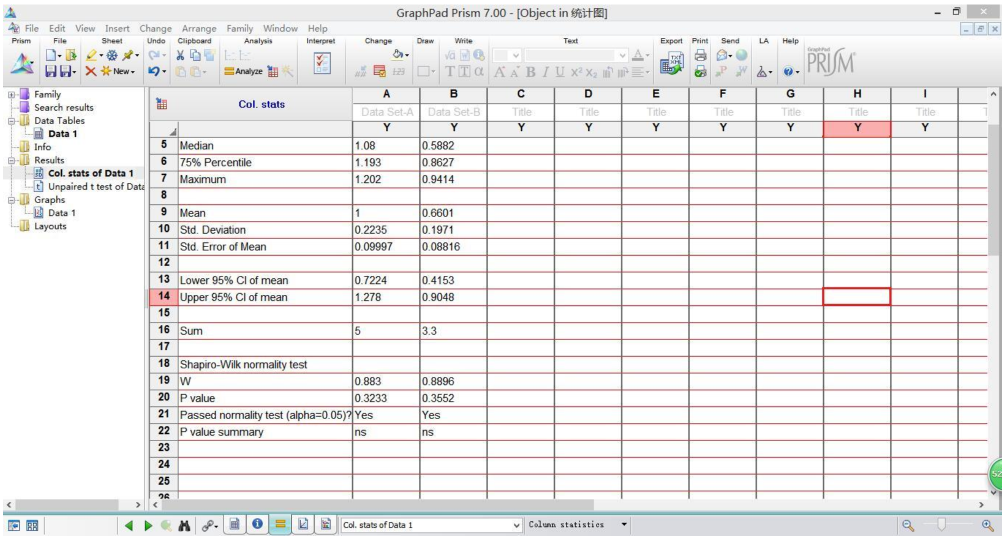

	Col. stats	A	B
		Data Set-A	Data Set-B
		Y	Y
5	Median	1.08	0.5882
6	75% Percentile	1.193	0.8627
7	Maximum	1.202	0.9414
8			
9	Mean	1	0.6601
10	Std. Deviation	0.2235	0.1971
11	Std. Error of Mean	0.09997	0.08816
12			
13	Lower 95% CI of mean	0.7224	0.4153
14	Upper 95% CI of mean	1.278	0.9048
15			
16	Sum	5	3.3
17			
18	Shapiro-Wilk normality test		
19	W	0.883	0.8896
20	P value	0.3233	0.3552
21	Passed normality test (alpha=0.05)?	Yes	Yes
22	P value summary	ns	ns

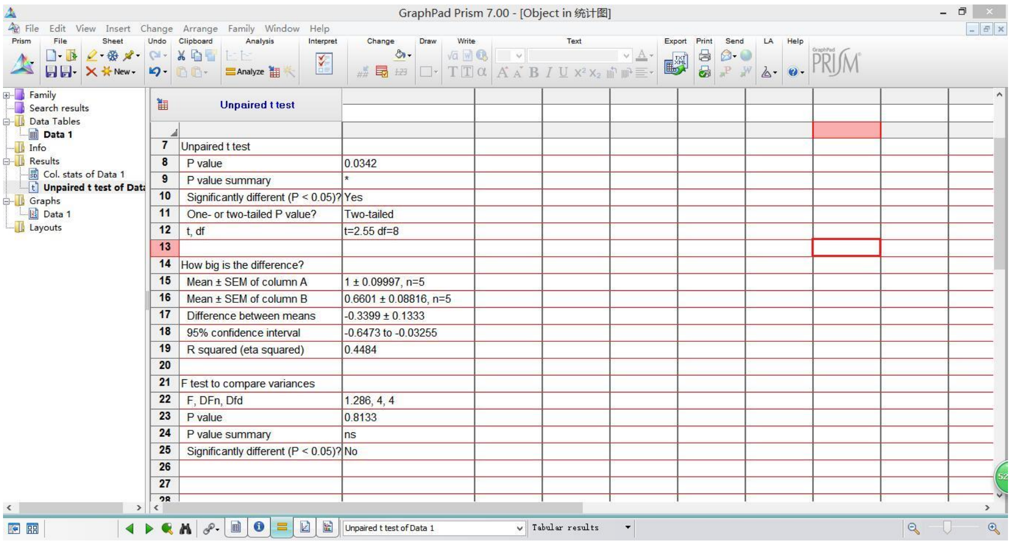

	Unpaired t test	
7	Unpaired t test	
8	P value	0.0342
9	P value summary	*
10	Significantly different (P < 0.05)?	Yes
11	One- or two-tailed P value?	Two-tailed
12	t, df	t=2.55 df=8
13		
14	How big is the difference?	
15	Mean ± SEM of column A	1 ± 0.09997, n=5
16	Mean ± SEM of column B	0.6601 ± 0.08816, n=5
17	Difference between means	-0.3399 ± 0.1333
18	95% confidence interval	-0.6473 to -0.03255
19	R squared (eta squared)	0.4484
20		
21	F test to compare variances	
22	F, DFn, Dfd	1.286, 4, 4
23	P value	0.8133
24	P value summary	ns
25	Significantly different (P < 0.05)?	No

Figure 1D

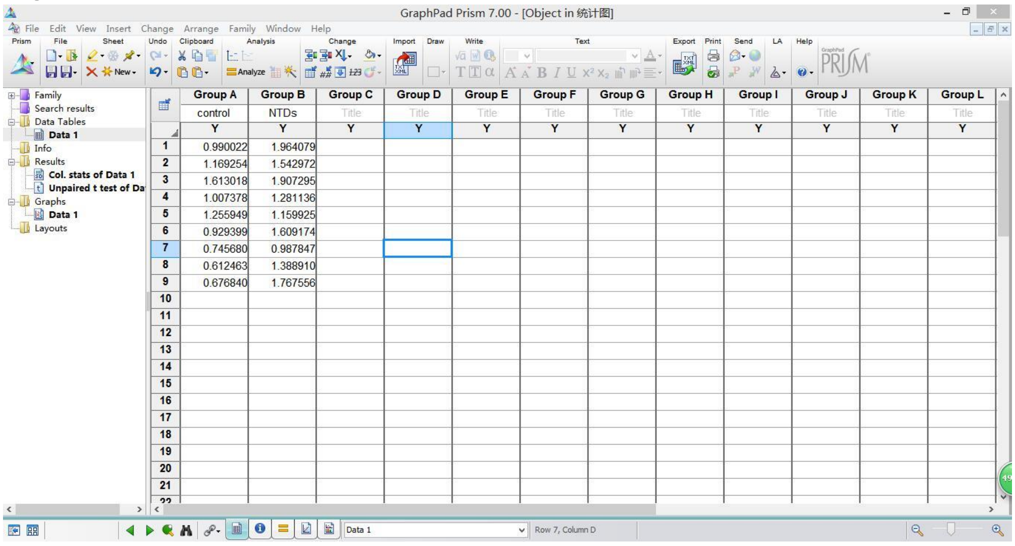

	Group A	Group B
	control	NTDs
	Y	Y
1	0.990022	1.964079
2	1.169254	1.542972
3	1.613018	1.907295
4	1.007378	1.281136
5	1.255949	1.159925
6	0.929399	1.609174
7	0.745680	0.987847
8	0.612463	1.388910
9	0.676840	1.767556

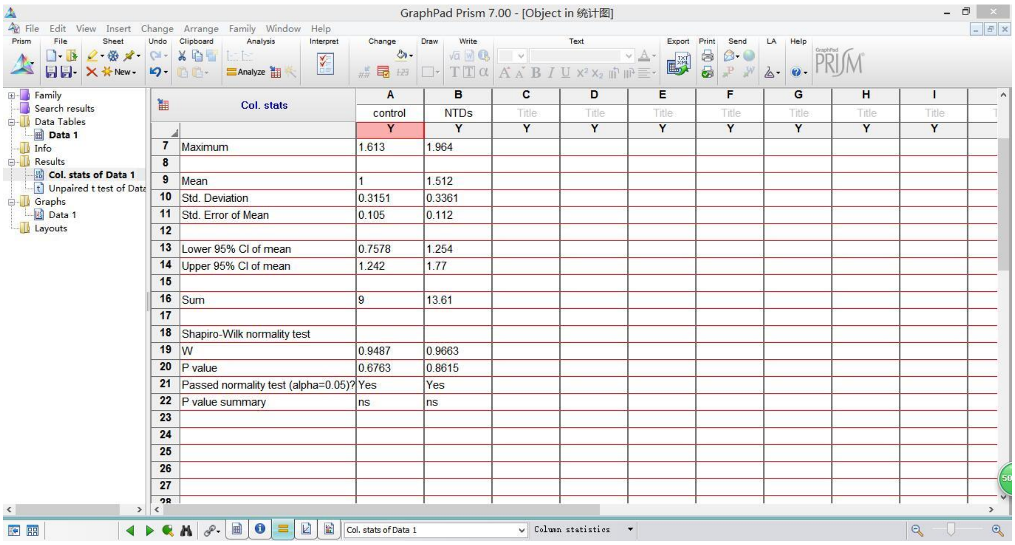

	Col. stats	A	B
		control	NTDs
		Y	Y
7	Maximum	1.613	1.964
8			
9	Mean	1	1.512
10	Std. Deviation	0.3151	0.3361
11	Std. Error of Mean	0.105	0.112
12			
13	Lower 95% CI of mean	0.7578	1.254
14	Upper 95% CI of mean	1.242	1.77
15			
16	Sum	9	13.61
17			
18	Shapiro-Wilk normality test		
19	W	0.9487	0.9663
20	P value	0.6763	0.8615
21	Passed normality test (alpha=0.05)?	Yes	Yes
22	P value summary	ns	ns

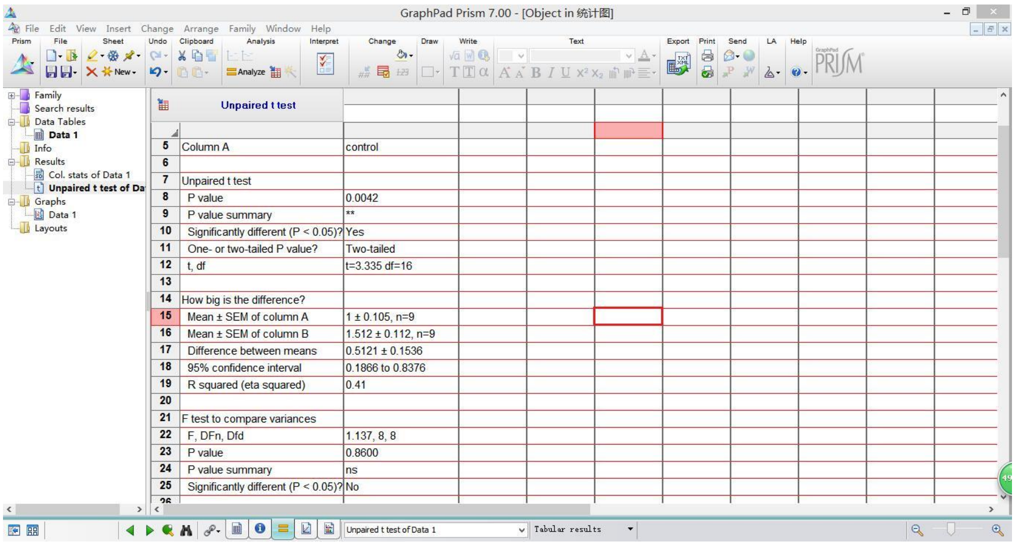

	Unpaired t test	
5	Column A	control
6		
7	Unpaired t test	
8	P value	0.0042
9	P value summary	**
10	Significantly different (P < 0.05)?	Yes
11	One- or two-tailed P value?	Two-tailed
12	t, df	t=3.335 df=16
13		
14	How big is the difference?	
15	Mean ± SEM of column A	1 ± 0.105, n=9
16	Mean ± SEM of column B	1.512 ± 0.112, n=9
17	Difference between means	0.5121 ± 0.1536
18	95% confidence interval	0.1866 to 0.8376
19	R squared (eta squared)	0.41
20		
21	F test to compare variances	
22	F, DFn, Dfd	1.137, 8, 8
23	P value	0.8600
24	P value summary	ns
25	Significantly different (P < 0.05)?	No

Figure 1E

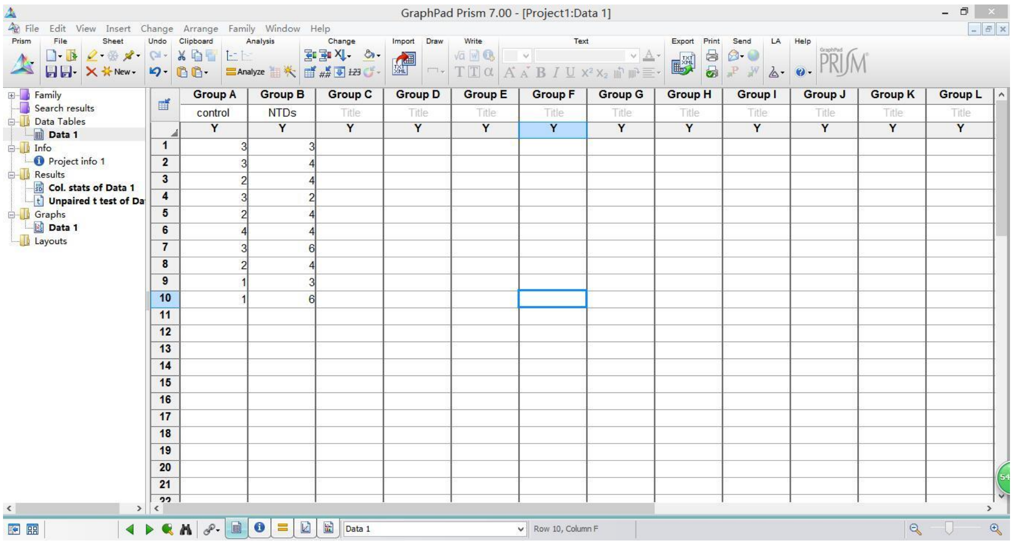

	Group A control	Group B NTDs
1	3	3
2	3	4
3	2	4
4	3	2
5	2	4
6	4	4
7	3	6
8	2	4
9	1	3
10	1	6

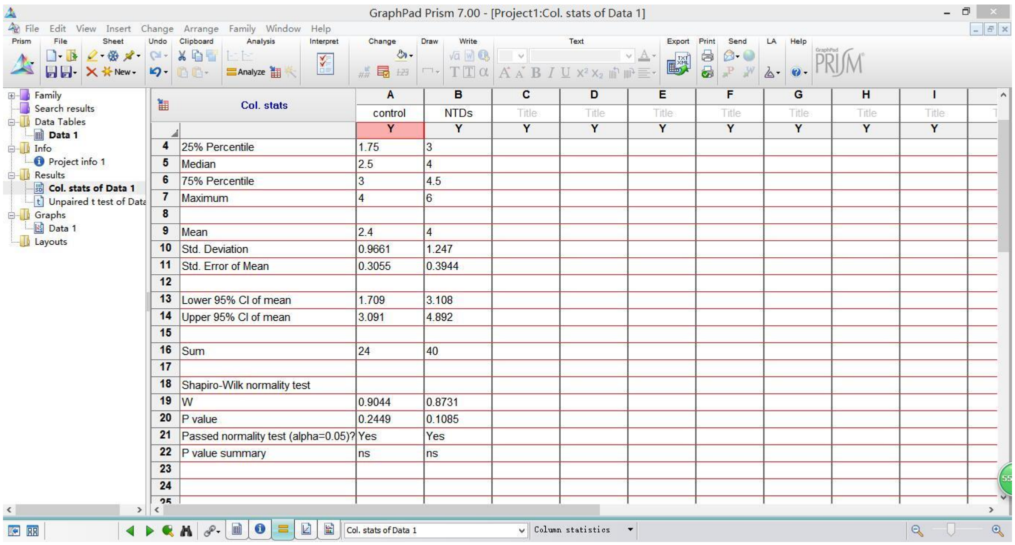

	Col. stats	A control	B NTDs
4	25% Percentile	1.75	3
5	Median	2.5	4
6	75% Percentile	3	4.5
7	Maximum	4	6
8			
9	Mean	2.4	4
10	Std. Deviation	0.9661	1.247
11	Std. Error of Mean	0.3055	0.3944
12			
13	Lower 95% CI of mean	1.709	3.108
14	Upper 95% CI of mean	3.091	4.892
15			
16	Sum	24	40
17			
18	Shapiro-Wilk normality test		
19	W	0.9044	0.8731
20	P value	0.2449	0.1085
21	Passed normality test (alpha=0.05)?	Yes	Yes
22	P value summary	ns	ns

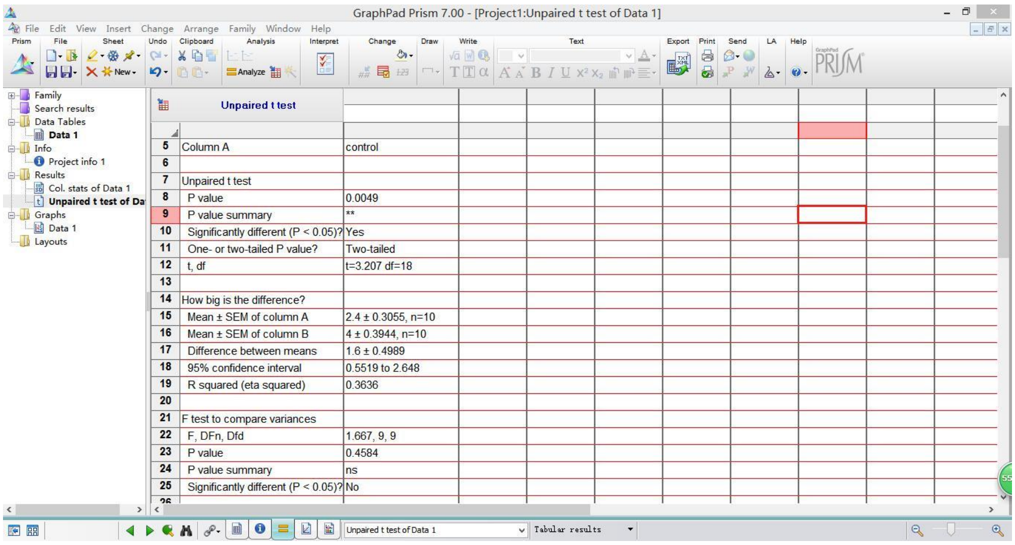

	Unpaired t test	
5	Column A	control
6		
7	Unpaired t test	
8	P value	0.0049
9	P value summary	**
10	Significantly different (P < 0.05)?	Yes
11	One- or two-tailed P value?	Two-tailed
12	t, df	t=3.207 df=18
13		
14	How big is the difference?	
15	Mean ± SEM of column A	2.4 ± 0.3055, n=10
16	Mean ± SEM of column B	4 ± 0.3944, n=10
17	Difference between means	1.6 ± 0.4989
18	95% confidence interval	0.5519 to 2.648
19	R squared (eta squared)	0.3636
20		
21	F test to compare variances	
22	F, DFn, Dfd	1.667, 9, 9
23	P value	0.4584
24	P value summary	ns
25	Significantly different (P < 0.05)?	No

Figure 1F

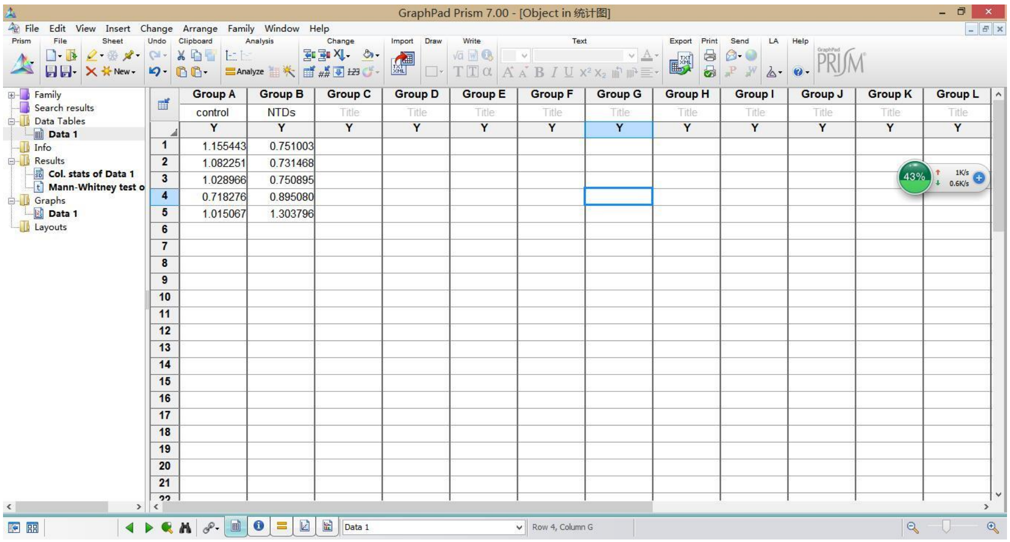
GraphPad Prism 7.00 - [Object in 统计图]
Group A
Group B
control
NTDs
1.155443
0.751003
1.082251
0.731468
1.028966
0.750895
0.718276
0.895080
1.015067
1.303796
Data 1
Row 4, Column G

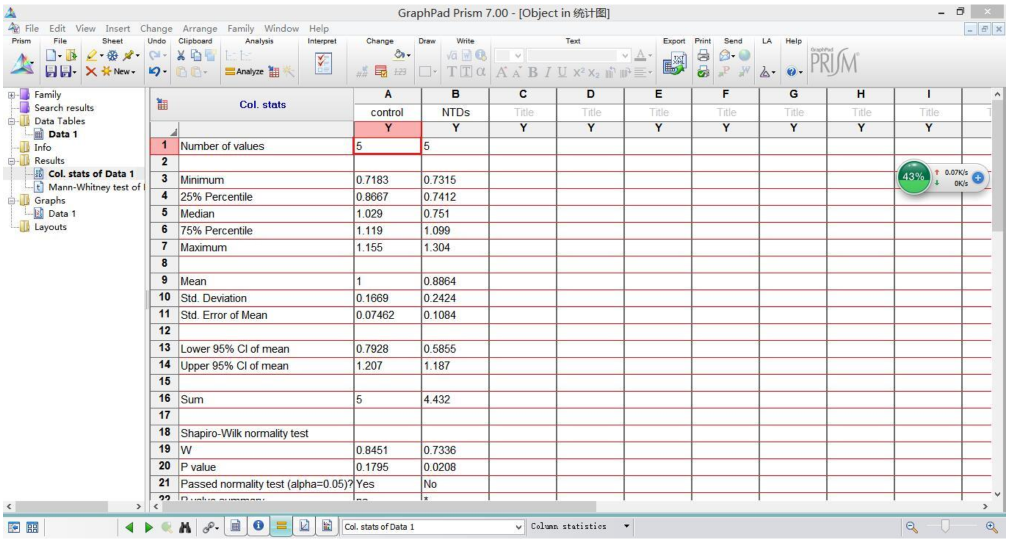
GraphPad Prism 7.00 - [Object in 统计图]
Col. stats
control
NTDs
Number of values 5 5
Minimum 0.7183 0.7315
25% Percentile 0.8667 0.7412
Median 1.029 0.751
75% Percentile 1.119 1.099
Maximum 1.155 1.304
Mean 1 0.8864
Std. Deviation 0.1669 0.2424
Std. Error of Mean 0.07462 0.1084
Lower 95% CI of mean 0.7928 0.5855
Upper 95% CI of mean 1.207 1.187
Sum 5 4.432
Shapiro-Wilk normality test
W 0.8451 0.7336
P value 0.1795 0.0208
Passed normality test (alpha=0.05)? Yes No
Col. stats of Data 1
Column statistics

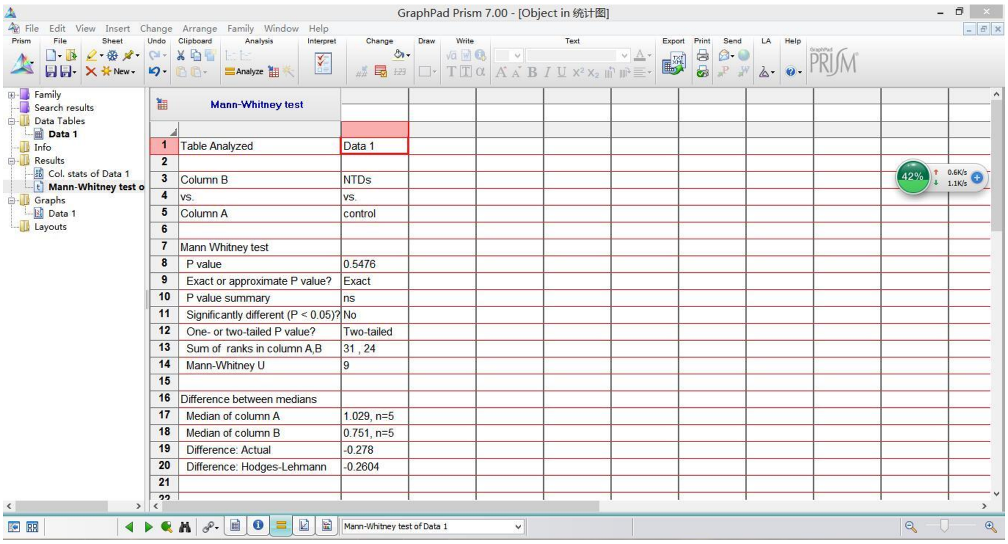
GraphPad Prism 7.00 - [Object in 统计图]
Mann-Whitney test
Table Analyzed Data 1
Column B NTDs
vs. vs.
Column A control
Mann Whitney test
P value 0.5476
Exact or approximate P value? Exact
P value summary ns
Significantly different (P < 0.05)? No
One- or two-tailed P value? Two-tailed
Sum of ranks in column A,B 31 , 24
Mann-Whitney U 9
Difference between medians
Median of column A 1.029, n=5
Median of column B 0.751, n=5
Difference: Actual -0.278
Difference: Hodges-Lehmann -0.2604
Mann-Whitney test of Data 1

Figure 1G

Relative fluorescence intensity:

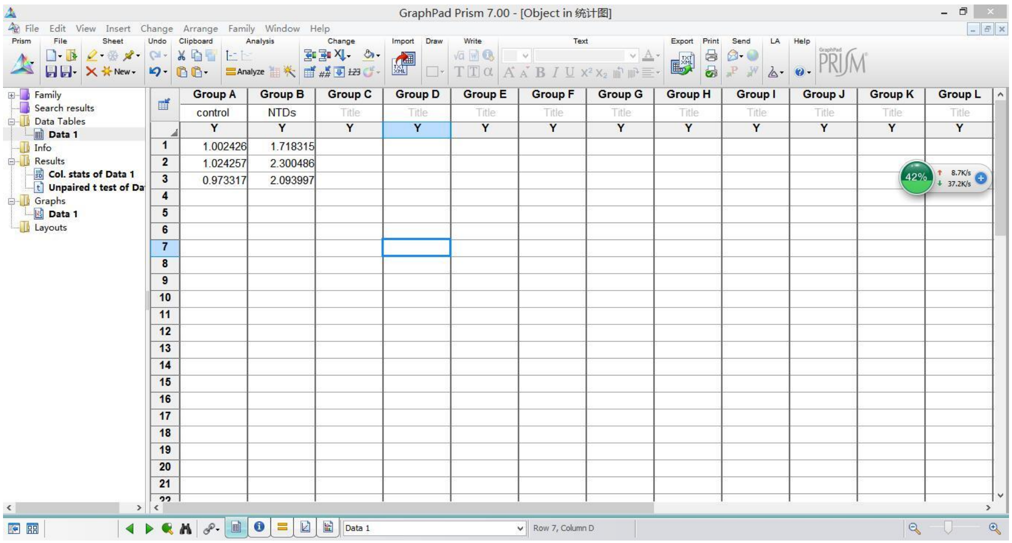

GraphPad Prism 7.00 - [Object in 统计图]

	Group A	Group B
	control	NTDs
	Y	Y
1	1.002426	1.718315
2	1.024257	2.300486
3	0.973317	2.093997

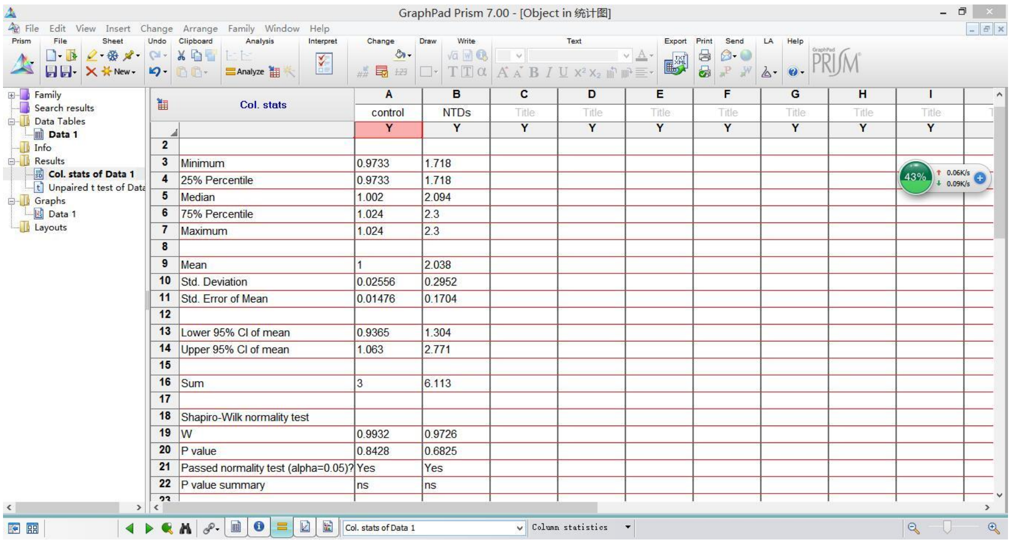

GraphPad Prism 7.00 - [Object in 统计图]

Col. stats	A	B
	control	NTDs
	Y	Y
3 Minimum	0.9733	1.718
4 25% Percentile	0.9733	1.718
5 Median	1.002	2.094
6 75% Percentile	1.024	2.3
7 Maximum	1.024	2.3
9 Mean	1	2.038
10 Std. Deviation	0.02556	0.2952
11 Std. Error of Mean	0.01476	0.1704
13 Lower 95% CI of mean	0.9365	1.304
14 Upper 95% CI of mean	1.063	2.771
16 Sum	3	6.113
18 Shapiro-Wilk normality test		
19 W	0.9932	0.9726
20 P value	0.8428	0.6825
21 Passed normality test (alpha=0.05)?	Yes	Yes
22 P value summary	ns	ns

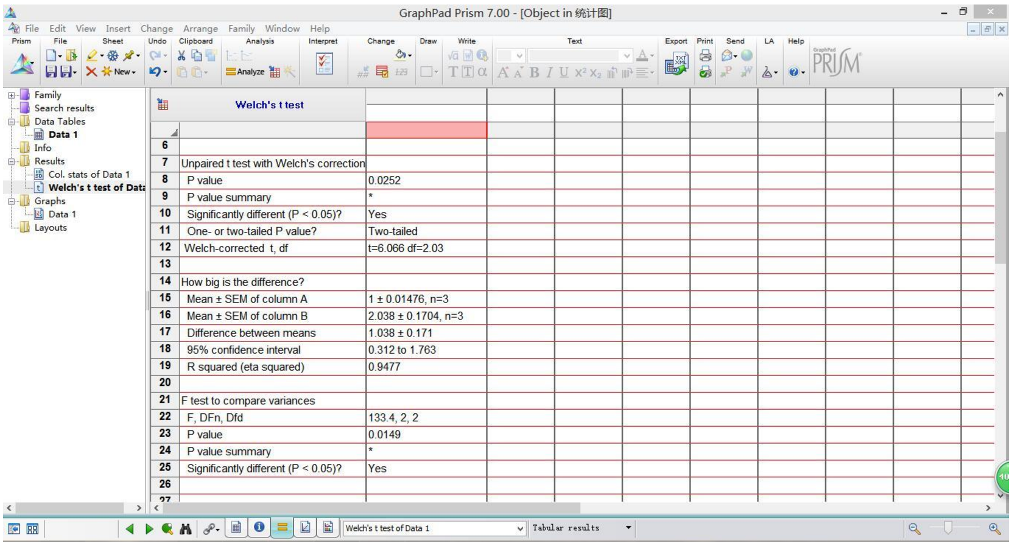

GraphPad Prism 7.00 - [Object in 统计图]

Welch's t test	
7 Unpaired t test with Welch's correction	
8 P value	0.0252
9 P value summary	*
10 Significantly different (P < 0.05)?	Yes
11 One- or two-tailed P value?	Two-tailed
12 Welch-corrected t, df	t=6.066 df=2.03
14 How big is the difference?	
15 Mean ± SEM of column A	1 ± 0.01476, n=3
16 Mean ± SEM of column B	2.038 ± 0.1704, n=3
17 Difference between means	1.038 ± 0.171
18 95% confidence interval	0.312 to 1.763
19 R squared (eta squared)	0.9477
21 F test to compare variances	
22 F, DFn, Dfd	133.4, 2, 2
23 P value	0.0149
24 P value summary	*
25 Significantly different (P < 0.05)?	Yes

TUNEL signal

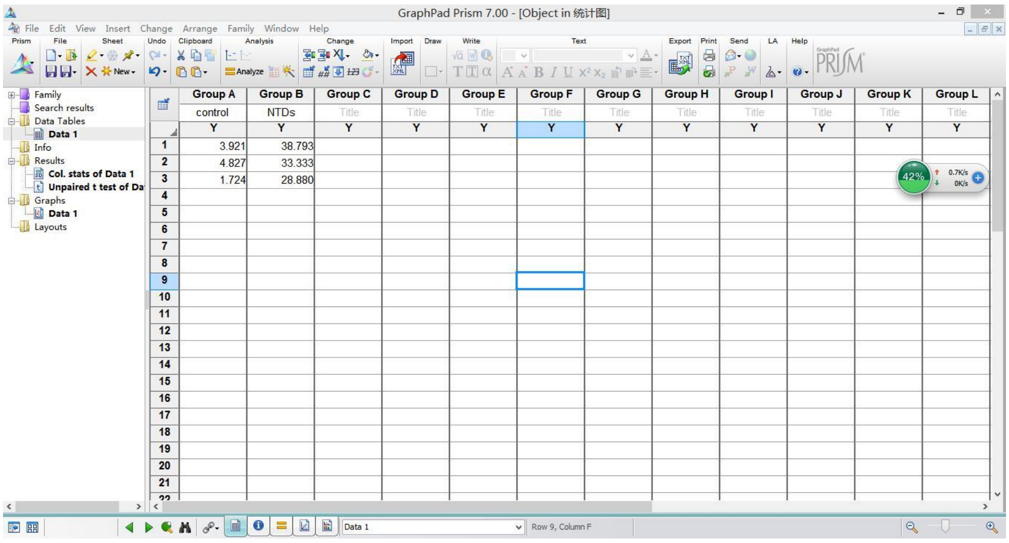

	Group A	Group B
	control	NTDs
	Y	Y
1	3.921	38.793
2	4.827	33.333
3	1.724	28.880

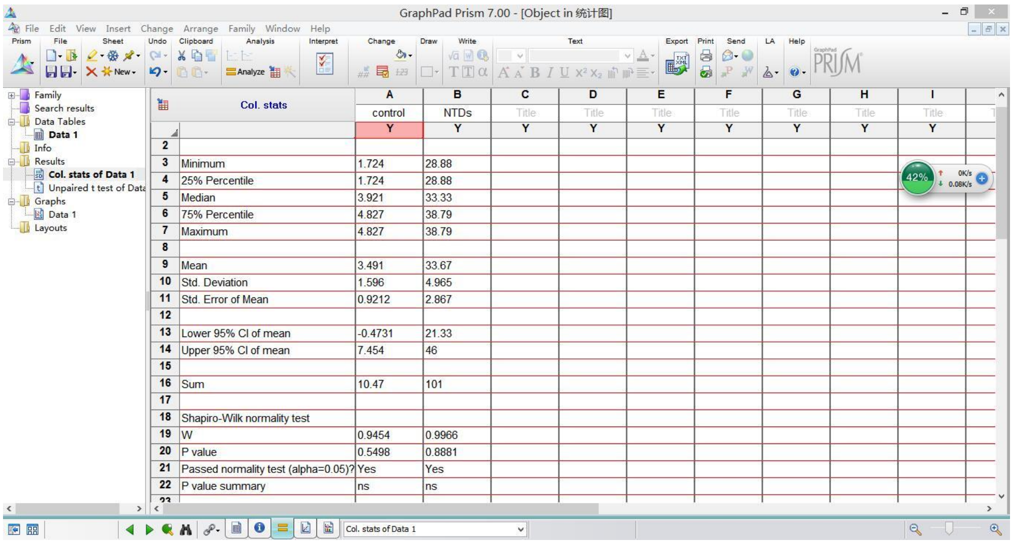

	Col. stats	A	B
		control	NTDs
		Y	Y
3	Minimum	1.724	28.88
4	25% Percentile	1.724	28.88
5	Median	3.921	33.33
6	75% Percentile	4.827	38.79
7	Maximum	4.827	38.79
9	Mean	3.491	33.67
10	Std. Deviation	1.596	4.965
11	Std. Error of Mean	0.9212	2.867
13	Lower 95% CI of mean	-0.4731	21.33
14	Upper 95% CI of mean	7.454	46
16	Sum	10.47	101
18	Shapiro-Wilk normality test		
19	W	0.9454	0.9966
20	P value	0.5498	0.8881
21	Passed normality test (alpha=0.05)?	Yes	Yes
22	P value summary	ns	ns

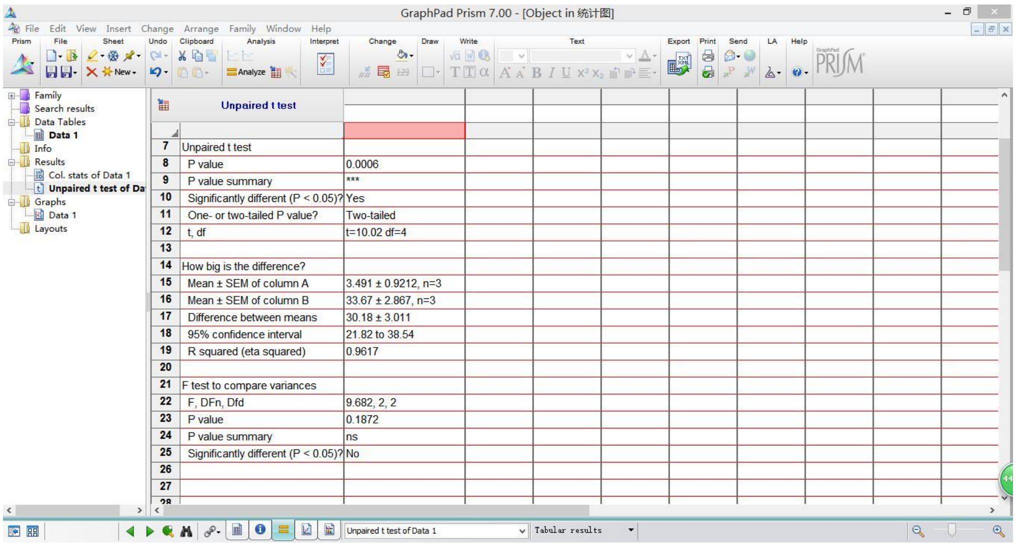

	Unpaired t test	
7	Unpaired t test	
8	P value	0.0006
9	P value summary	***
10	Significantly different (P < 0.05)?	Yes
11	One- or two-tailed P value?	Two-tailed
12	t, df	t=10.02 df=4
14	How big is the difference?	
15	Mean ± SEM of column A	3.491 ± 0.9212, n=3
16	Mean ± SEM of column B	33.67 ± 2.867, n=3
17	Difference between means	30.18 ± 3.011
18	95% confidence interval	21.82 to 38.54
19	R squared (eta squared)	0.9617
21	F test to compare variances	
22	F, DFn, Dfd	9.682, 2, 2
23	P value	0.1872
24	P value summary	ns
25	Significantly different (P < 0.05)?	No

Figure 2B

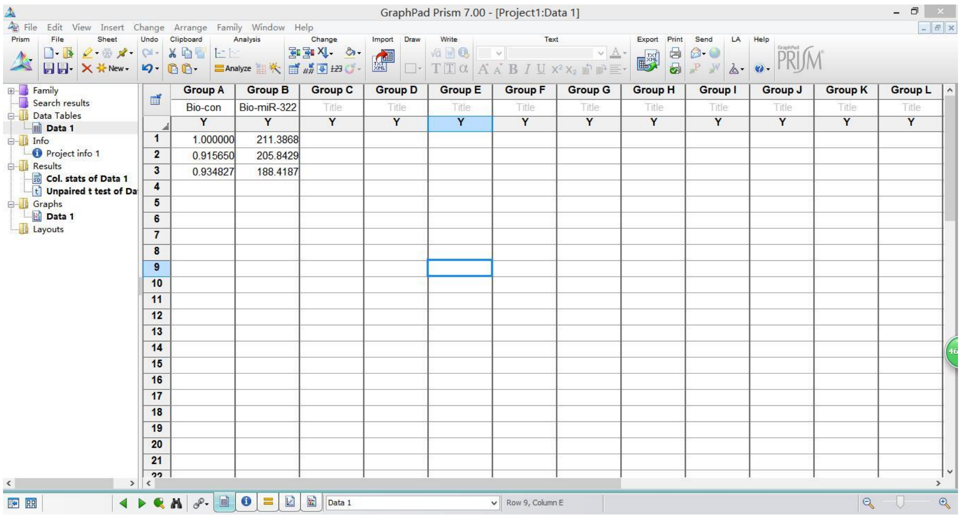
GraphPad Prism 7.00 - [Project1:Data 1]
Group A
Group B
Bio-con
Bio-miR-322
1.000000
211.3868
0.915650
205.8429
0.934827
188.4187

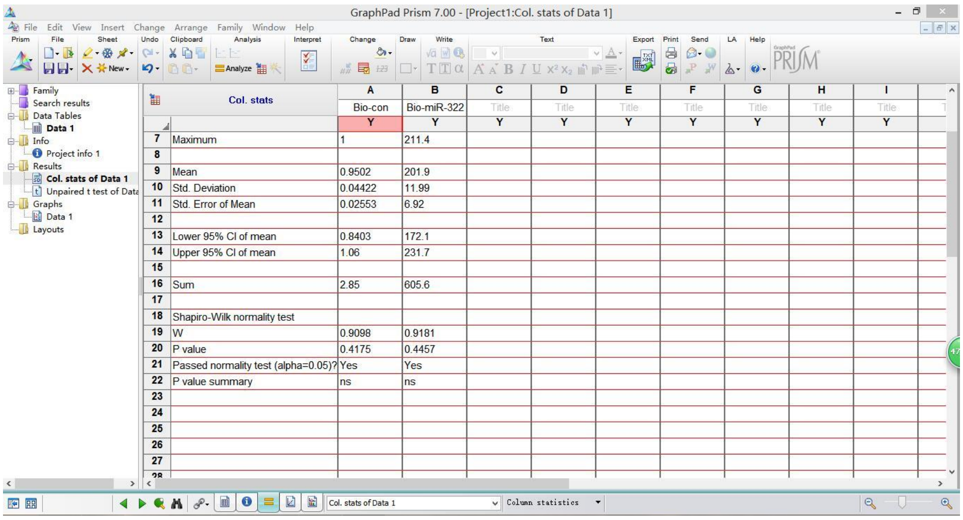
GraphPad Prism 7.00 - [Project1:Col. stats of Data 1]
Col. stats
Bio-con
Bio-miR-322
Maximum 1 211.4
Mean 0.9502 201.9
Std. Deviation 0.04422 11.99
Std. Error of Mean 0.02553 6.92
Lower 95% CI of mean 0.8403 172.1
Upper 95% CI of mean 1.06 231.7
Sum 2.85 605.6
Shapiro-Wilk normality test
W 0.9098 0.9181
P value 0.4175 0.4457
Passed normality test (alpha=0.05)? Yes Yes
P value summary ns ns

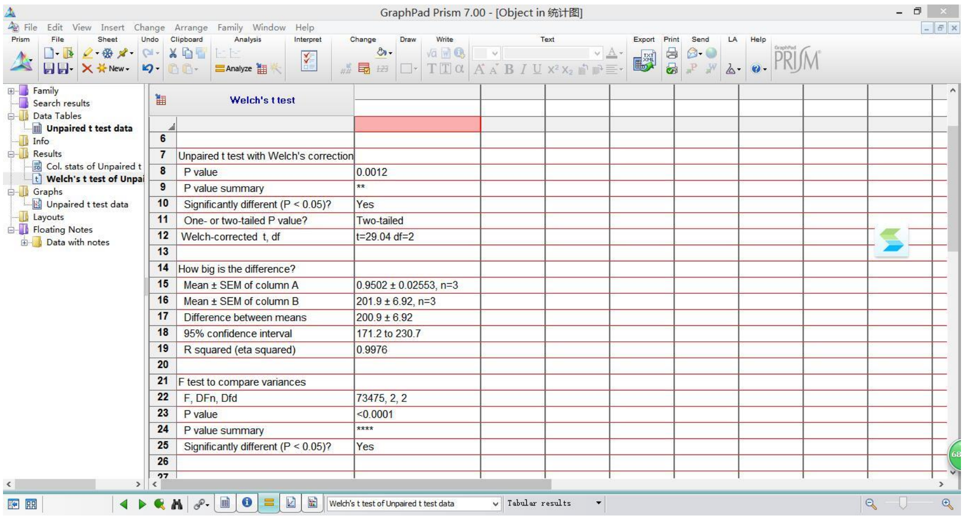
GraphPad Prism 7.00 - [Object in 统计图]
Welch's t test
Unpaired t test with Welch's correction
P value 0.0012
P value summary **
Significantly different (P < 0.05)? Yes
One- or two-tailed P value? Two-tailed
Welch-corrected t, df t=29.04 df=2
How big is the difference?
Mean ± SEM of column A 0.9502 ± 0.02553, n=3
Mean ± SEM of column B 201.9 ± 6.92, n=3
Difference between means 200.9 ± 6.92
95% confidence interval 171.2 to 230.7
R squared (eta squared) 0.9976
F test to compare variances
F, DFn, Dfd 73475, 2, 2
P value <0.0001
P value summary ****
Significantly different (P < 0.05)? Yes

Figure 2C

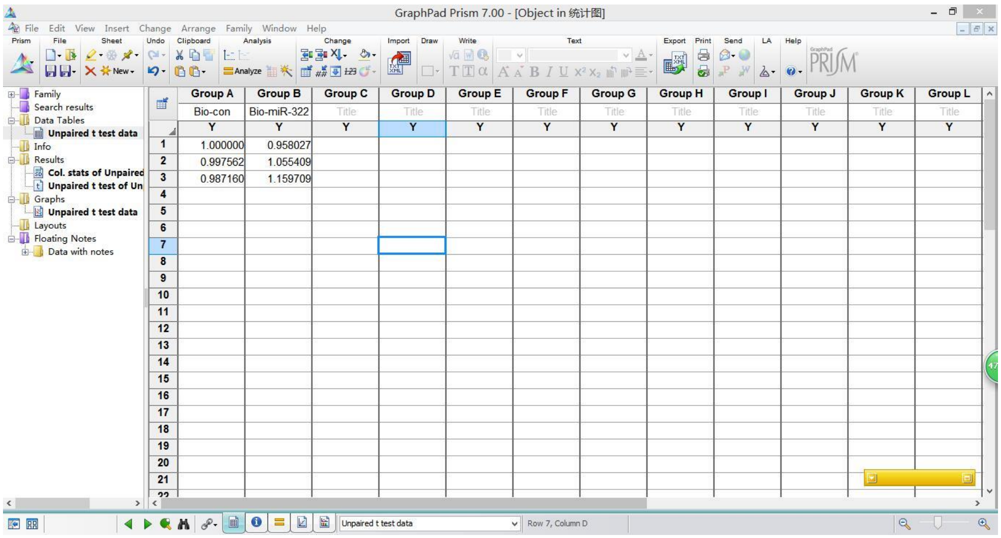

	Group A	Group B
	Bio-con	Bio-miR-322
	Y	Y
1	1.000000	0.958027
2	0.997562	1.055409
3	0.987160	1.159709

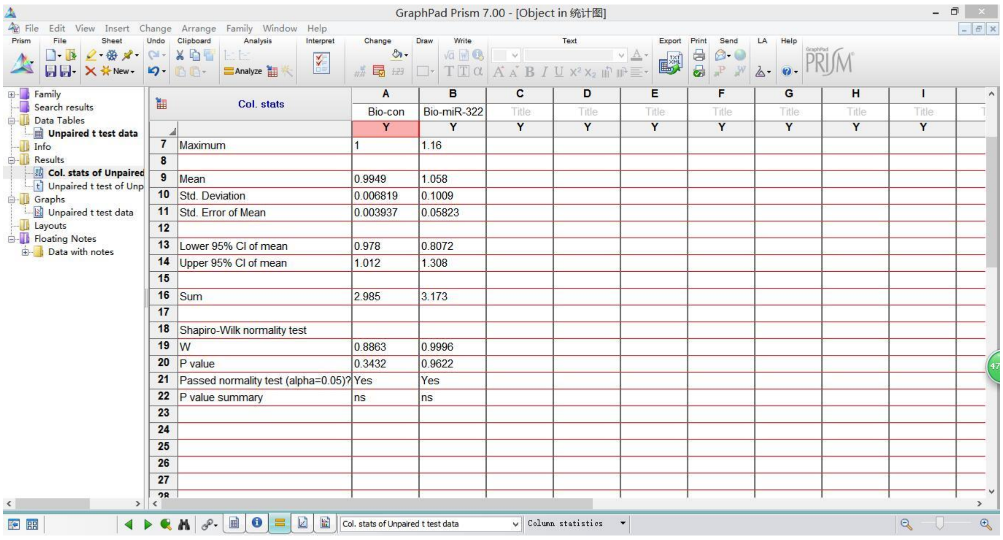

	Col. stats	A	B
		Bio-con	Bio-miR-322
7	Maximum	1	1.16
8			
9	Mean	0.9949	1.058
10	Std. Deviation	0.006819	0.1009
11	Std. Error of Mean	0.003937	0.05823
12			
13	Lower 95% CI of mean	0.978	0.8072
14	Upper 95% CI of mean	1.012	1.308
15			
16	Sum	2.985	3.173
17			
18	Shapiro-Wilk normality test		
19	W	0.8863	0.9996
20	P value	0.3432	0.9622
21	Passed normality test (alpha=0.05)?	Yes	Yes
22	P value summary	ns	ns

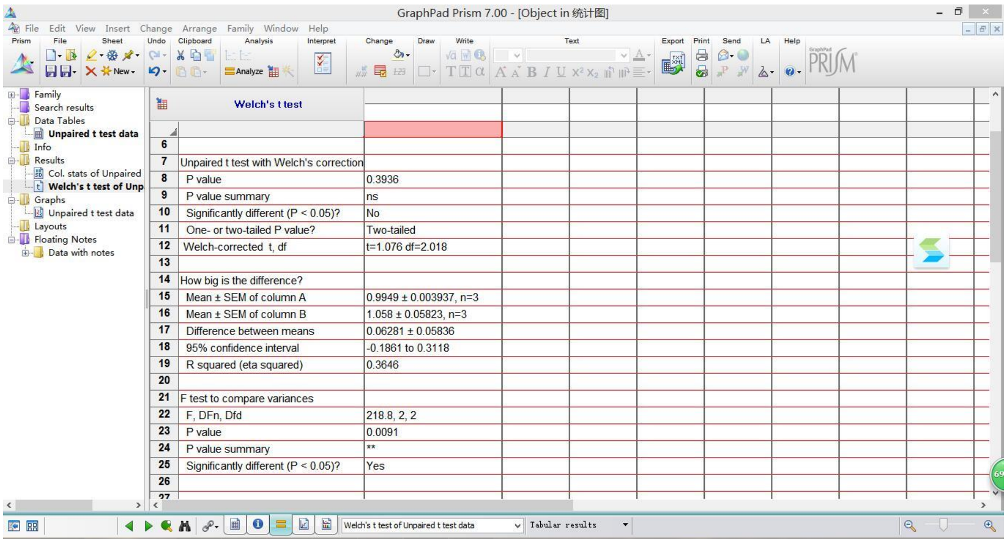

	Welch's t test	
7	Unpaired t test with Welch's correction	
8	P value	0.3936
9	P value summary	ns
10	Significantly different (P < 0.05)?	No
11	One- or two-tailed P value?	Two-tailed
12	Welch-corrected t, df	t=1.076 df=2.018
13		
14	How big is the difference?	
15	Mean ± SEM of column A	0.9949 ± 0.003937, n=3
16	Mean ± SEM of column B	1.058 ± 0.05823, n=3
17	Difference between means	0.06281 ± 0.05836
18	95% confidence interval	-0.1861 to 0.3118
19	R squared (eta squared)	0.3646
20		
21	F test to compare variances	
22	F, DFn, Dfd	218.8, 2, 2
23	P value	0.0091
24	P value summary	**
25	Significantly different (P < 0.05)?	Yes

Figure 2D

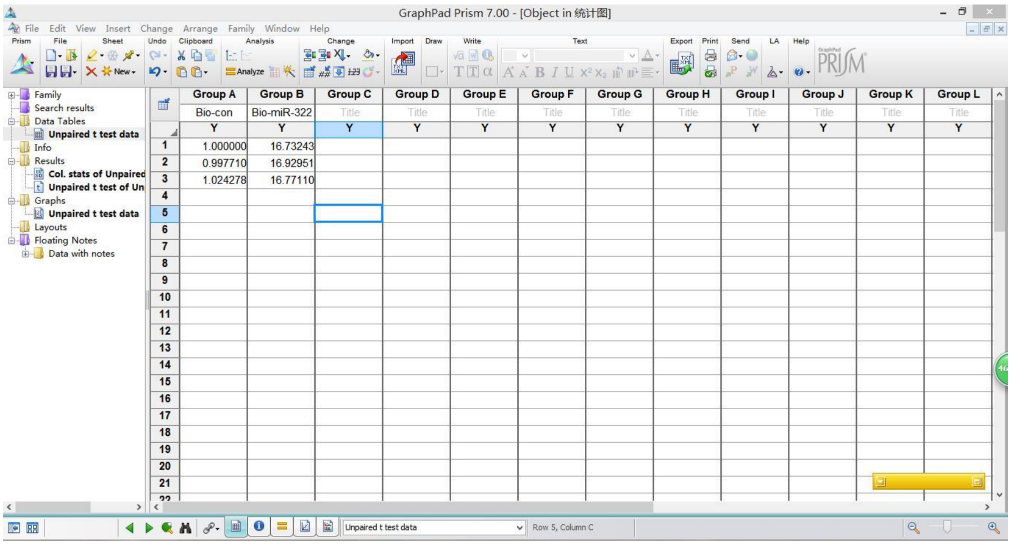

GraphPad Prism 7.00 - [Object in 统计图]
Family
Search results
Data Tables
Unpaired t test data
Info
Results
Col. stats of Unpaired
Unpaired t test of Un
Graphs
Unpaired t test data
Layouts
Floating Notes
Data with notes
Group A
Bio-con
Group B
Bio-miR-322
1 1.000000 16.73243
2 0.997710 16.92951
3 1.024278 16.77110
Unpaired t test data
Row 5, Column C

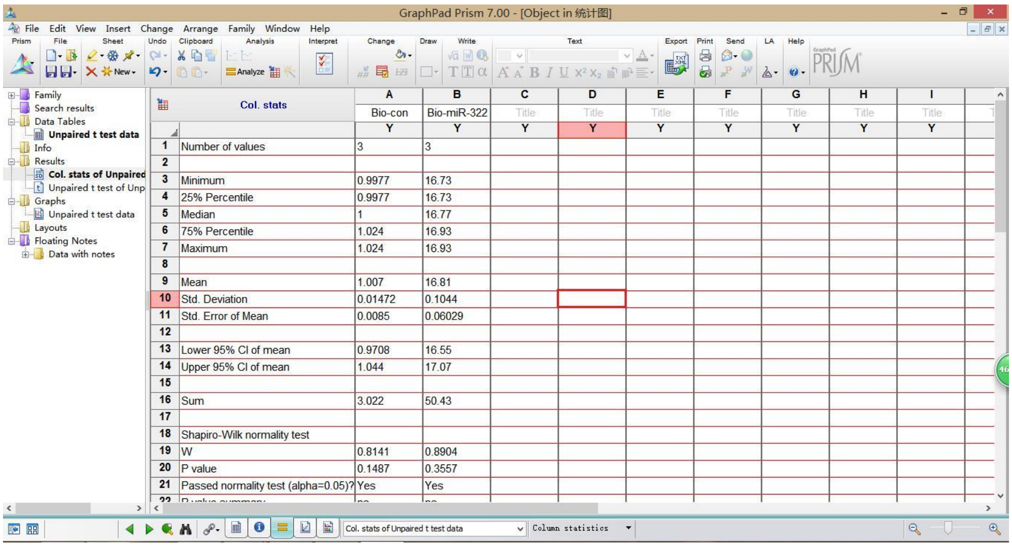

GraphPad Prism 7.00 - [Object in 统计图]
Col. stats
A Bio-con
B Bio-miR-322
1 Number of values 3 3
3 Minimum 0.9977 16.73
4 25% Percentile 0.9977 16.73
5 Median 1 16.77
6 75% Percentile 1.024 16.93
7 Maximum 1.024 16.93
9 Mean 1.007 16.81
10 Std. Deviation 0.01472 0.1044
11 Std. Error of Mean 0.0085 0.06029
13 Lower 95% CI of mean 0.9708 16.55
14 Upper 95% CI of mean 1.044 17.07
16 Sum 3.022 50.43
18 Shapiro-Wilk normality test
19 W 0.8141 0.8904
20 P value 0.1487 0.3557
21 Passed normality test (alpha=0.05)? Yes Yes
Col. stats of Unpaired t test data
Column statistics

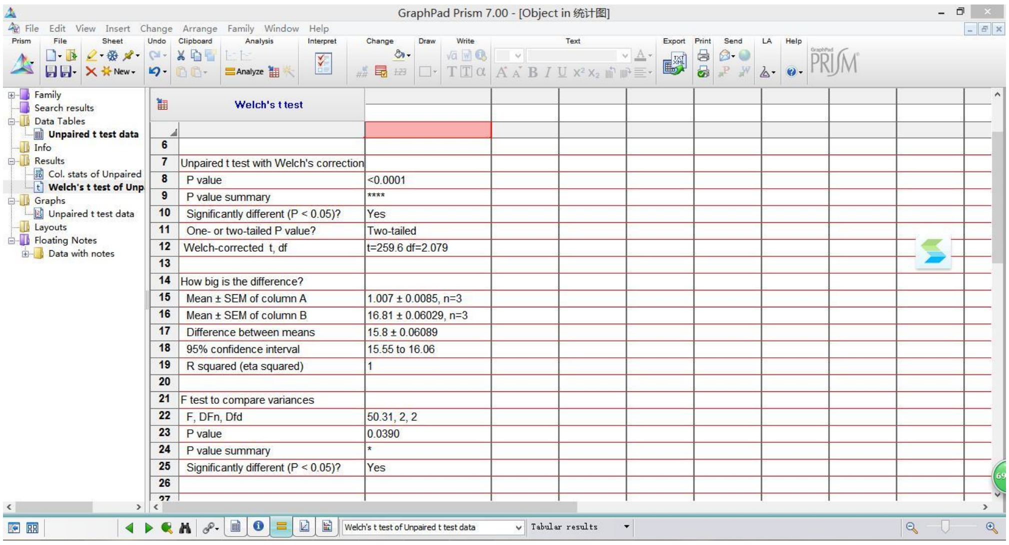

GraphPad Prism 7.00 - [Object in 统计图]
Welch's t test
7 Unpaired t test with Welch's correction
8 P value <0.0001
9 P value summary ****
10 Significantly different (P < 0.05)? Yes
11 One- or two-tailed P value? Two-tailed
12 Welch-corrected t, df t=259.6 df=2.079
14 How big is the difference?
15 Mean ± SEM of column A 1.007 ± 0.0085, n=3
16 Mean ± SEM of column B 16.81 ± 0.06029, n=3
17 Difference between means 15.8 ± 0.06089
18 95% confidence interval 15.55 to 16.06
19 R squared (eta squared) 1
21 F test to compare variances
22 F, DFn, Dfd 50.31, 2, 2
23 P value 0.0390
24 P value summary *
25 Significantly different (P < 0.05)? Yes
Welch's t test of Unpaired t test data
Tabular results

Figure 2E

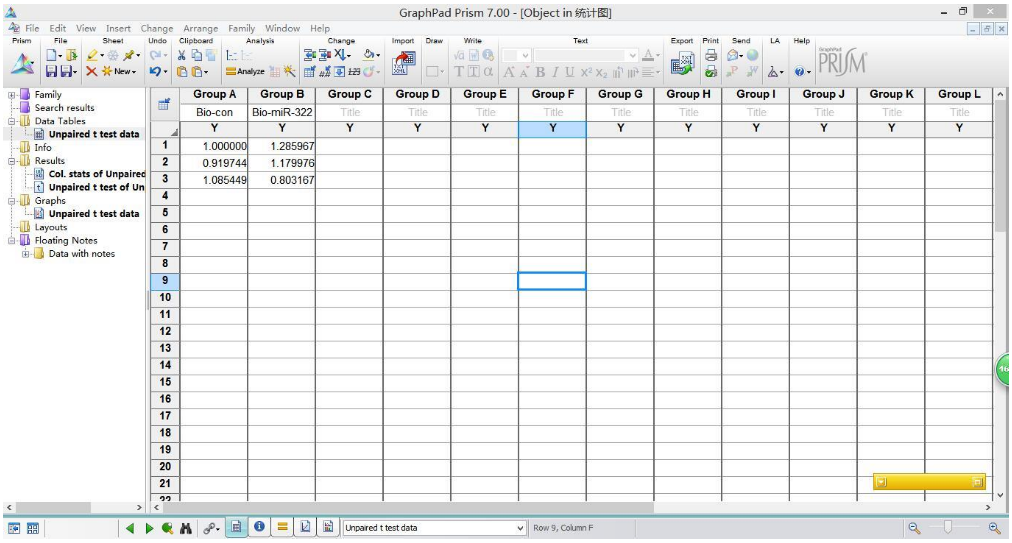

	Group A	Group B
	Bio-con	Bio-miR-322
	Y	Y
1	1.000000	1.285967
2	0.919744	1.179976
3	1.085449	0.803167

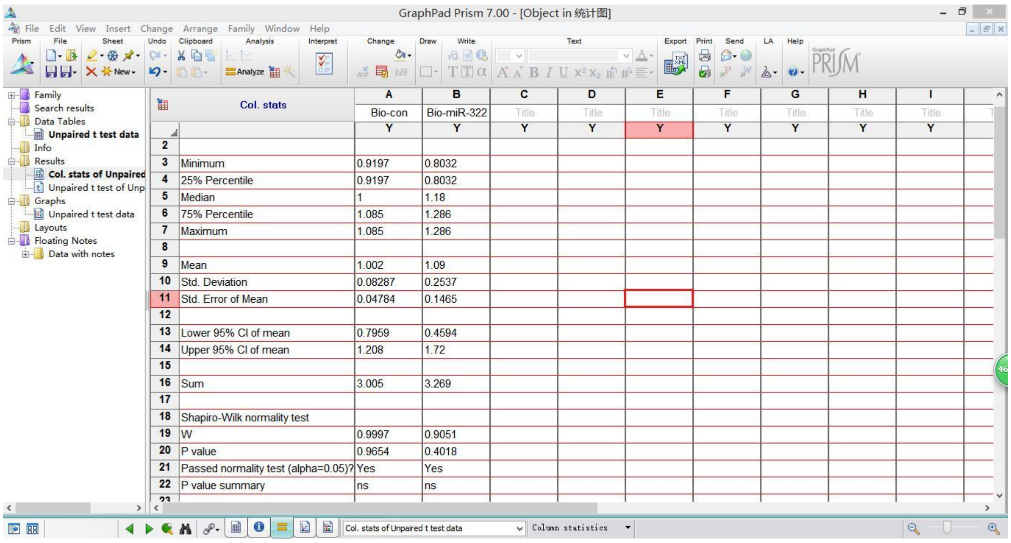

	Col. stats	A	B
		Bio-con	Bio-miR-322
		Y	Y
3	Minimum	0.9197	0.8032
4	25% Percentile	0.9197	0.8032
5	Median	1	1.18
6	75% Percentile	1.085	1.286
7	Maximum	1.085	1.286
9	Mean	1.002	1.09
10	Std. Deviation	0.08287	0.2537
11	Std. Error of Mean	0.04784	0.1465
13	Lower 95% CI of mean	0.7959	0.4594
14	Upper 95% CI of mean	1.208	1.72
16	Sum	3.005	3.269
18	Shapiro-Wilk normality test		
19	W	0.9997	0.9051
20	P value	0.9654	0.4018
21	Passed normality test (alpha=0.05)?	Yes	Yes
22	P value summary	ns	ns

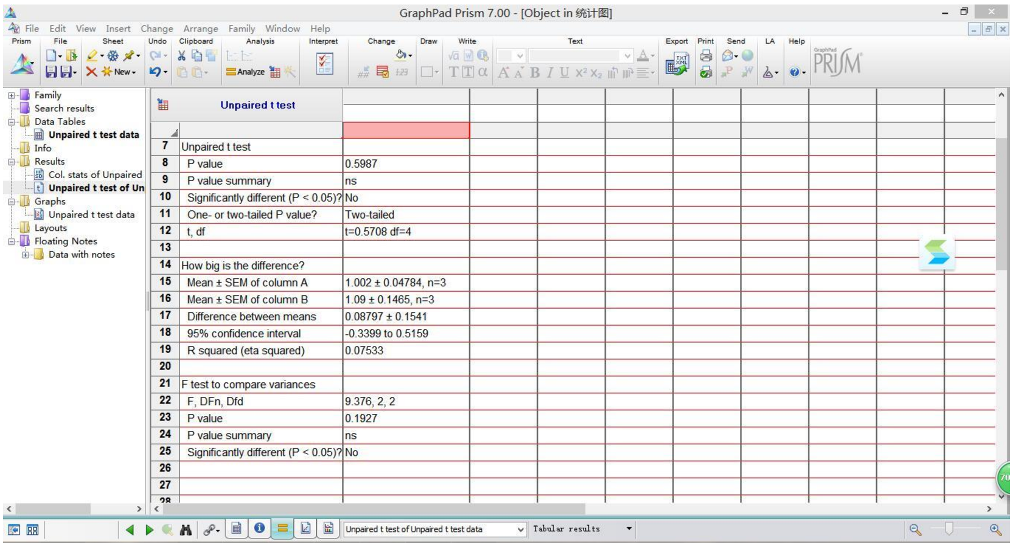

	Unpaired t test	
7	Unpaired t test	
8	P value	0.5987
9	P value summary	ns
10	Significantly different (P < 0.05)?	No
11	One- or two-tailed P value?	Two-tailed
12	t, df	t=0.5708 df=4
14	How big is the difference?	
15	Mean ± SEM of column A	1.002 ± 0.04784, n=3
16	Mean ± SEM of column B	1.09 ± 0.1465, n=3
17	Difference between means	0.08797 ± 0.1541
18	95% confidence interval	-0.3399 to 0.5159
19	R squared (eta squared)	0.07533
21	F test to compare variances	
22	F, DFn, Dfd	9.376, 2, 2
23	P value	0.1927
24	P value summary	ns
25	Significantly different (P < 0.05)?	No

Figure 3A

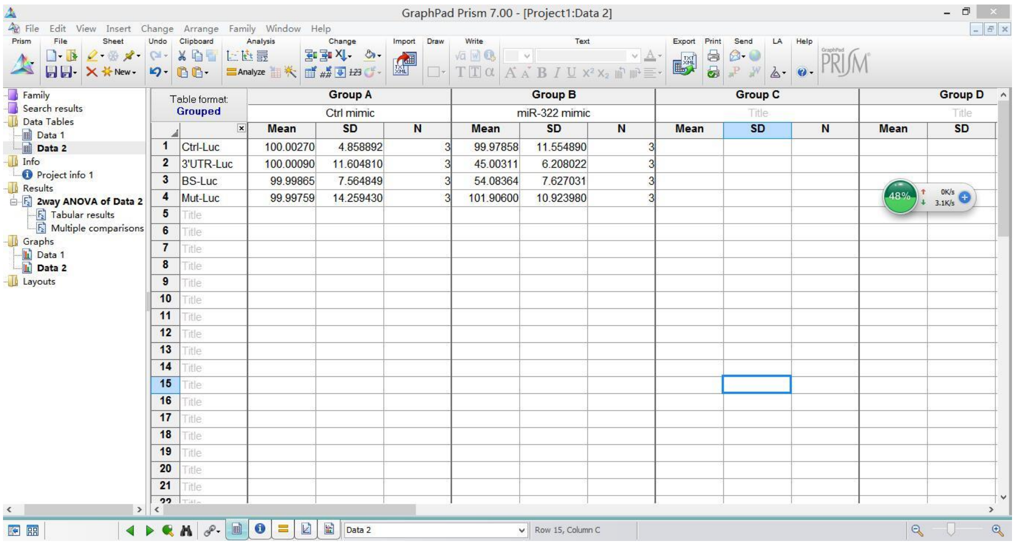
GraphPad Prism 7.00 - [Project1:Data 2]
Table format: Grouped
Group A
Ctrl mimic
Group B
miR-322 mimic
Group C
Group D
Mean
SD
N
1 Ctrl-Luc 100.00270 4.858892 3 99.97858 11.554890 3
2 3'UTR-Luc 100.00090 11.604810 3 45.00311 6.208022 3
3 BS-Luc 99.99865 7.564849 3 54.08364 7.627031 3
4 Mut-Luc 99.99759 14.259430 3 101.90600 10.923980 3
Data 2
Row 15, Column C

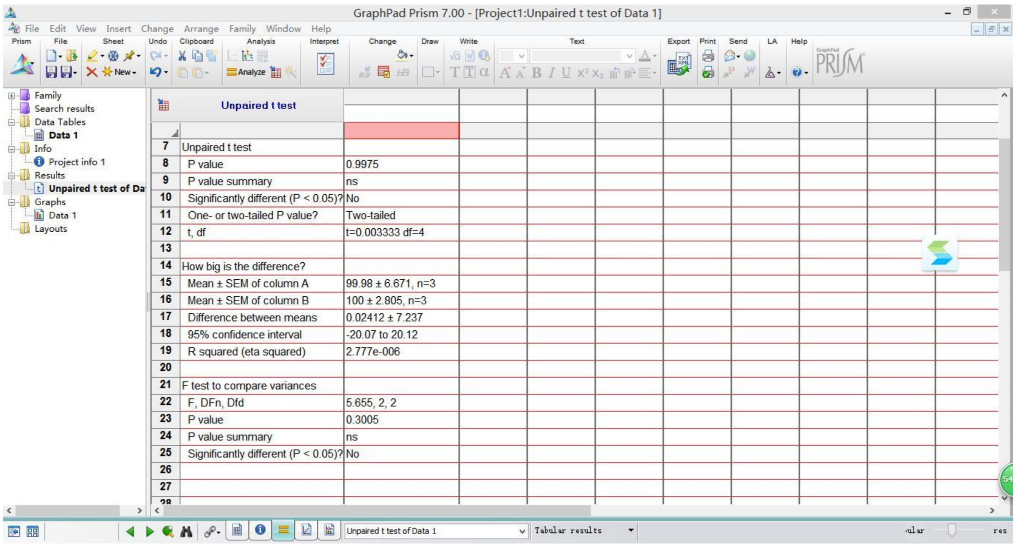
GraphPad Prism 7.00 - [Project1:Unpaired t test of Data 1]
Unpaired t test
7 Unpaired t test
8 P value 0.9975
9 P value summary ns
10 Significantly different (P < 0.05)? No
11 One- or two-tailed P value? Two-tailed
12 t, df t=0.003333 df=4
14 How big is the difference?
15 Mean ± SEM of column A 99.98 ± 6.671, n=3
16 Mean ± SEM of column B 100 ± 2.805, n=3
17 Difference between means 0.02412 ± 7.237
18 95% confidence interval -20.07 to 20.12
19 R squared (eta squared) 2.777e-006
21 F test to compare variances
22 F, DFn, Dfd 5.655, 2, 2
23 P value 0.3005
24 P value summary ns
25 Significantly different (P < 0.05)? No
Unpaired t test of Data 1
Tabular results

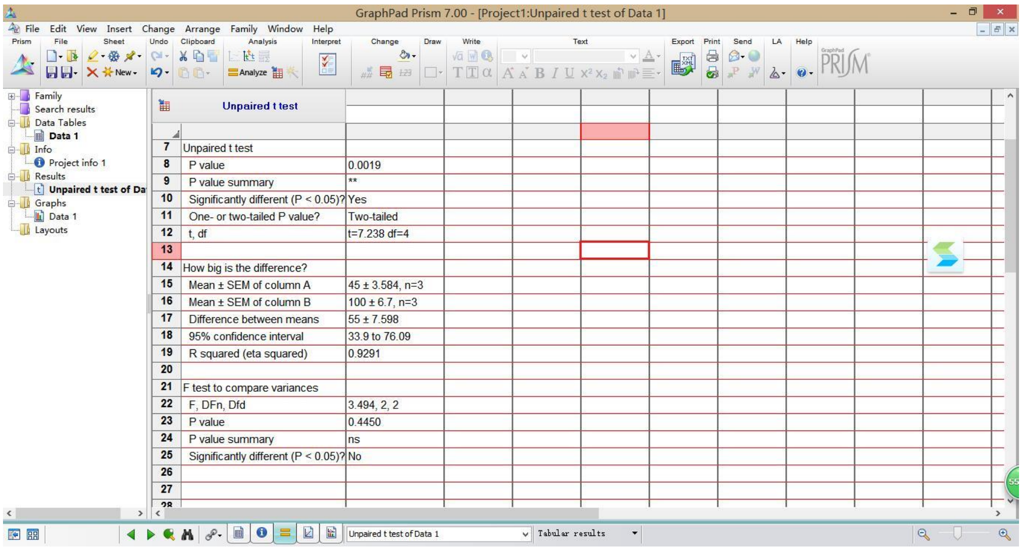
GraphPad Prism 7.00 - [Project1:Unpaired t test of Data 1]
Unpaired t test
7 Unpaired t test
8 P value 0.0019
9 P value summary **
10 Significantly different (P < 0.05)? Yes
11 One- or two-tailed P value? Two-tailed
12 t, df t=7.238 df=4
14 How big is the difference?
15 Mean ± SEM of column A 45 ± 3.584, n=3
16 Mean ± SEM of column B 100 ± 6.7, n=3
17 Difference between means 55 ± 7.598
18 95% confidence interval 33.9 to 76.09
19 R squared (eta squared) 0.9291
21 F test to compare variances
22 F, DFn, Dfd 3.494, 2, 2
23 P value 0.4450
24 P value summary ns
25 Significantly different (P < 0.05)? No
Unpaired t test of Data 1
Tabular results

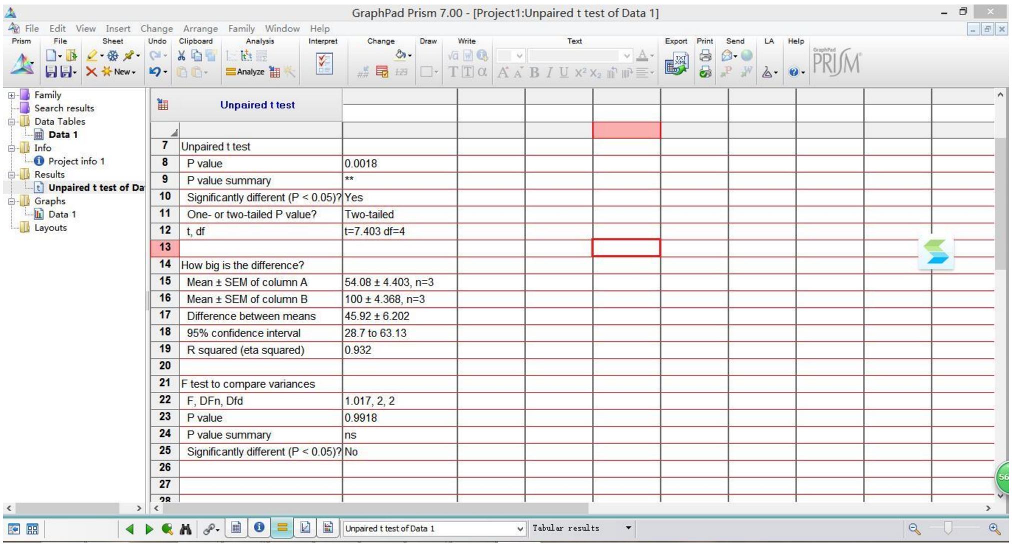

GraphPad Prism 7.00 - [Project1:Unpaired t test of Data 1]

	Unpaired t test	
7	Unpaired t test	
8	P value	0.0018
9	P value summary	**
10	Significantly different (P < 0.05)?	Yes
11	One- or two-tailed P value?	Two-tailed
12	t, df	t=7.403 df=4
13		
14	How big is the difference?	
15	Mean ± SEM of column A	54.08 ± 4.403, n=3
16	Mean ± SEM of column B	100 ± 4.368, n=3
17	Difference between means	45.92 ± 6.202
18	95% confidence interval	28.7 to 63.13
19	R squared (eta squared)	0.932
20		
21	F test to compare variances	
22	F, DFn, Dfd	1.017, 2, 2
23	P value	0.9918
24	P value summary	ns
25	Significantly different (P < 0.05)?	No

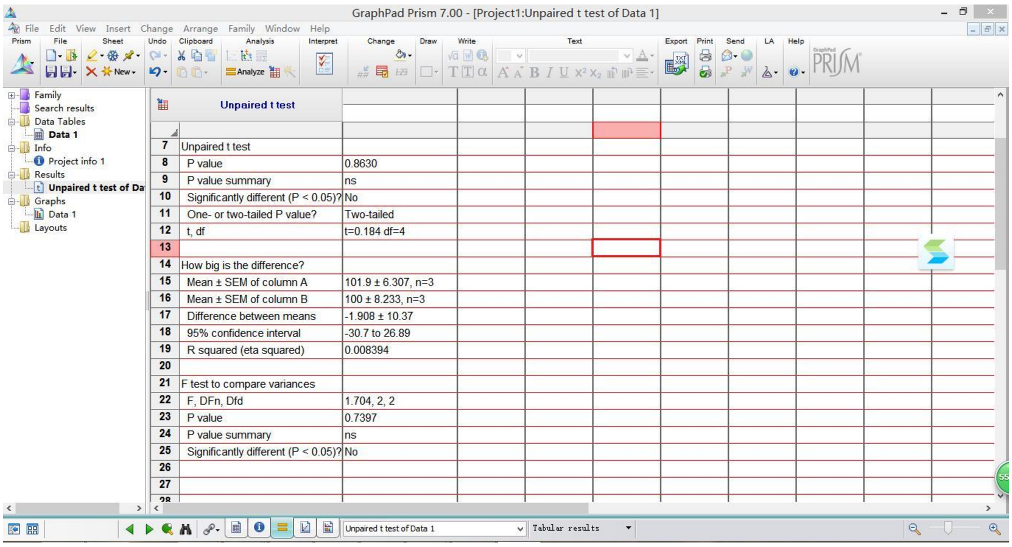

GraphPad Prism 7.00 - [Project1:Unpaired t test of Data 1]

	Unpaired t test	
7	Unpaired t test	
8	P value	0.8630
9	P value summary	ns
10	Significantly different (P < 0.05)?	No
11	One- or two-tailed P value?	Two-tailed
12	t, df	t=0.184 df=4
13		
14	How big is the difference?	
15	Mean ± SEM of column A	101.9 ± 6.307, n=3
16	Mean ± SEM of column B	100 ± 8.233, n=3
17	Difference between means	-1.908 ± 10.37
18	95% confidence interval	-30.7 to 26.89
19	R squared (eta squared)	0.008394
20		
21	F test to compare variances	
22	F, DFn, Dfd	1.704, 2, 2
23	P value	0.7397
24	P value summary	ns
25	Significantly different (P < 0.05)?	No

Figure 3B

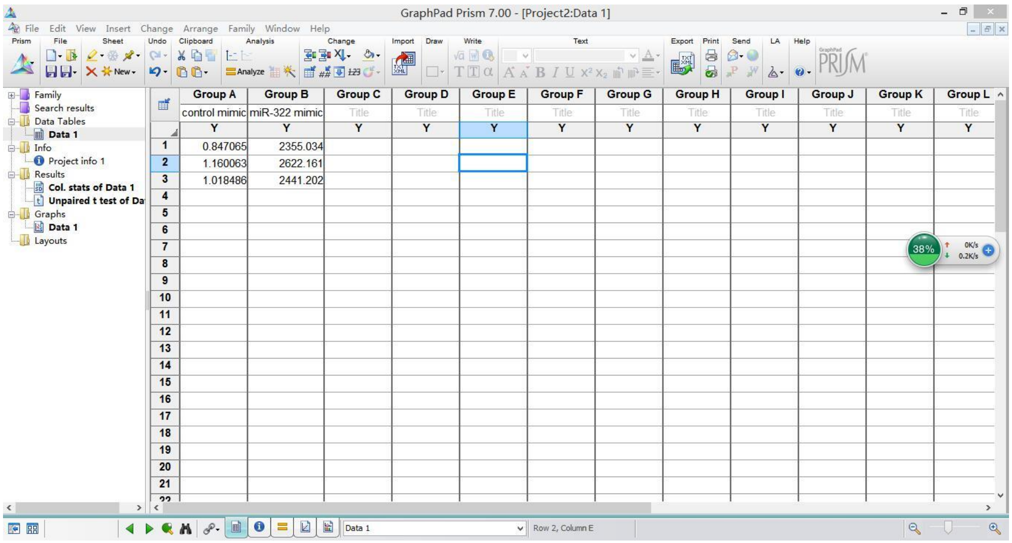
GraphPad Prism 7.00 - [Project2:Data 1]
Group A
Group B
control mimic
miR-322 mimic
0.847065
2355.034
1.160063
2622.161
1.018486
2441.202
Row 2, Column E

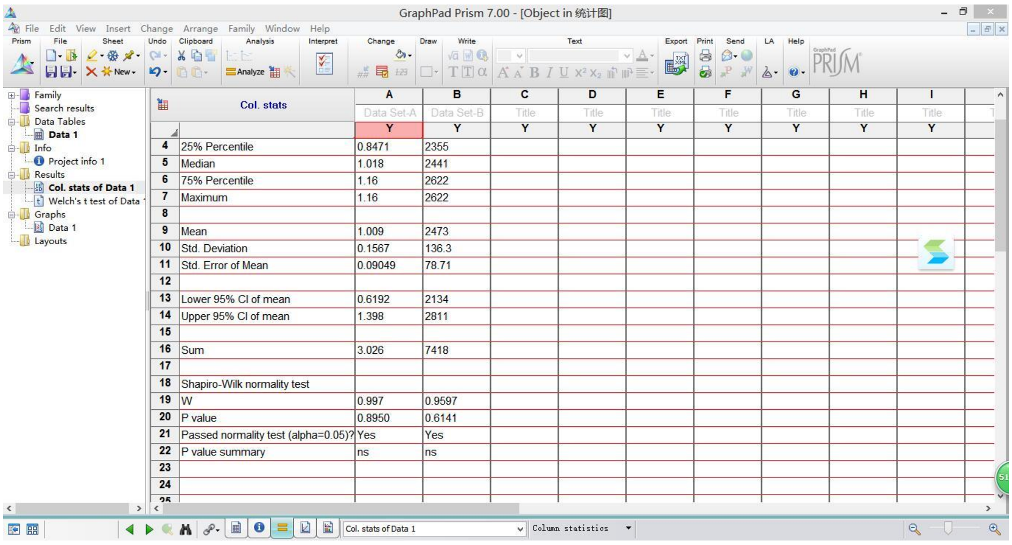
GraphPad Prism 7.00 - [Object in 统计图]
Col. stats
Data Set-A
Data Set-B
25% Percentile 0.8471 2355
Median 1.018 2441
75% Percentile 1.16 2622
Maximum 1.16 2622
Mean 1.009 2473
Std. Deviation 0.1567 136.3
Std. Error of Mean 0.09049 78.71
Lower 95% CI of mean 0.6192 2134
Upper 95% CI of mean 1.398 2811
Sum 3.026 7418
Shapiro-Wilk normality test
W 0.997 0.9597
P value 0.8950 0.6141
Passed normality test (alpha=0.05)? Yes Yes
P value summary ns ns
Col. stats of Data 1
Column statistics

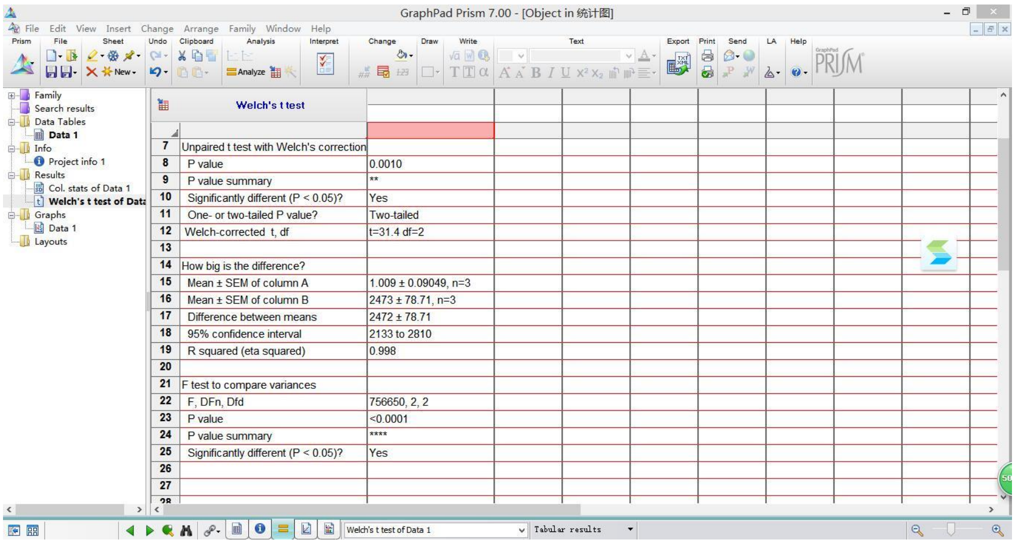
GraphPad Prism 7.00 - [Object in 统计图]
Welch's t test
Unpaired t test with Welch's correction
P value 0.0010
P value summary **
Significantly different (P < 0.05)? Yes
One- or two-tailed P value? Two-tailed
Welch-corrected t, df t=31.4 df=2
How big is the difference?
Mean ± SEM of column A 1.009 ± 0.09049, n=3
Mean ± SEM of column B 2473 ± 78.71, n=3
Difference between means 2472 ± 78.71
95% confidence interval 2133 to 2810
R squared (eta squared) 0.998
F test to compare variances
F, DFn, Dfd 756650, 2, 2
P value <0.0001
P value summary ****
Significantly different (P < 0.05)? Yes
Welch's t test of Data 1
Tabular results

Figure 3C

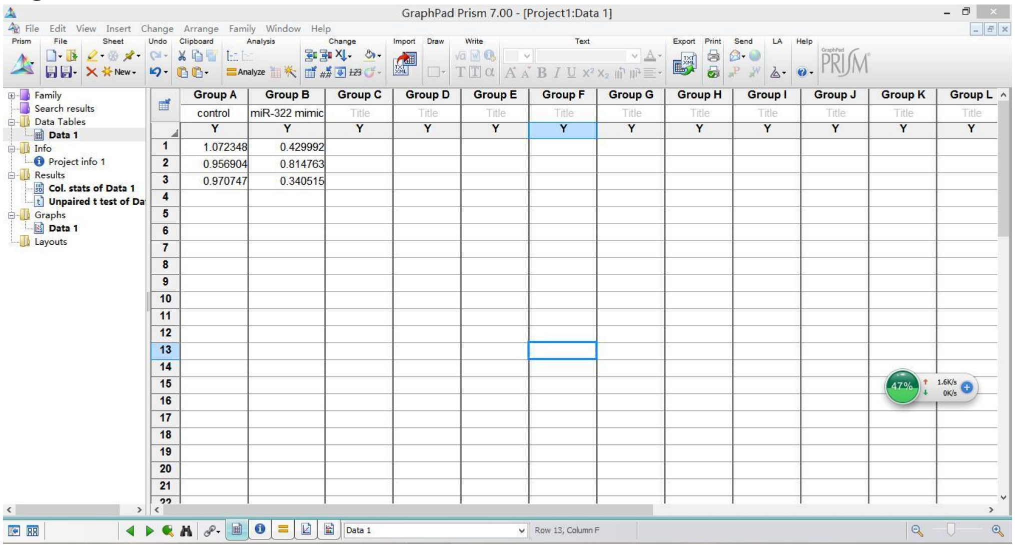

	Group A	Group B
	control	miR-322 mimic
	Y	Y
1	1.072348	0.429992
2	0.956904	0.814763
3	0.970747	0.340515

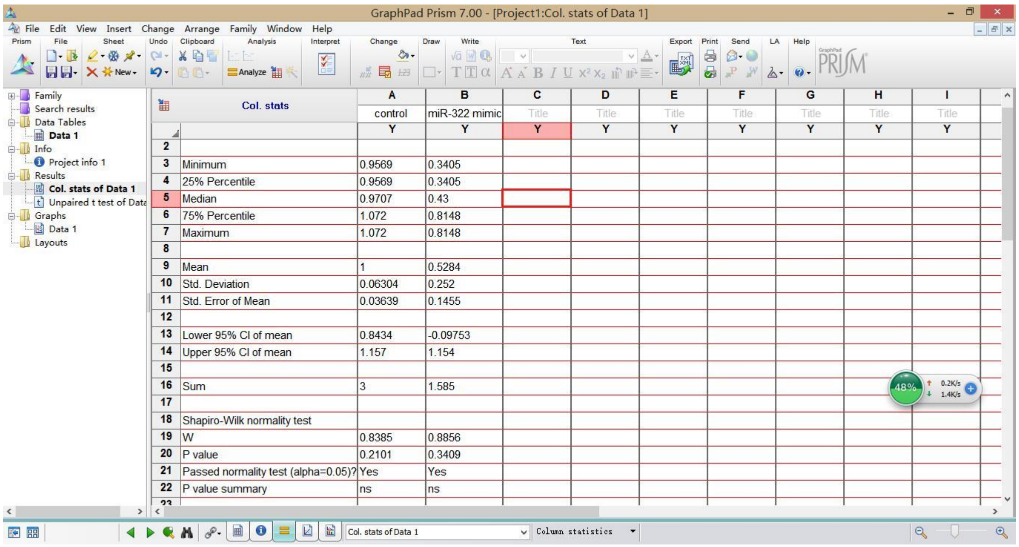

	Col. stats	A	B
		control	miR-322 mimic
		Y	Y
3	Minimum	0.9569	0.3405
4	25% Percentile	0.9569	0.3405
5	Median	0.9707	0.43
6	75% Percentile	1.072	0.8148
7	Maximum	1.072	0.8148
9	Mean	1	0.5284
10	Std. Deviation	0.06304	0.252
11	Std. Error of Mean	0.03639	0.1455
13	Lower 95% CI of mean	0.8434	-0.09753
14	Upper 95% CI of mean	1.157	1.154
16	Sum	3	1.585
18	Shapiro-Wilk normality test		
19	W	0.8385	0.8856
20	P value	0.2101	0.3409
21	Passed normality test (alpha=0.05)?	Yes	Yes
22	P value summary	ns	ns

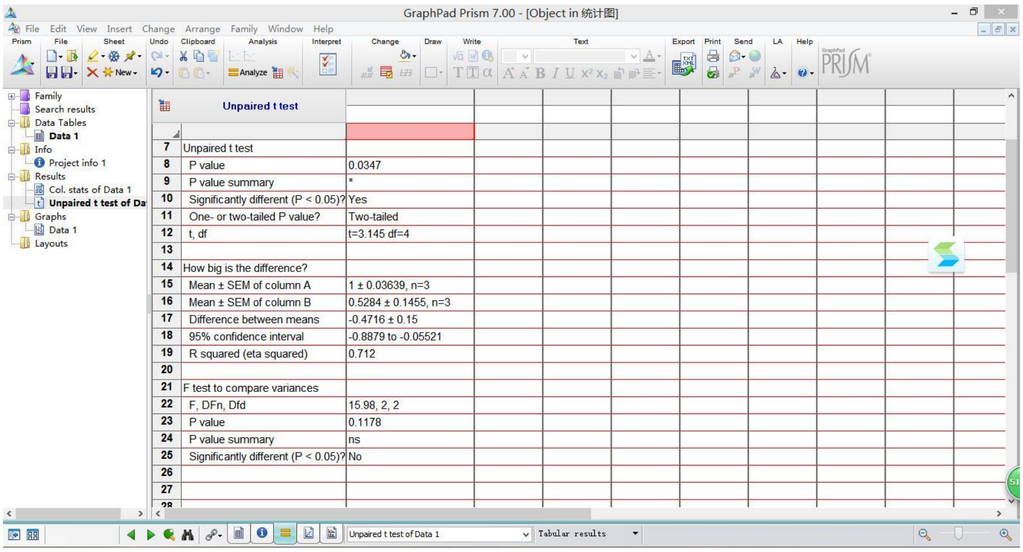

	Unpaired t test	
7	Unpaired t test	
8	P value	0.0347
9	P value summary	*
10	Significantly different (P < 0.05)?	Yes
11	One- or two-tailed P value?	Two-tailed
12	t, df	t=3.145 df=4
14	How big is the difference?	
15	Mean ± SEM of column A	1 ± 0.03639, n=3
16	Mean ± SEM of column B	0.5284 ± 0.1455, n=3
17	Difference between means	-0.4716 ± 0.15
18	95% confidence interval	-0.8879 to -0.05521
19	R squared (eta squared)	0.712
21	F test to compare variances	
22	F, DFn, Dfd	15.98, 2, 2
23	P value	0.1178
24	P value summary	ns
25	Significantly different (P < 0.05)?	No

Figure 3D

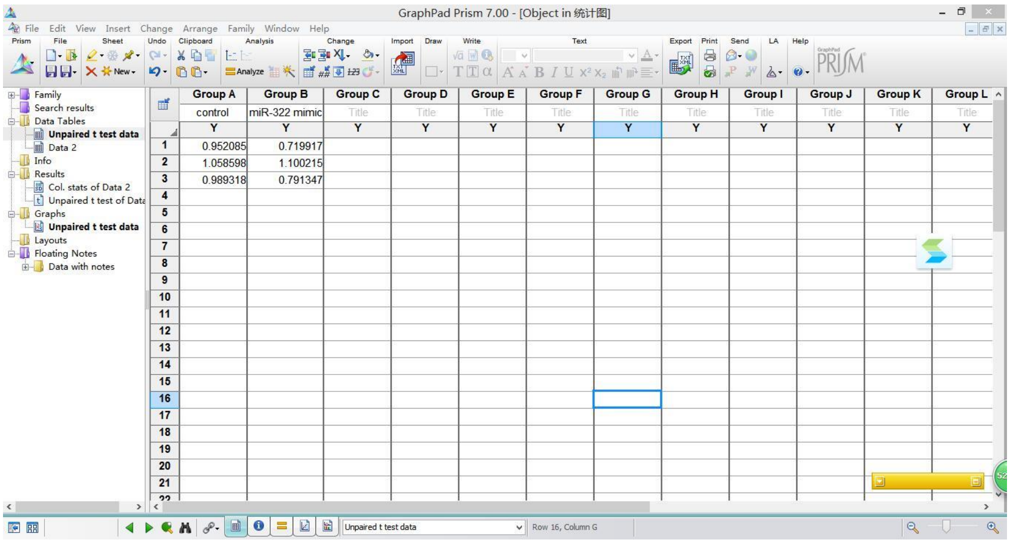
GraphPad Prism 7.00 - [Object in 统计图]
Group A control
Group B miR-322 mimic
1 0.952085 0.719917
2 1.058598 1.100215
3 0.989318 0.791347
Unpaired t test data
Row 16, Column G

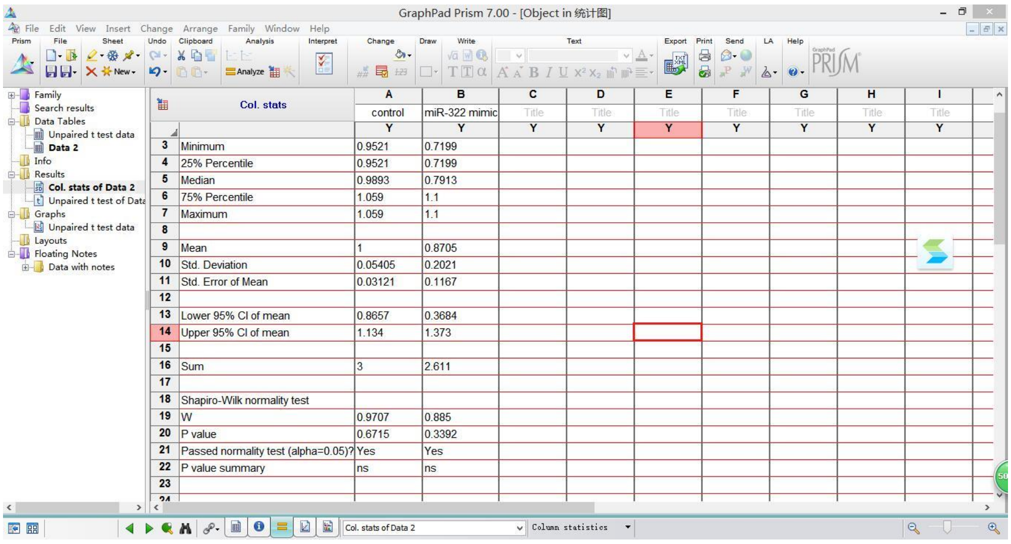
GraphPad Prism 7.00 - [Object in 统计图]
Col. stats
A control
B miR-322 mimic
3 Minimum 0.9521 0.7199
4 25% Percentile 0.9521 0.7199
5 Median 0.9893 0.7913
6 75% Percentile 1.059 1.1
7 Maximum 1.059 1.1
9 Mean 1 0.8705
10 Std. Deviation 0.05405 0.2021
11 Std. Error of Mean 0.03121 0.1167
13 Lower 95% CI of mean 0.8657 0.3684
14 Upper 95% CI of mean 1.134 1.373
16 Sum 3 2.611
18 Shapiro-Wilk normality test
19 W 0.9707 0.885
20 P value 0.6715 0.3392
21 Passed normality test (alpha=0.05)? Yes Yes
22 P value summary ns ns
Col. stats of Data 2
Column statistics

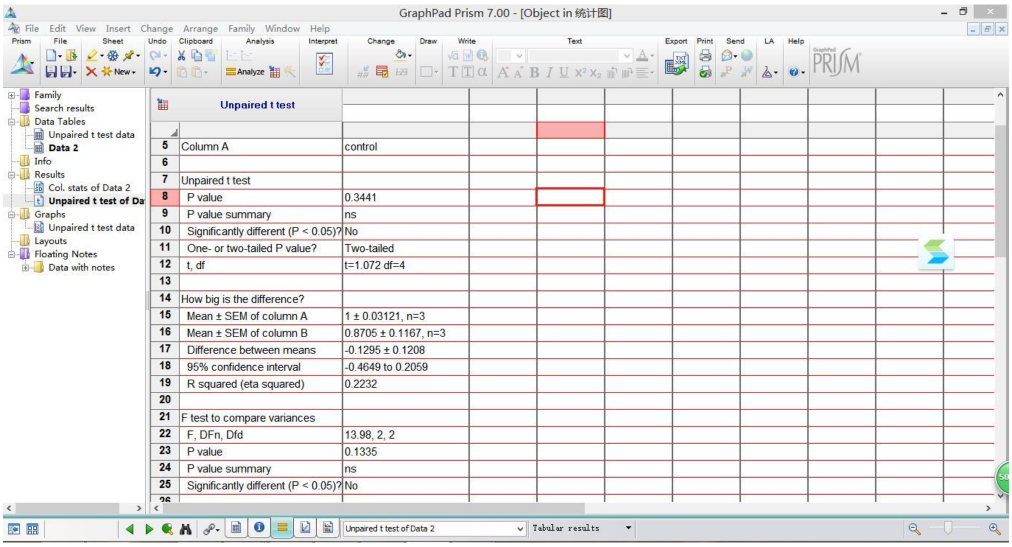
GraphPad Prism 7.00 - [Object in 统计图]
Unpaired t test
5 Column A control
7 Unpaired t test
8 P value 0.3441
9 P value summary ns
10 Significantly different (P < 0.05)? No
11 One- or two-tailed P value? Two-tailed
12 t, df t=1.072 df=4
14 How big is the difference?
15 Mean ± SEM of column A 1 ± 0.03121, n=3
16 Mean ± SEM of column B 0.8705 ± 0.1167, n=3
17 Difference between means -0.1295 ± 0.1208
18 95% confidence interval -0.4649 to 0.2059
19 R squared (eta squared) 0.2232
21 F test to compare variances
22 F, DFn, Dfd 13.98, 2, 2
23 P value 0.1335
24 P value summary ns
25 Significantly different (P < 0.05)? No
Unpaired t test of Data 2
Tabular results

Figure 3E

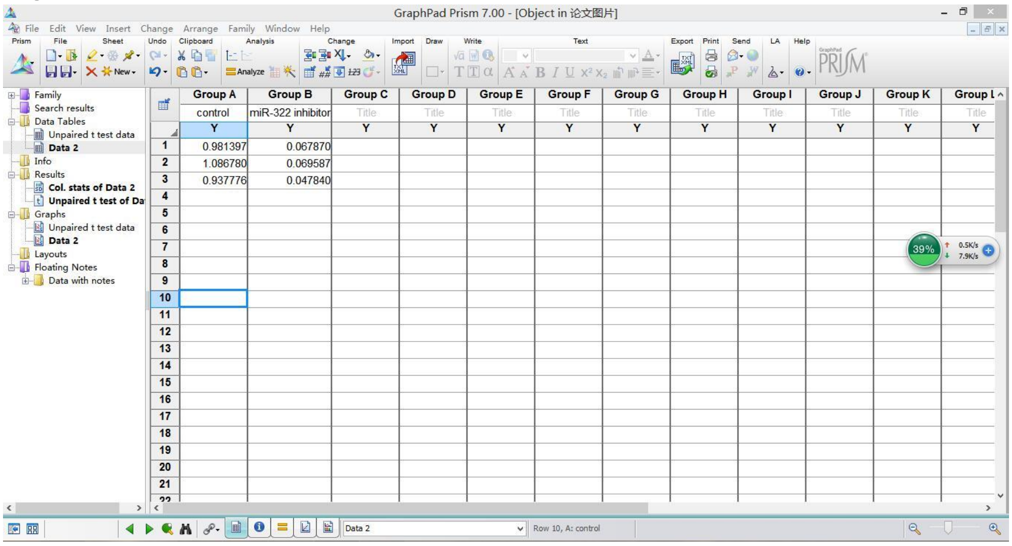

	Group A	Group B
	control	miR-322 inhibitor
	Y	Y
1	0.981397	0.067870
2	1.086780	0.069587
3	0.937776	0.047840

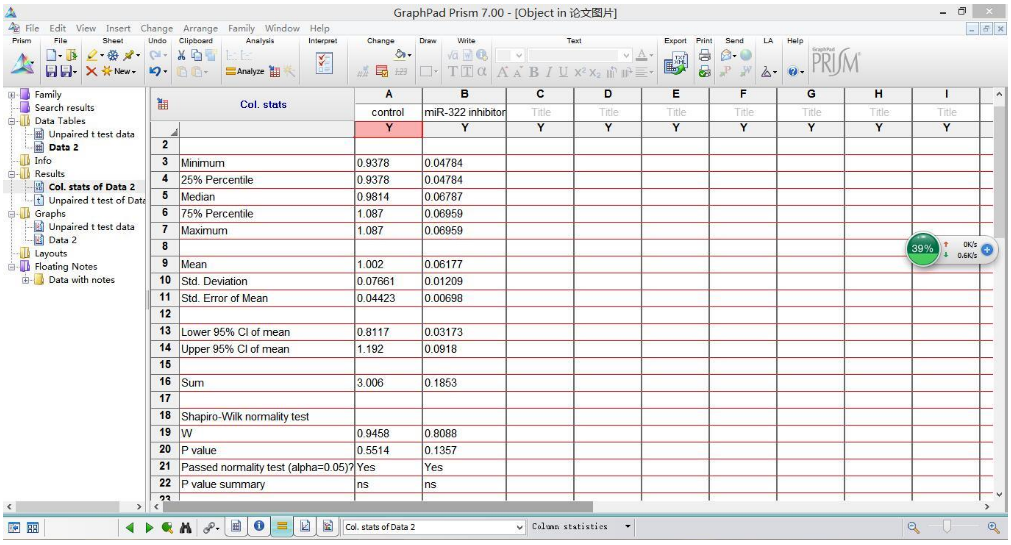

	Col. stats	A	B
		control	miR-322 inhibitor
		Y	Y
3	Minimum	0.9378	0.04784
4	25% Percentile	0.9378	0.04784
5	Median	0.9814	0.06787
6	75% Percentile	1.087	0.06959
7	Maximum	1.087	0.06959
9	Mean	1.002	0.06177
10	Std. Deviation	0.07661	0.01209
11	Std. Error of Mean	0.04423	0.00698
13	Lower 95% CI of mean	0.8117	0.03173
14	Upper 95% CI of mean	1.192	0.0918
16	Sum	3.006	0.1853
18	Shapiro-Wilk normality test		
19	W	0.9458	0.8088
20	P value	0.5514	0.1357
21	Passed normality test (alpha=0.05)?	Yes	Yes
22	P value summary	ns	ns

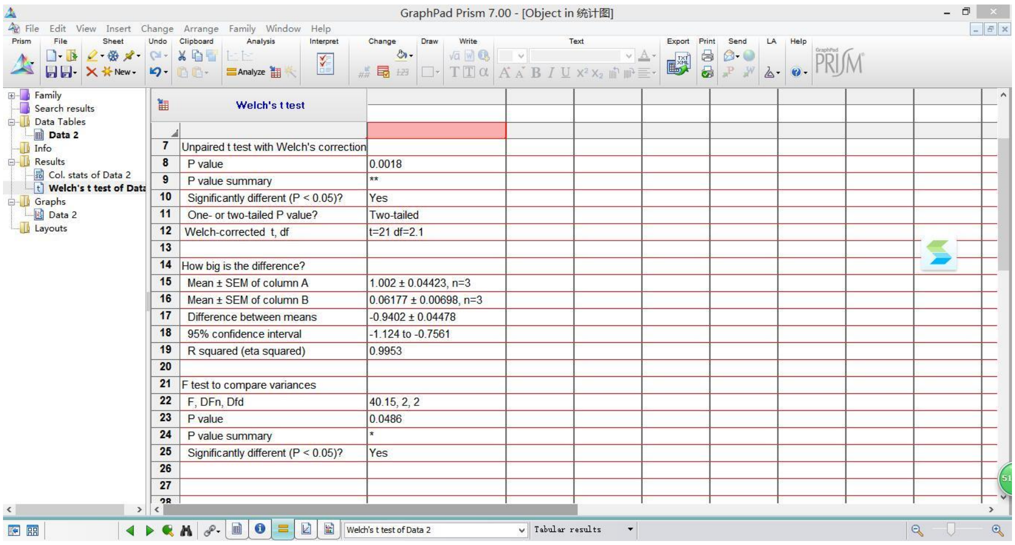

	Welch's t test	
7	Unpaired t test with Welch's correction	
8	P value	0.0018
9	P value summary	**
10	Significantly different (P < 0.05)?	Yes
11	One- or two-tailed P value?	Two-tailed
12	Welch-corrected t, df	t=21 df=2.1
14	How big is the difference?	
15	Mean ± SEM of column A	1.002 ± 0.04423, n=3
16	Mean ± SEM of column B	0.06177 ± 0.00698, n=3
17	Difference between means	-0.9402 ± 0.04478
18	95% confidence interval	-1.124 to -0.7561
19	R squared (eta squared)	0.9953
21	F test to compare variances	
22	F, DFn, Dfd	40.15, 2, 2
23	P value	0.0486
24	P value summary	*
25	Significantly different (P < 0.05)?	Yes

Figure 3F

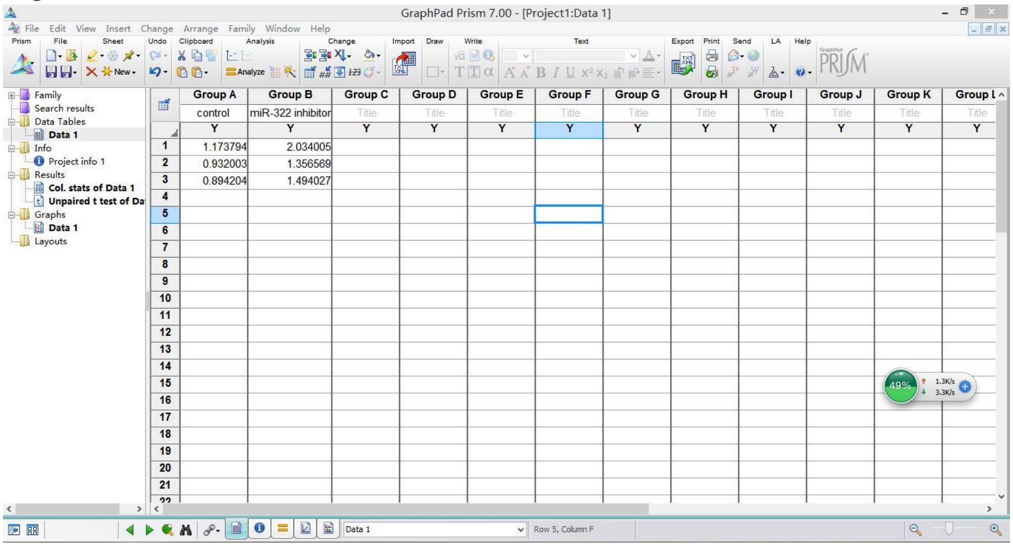

GraphPad Prism 7.00 - [Project1:Data 1]
Group A
Group B
control
miR-322 inhibitor
1.173794
2.034005
0.932003
1.356569
0.894204
1.494027
Row 5, Column F

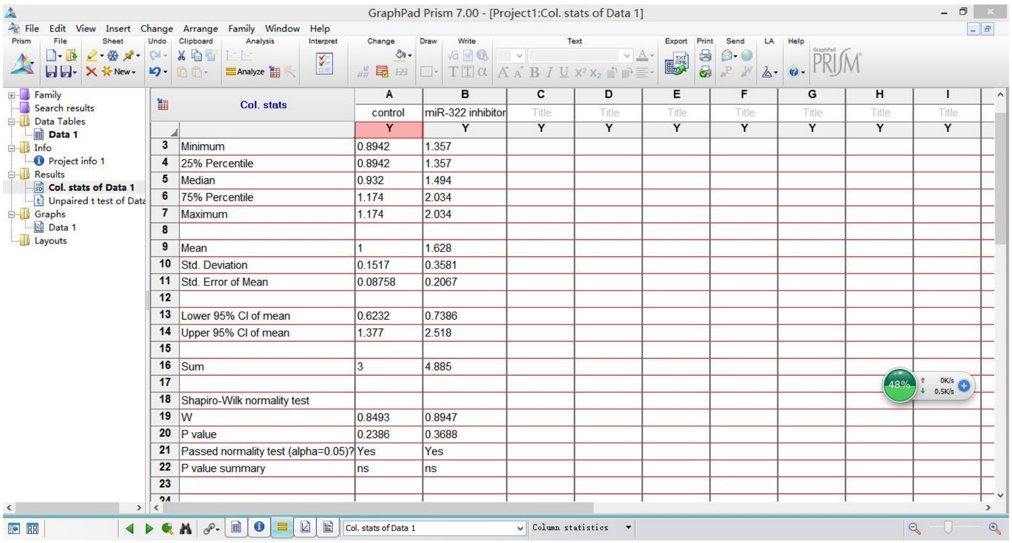

GraphPad Prism 7.00 - [Project1:Col. stats of Data 1]
Col. stats
control
miR-322 inhibitor
Minimum 0.8942 1.357
25% Percentile 0.8942 1.357
Median 0.932 1.494
75% Percentile 1.174 2.034
Maximum 1.174 2.034
Mean 1 1.628
Std. Deviation 0.1517 0.3581
Std. Error of Mean 0.08758 0.2067
Lower 95% CI of mean 0.6232 0.7386
Upper 95% CI of mean 1.377 2.518
Sum 3 4.885
Shapiro-Wilk normality test
W 0.8493 0.8947
P value 0.2386 0.3688
Passed normality test (alpha=0.05)? Yes Yes
P value summary ns ns

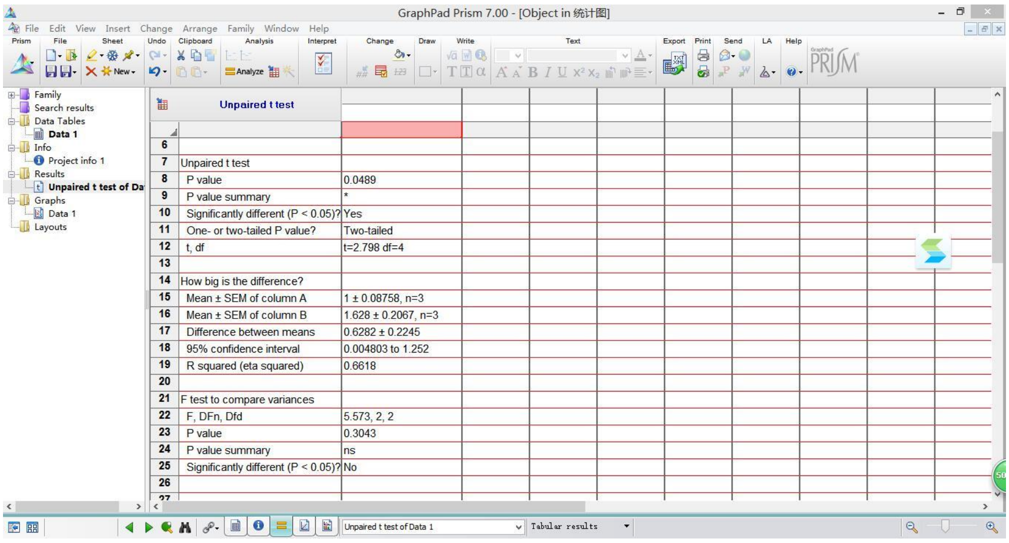

GraphPad Prism 7.00 - [Object in 统计图]
Unpaired t test
Unpaired t test
P value 0.0489
P value summary *
Significantly different (P < 0.05)? Yes
One- or two-tailed P value? Two-tailed
t, df t=2.798 df=4
How big is the difference?
Mean ± SEM of column A 1 ± 0.08758, n=3
Mean ± SEM of column B 1.628 ± 0.2067, n=3
Difference between means 0.6282 ± 0.2245
95% confidence interval 0.004803 to 1.252
R squared (eta squared) 0.6618
F test to compare variances
F, DFn, Dfd 5.573, 2, 2
P value 0.3043
P value summary ns
Significantly different (P < 0.05)? No

Figure 3G

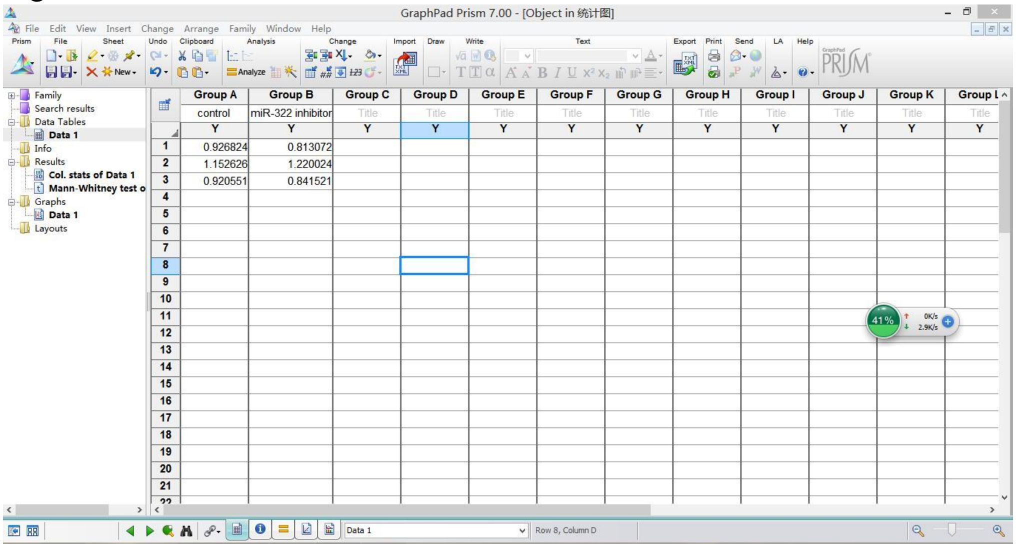

	Group A	Group B
	control	miR-322 inhibitor
1	0.926824	0.813072
2	1.152626	1.220024
3	0.920551	0.841521

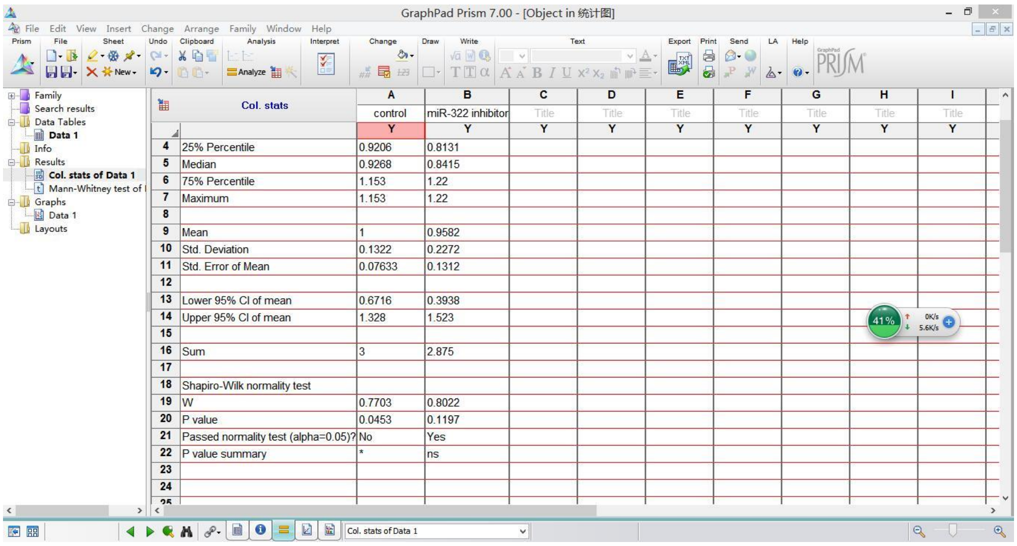

	Col. stats	A control	B miR-322 inhibitor
4	25% Percentile	0.9206	0.8131
5	Median	0.9268	0.8415
6	75% Percentile	1.153	1.22
7	Maximum	1.153	1.22
8			
9	Mean	1	0.9582
10	Std. Deviation	0.1322	0.2272
11	Std. Error of Mean	0.07633	0.1312
12			
13	Lower 95% CI of mean	0.6716	0.3938
14	Upper 95% CI of mean	1.328	1.523
15			
16	Sum	3	2.875
17			
18	Shapiro-Wilk normality test		
19	W	0.7703	0.8022
20	P value	0.0453	0.1197
21	Passed normality test (alpha=0.05)?	No	Yes
22	P value summary	*	ns

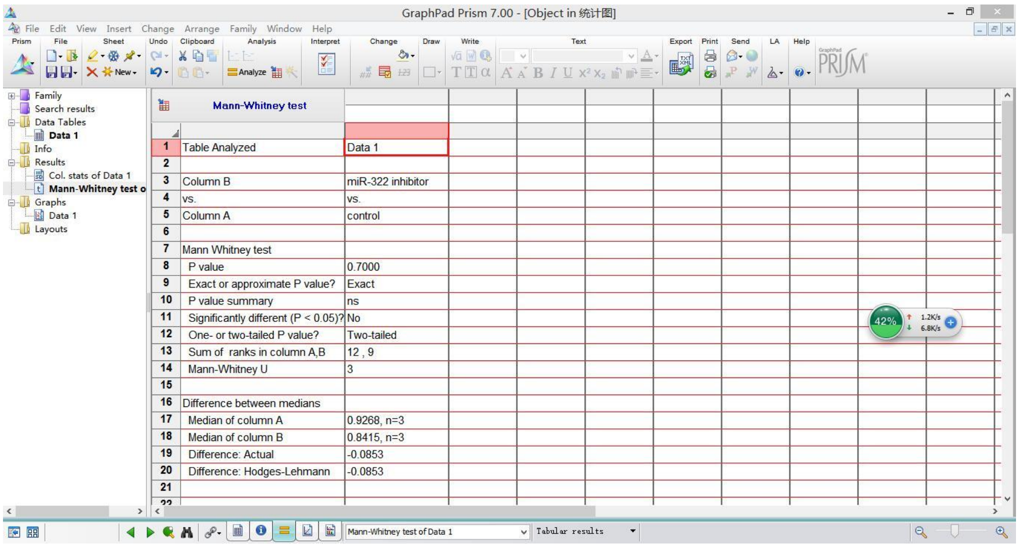

	Mann-Whitney test	
1	Table Analyzed	Data 1
2		
3	Column B	miR-322 inhibitor
4	vs.	vs.
5	Column A	control
6		
7	Mann Whitney test	
8	P value	0.7000
9	Exact or approximate P value?	Exact
10	P value summary	ns
11	Significantly different (P < 0.05)?	No
12	One- or two-tailed P value?	Two-tailed
13	Sum of ranks in column A,B	12 , 9
14	Mann-Whitney U	3
15		
16	Difference between medians	
17	Median of column A	0.9268, n=3
18	Median of column B	0.8415, n=3
19	Difference: Actual	-0.0853
20	Difference: Hodges-Lehmann	-0.0853

Figure 4A (NOX4)

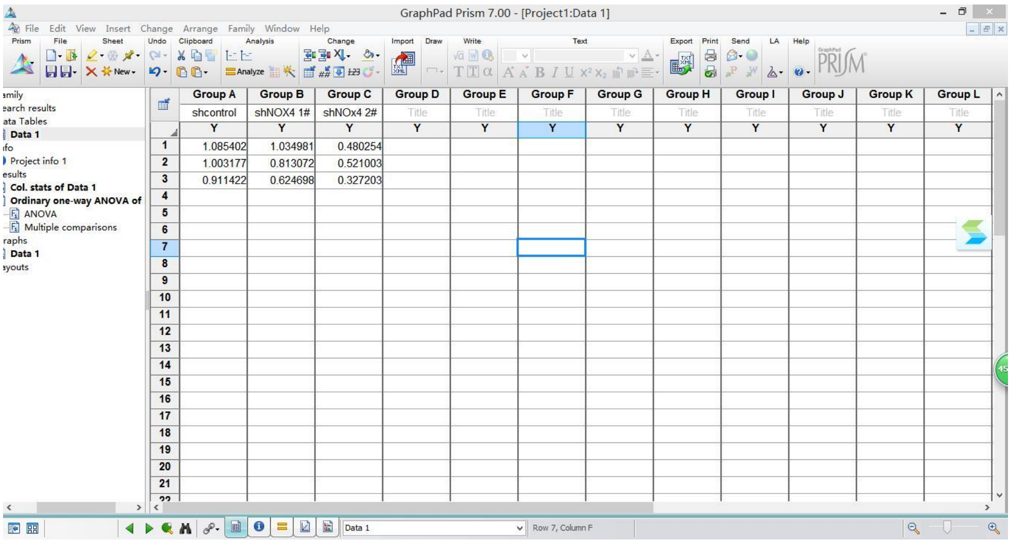

	Group A	Group B	Group C
	shcontrol	shNOX4 1#	shNOx4 2#
	Y	Y	Y
1	1.085402	1.034981	0.480254
2	1.003177	0.813072	0.521003
3	0.911422	0.624698	0.327203

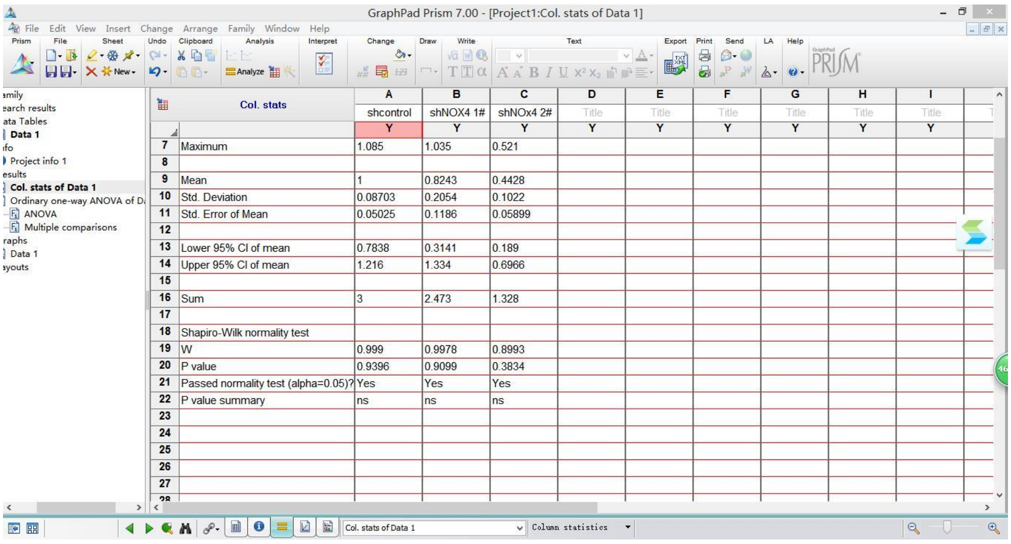

	Col. stats	A	B	C
		shcontrol	shNOX4 1#	shNOx4 2#
7	Maximum	1.085	1.035	0.521
8				
9	Mean	1	0.8243	0.4428
10	Std. Deviation	0.08703	0.2054	0.1022
11	Std. Error of Mean	0.05025	0.1186	0.05899
12				
13	Lower 95% CI of mean	0.7838	0.3141	0.189
14	Upper 95% CI of mean	1.216	1.334	0.6966
15				
16	Sum	3	2.473	1.328
17				
18	Shapiro-Wilk normality test			
19	W	0.999	0.9978	0.8993
20	P value	0.9396	0.9099	0.3834
21	Passed normality test (alpha=0.05)?	Yes	Yes	Yes
22	P value summary	ns	ns	ns

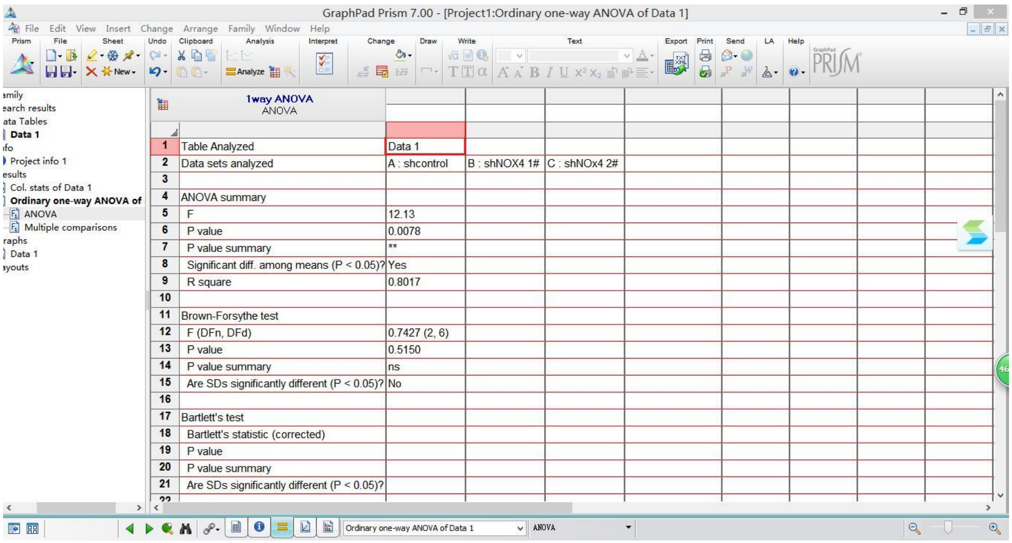

	1way ANOVA ANOVA			
1	Table Analyzed	Data 1		
2	Data sets analyzed	A : shcontrol	B : shNOX4 1#	C : shNOx4 2#
3				
4	ANOVA summary			
5	F	12.13		
6	P value	0.0078		
7	P value summary	**		
8	Significant diff. among means (P < 0.05)?	Yes		
9	R square	0.8017		
10				
11	Brown-Forsythe test			
12	F (DFn, DFd)	0.7427 (2, 6)		
13	P value	0.5150		
14	P value summary	ns		
15	Are SDs significantly different (P < 0.05)?	No		
16				
17	Bartlett's test			
18	Bartlett's statistic (corrected)			
19	P value			
20	P value summary			
21	Are SDs significantly different (P < 0.05)?			

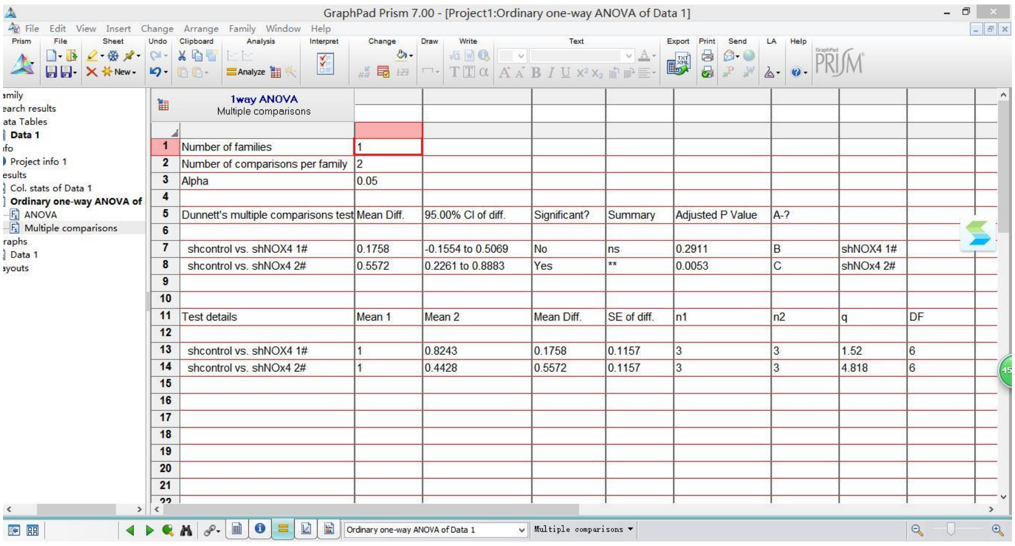

1way ANOVA — Multiple comparisons

1	Number of families	1							
2	Number of comparisons per family	2							
3	Alpha	0.05							
4									
5	Dunnett's multiple comparisons test	Mean Diff.	95.00% CI of diff.	Significant?	Summary	Adjusted P Value	A-?		
6									
7	shcontrol vs. shNOX4 1#	0.1758	-0.1554 to 0.5069	No	ns	0.2911	B	shNOX4 1#	
8	shcontrol vs. shNOx4 2#	0.5572	0.2261 to 0.8883	Yes	**	0.0053	C	shNOx4 2#	
9									
10									
11	Test details	Mean 1	Mean 2	Mean Diff.	SE of diff.	n1	n2	q	DF
12									
13	shcontrol vs. shNOX4 1#	1	0.8243	0.1758	0.1157	3	3	1.52	6
14	shcontrol vs. shNOx4 2#	1	0.4428	0.5572	0.1157	3	3	4.818	6

Figure 4A (Cleaved Caspase-3)

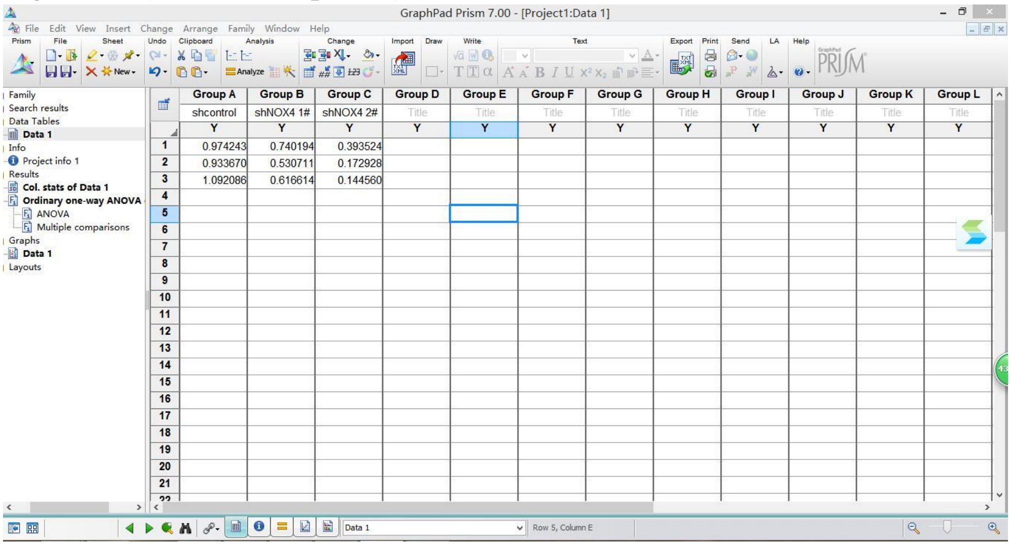

GraphPad Prism 7.00 - [Project1:Data 1]

	Group A	Group B	Group C
	shcontrol	shNOX4 1#	shNOX4 2#
	Y	Y	Y
1	0.974243	0.740194	0.393524
2	0.933670	0.530711	0.172928
3	1.092086	0.616614	0.144560

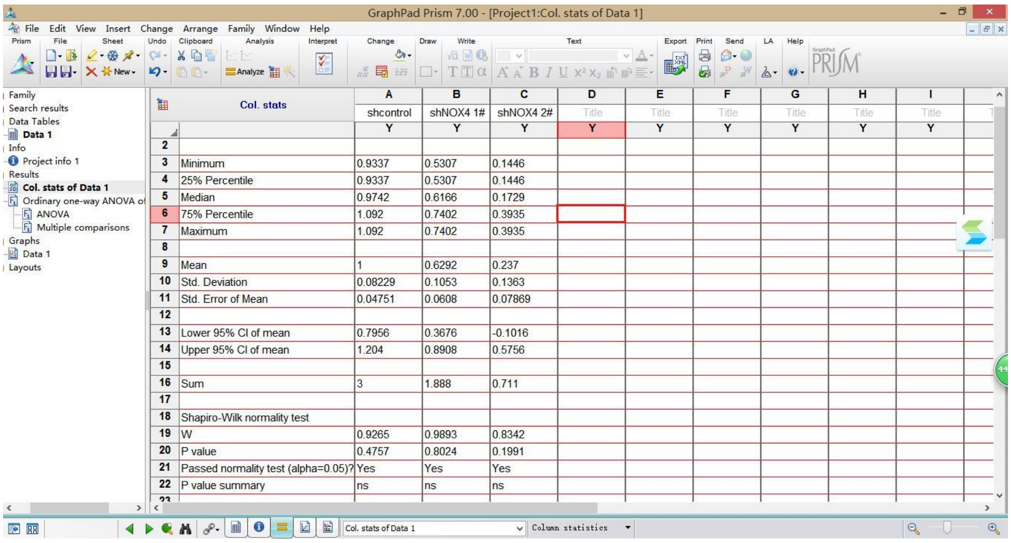

GraphPad Prism 7.00 - [Project1:Col. stats of Data 1]

	Col. stats	A	B	C
		shcontrol	shNOX4 1#	shNOX4 2#
		Y	Y	Y
2				
3	Minimum	0.9337	0.5307	0.1446
4	25% Percentile	0.9337	0.5307	0.1446
5	Median	0.9742	0.6166	0.1729
6	75% Percentile	1.092	0.7402	0.3935
7	Maximum	1.092	0.7402	0.3935
8				
9	Mean	1	0.6292	0.237
10	Std. Deviation	0.08229	0.1053	0.1363
11	Std. Error of Mean	0.04751	0.0608	0.07869
12				
13	Lower 95% CI of mean	0.7956	0.3676	-0.1016
14	Upper 95% CI of mean	1.204	0.8908	0.5756
15				
16	Sum	3	1.888	0.711
17				
18	Shapiro-Wilk normality test			
19	W	0.9265	0.9893	0.8342
20	P value	0.4757	0.8024	0.1991
21	Passed normality test (alpha=0.05)?	Yes	Yes	Yes
22	P value summary	ns	ns	ns

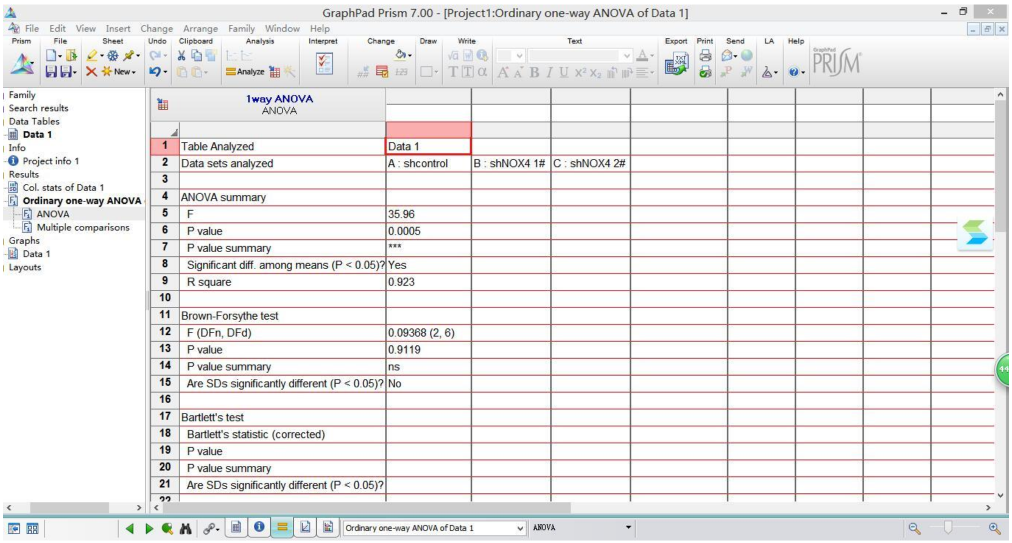

GraphPad Prism 7.00 - [Project1:Ordinary one-way ANOVA of Data 1]

	1way ANOVA<br>ANOVA			
1	Table Analyzed	Data 1		
2	Data sets analyzed	A : shcontrol	B : shNOX4 1#	C : shNOX4 2#
3				
4	ANOVA summary			
5	F	35.96		
6	P value	0.0005		
7	P value summary	***		
8	Significant diff. among means (P < 0.05)?	Yes		
9	R square	0.923		
10				
11	Brown-Forsythe test			
12	F (DFn, DFd)	0.09368 (2, 6)		
13	P value	0.9119		
14	P value summary	ns		
15	Are SDs significantly different (P < 0.05)?	No		
16				
17	Bartlett's test			
18	Bartlett's statistic (corrected)			
19	P value			
20	P value summary			
21	Are SDs significantly different (P < 0.05)?			

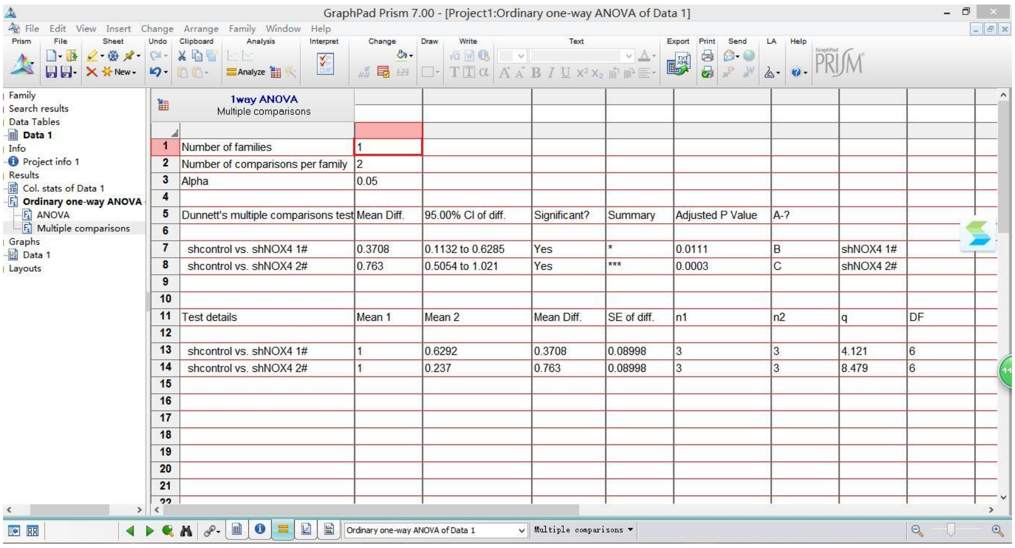

1	Number of families	1							
2	Number of comparisons per family	2							
3	Alpha	0.05							
4									
5	Dunnett's multiple comparisons test	Mean Diff.	95.00% CI of diff.	Significant?	Summary	Adjusted P Value	A-?		
6									
7	shcontrol vs. shNOX4 1#	0.3708	0.1132 to 0.6285	Yes	*	0.0111	B	shNOX4 1#	
8	shcontrol vs. shNOX4 2#	0.763	0.5054 to 1.021	Yes	***	0.0003	C	shNOX4 2#	
9									
10									
11	Test details	Mean 1	Mean 2	Mean Diff.	SE of diff.	n1	n2	q	DF
12									
13	shcontrol vs. shNOX4 1#	1	0.6292	0.3708	0.08998	3	3	4.121	6
14	shcontrol vs. shNOX4 2#	1	0.237	0.763	0.08998	3	3	8.479	6

Figure 4A (Bax/Bcl-2)

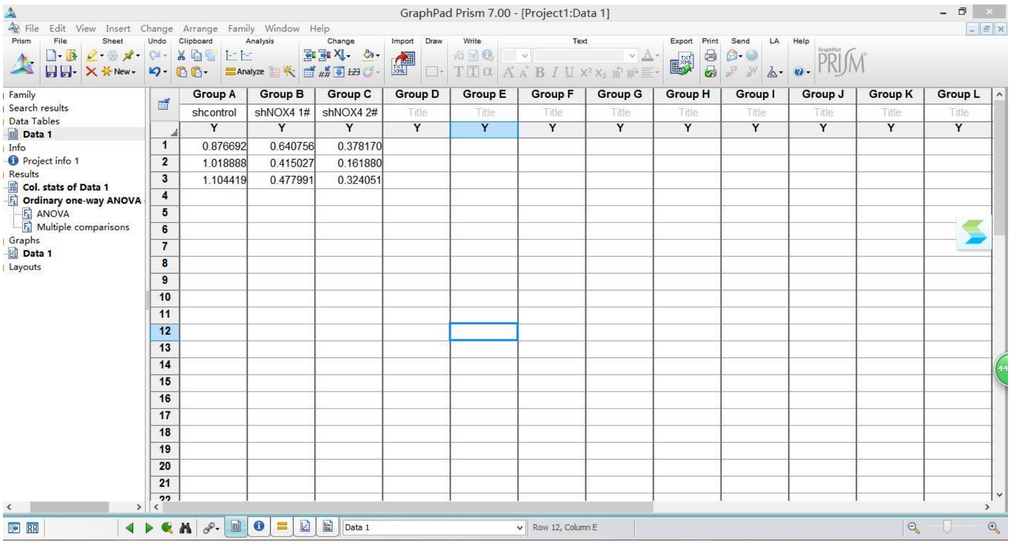

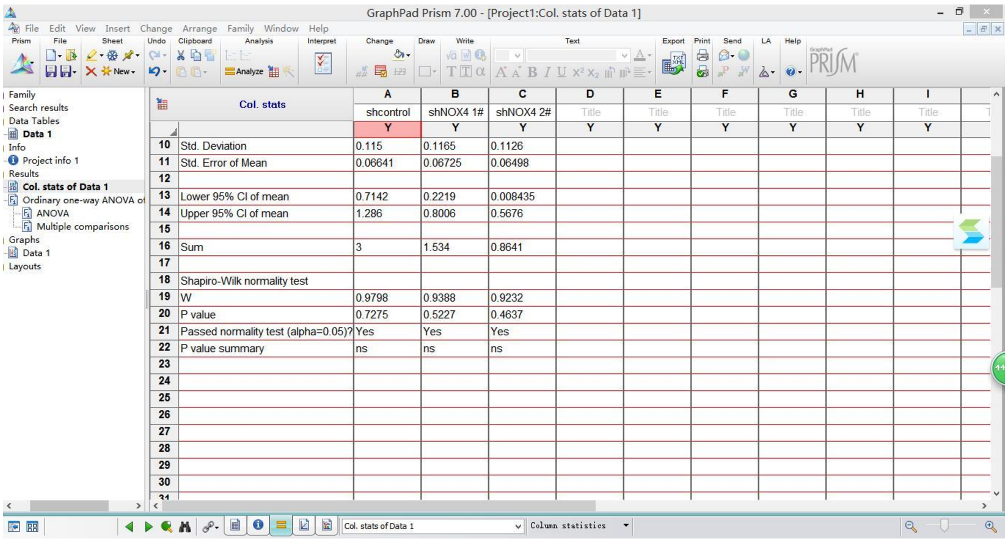

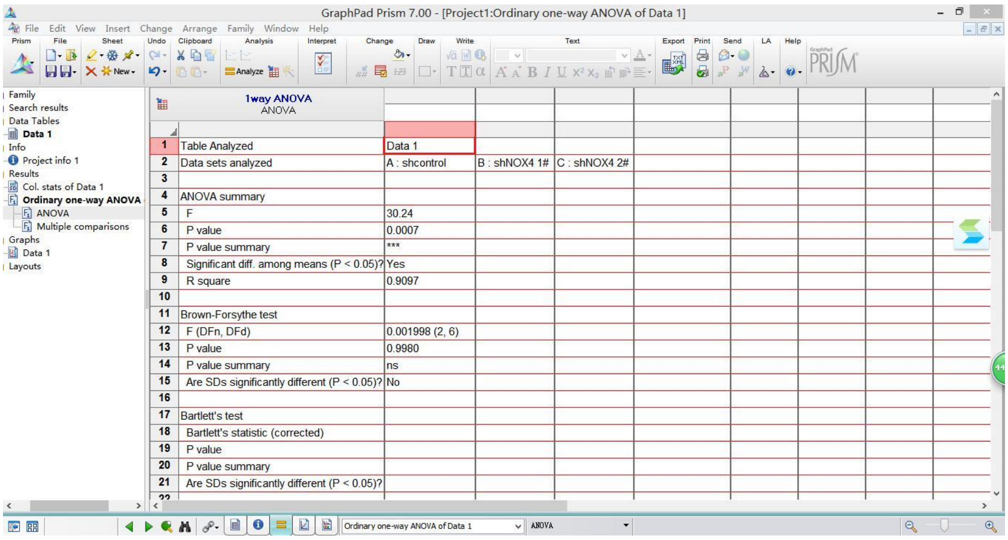

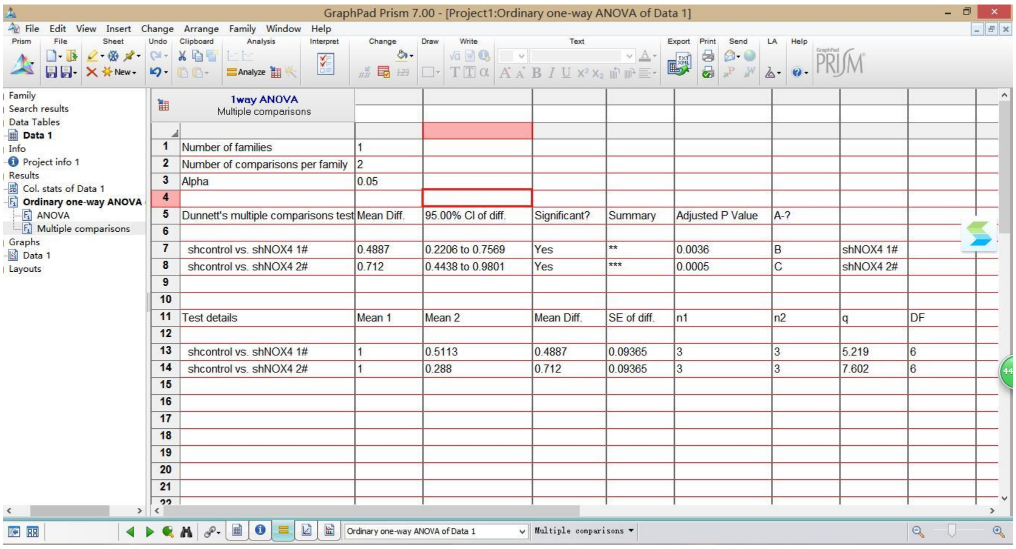

	1way ANOVA Multiple comparisons								
1	Number of families	1							
2	Number of comparisons per family	2							
3	Alpha	0.05							
4									
5	Dunnett's multiple comparisons test	Mean Diff.	95.00% CI of diff.	Significant?	Summary	Adjusted P Value	A-?		
6									
7	shcontrol vs. shNOX4 1#	0.4887	0.2206 to 0.7569	Yes	**	0.0036	B	shNOX4 1#	
8	shcontrol vs. shNOX4 2#	0.712	0.4438 to 0.9801	Yes	***	0.0005	C	shNOX4 2#	
9									
10									
11	Test details	Mean 1	Mean 2	Mean Diff.	SE of diff.	n1	n2	q	DF
12									
13	shcontrol vs. shNOX4 1#	1	0.5113	0.4887	0.09365	3	3	5.219	6
14	shcontrol vs. shNOX4 2#	1	0.288	0.712	0.09365	3	3	7.602	6

Figure 4B (NOX4)

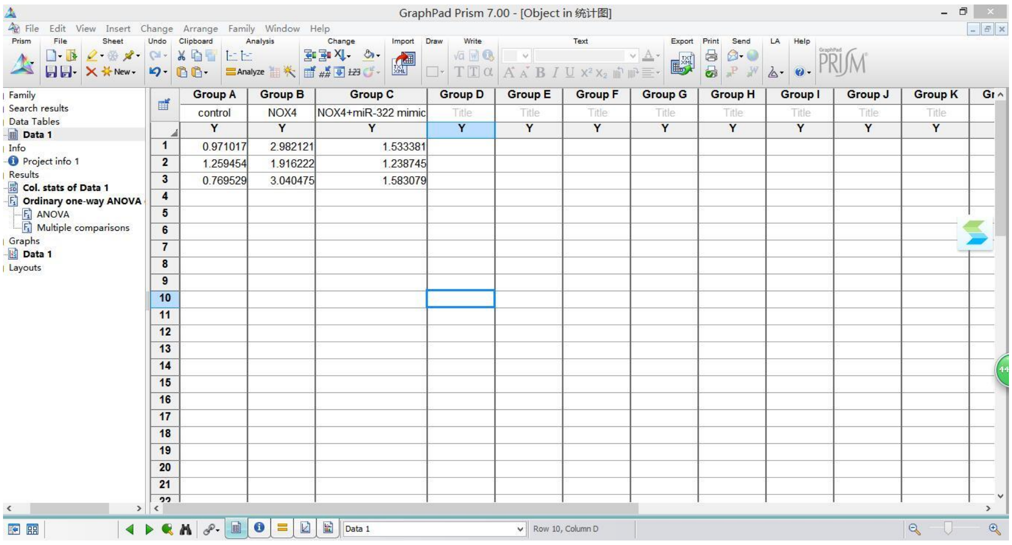

	Group A	Group B	Group C
	control	NOX4	NOX4+miR-322 mimic
	Y	Y	Y
1	0.971017	2.982121	1.533381
2	1.259454	1.916222	1.238745
3	0.769529	3.040475	1.583079

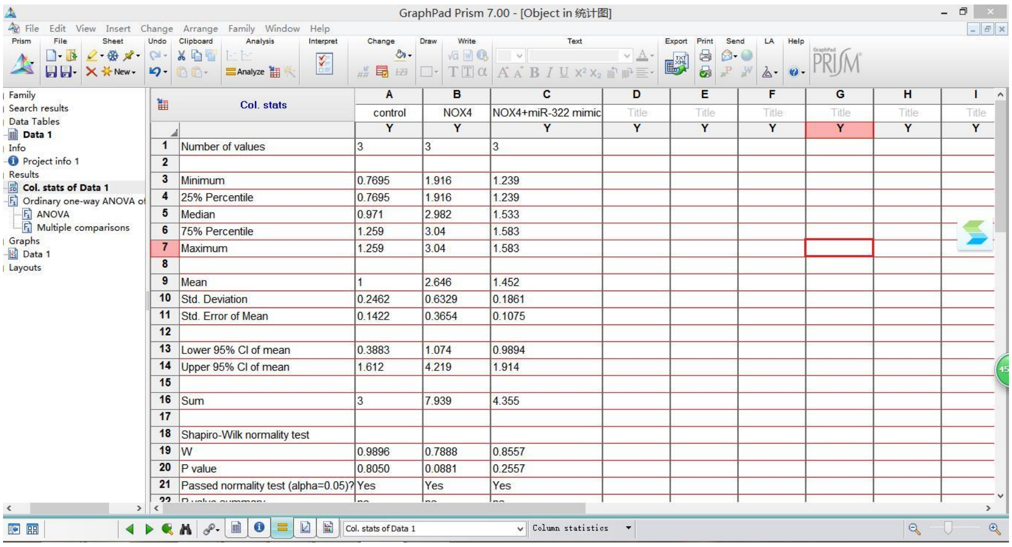

Col. stats	A	B	C
	control	NOX4	NOX4+miR-322 mimic
1 Number of values	3	3	3
3 Minimum	0.7695	1.916	1.239
4 25% Percentile	0.7695	1.916	1.239
5 Median	0.971	2.982	1.533
6 75% Percentile	1.259	3.04	1.583
7 Maximum	1.259	3.04	1.583
9 Mean	1	2.646	1.452
10 Std. Deviation	0.2462	0.6329	0.1861
11 Std. Error of Mean	0.1422	0.3654	0.1075
13 Lower 95% CI of mean	0.3883	1.074	0.9894
14 Upper 95% CI of mean	1.612	4.219	1.914
16 Sum	3	7.939	4.355
18 Shapiro-Wilk normality test			
19 W	0.9896	0.7888	0.8557
20 P value	0.8050	0.0881	0.2557
21 Passed normality test (alpha=0.05)?	Yes	Yes	Yes

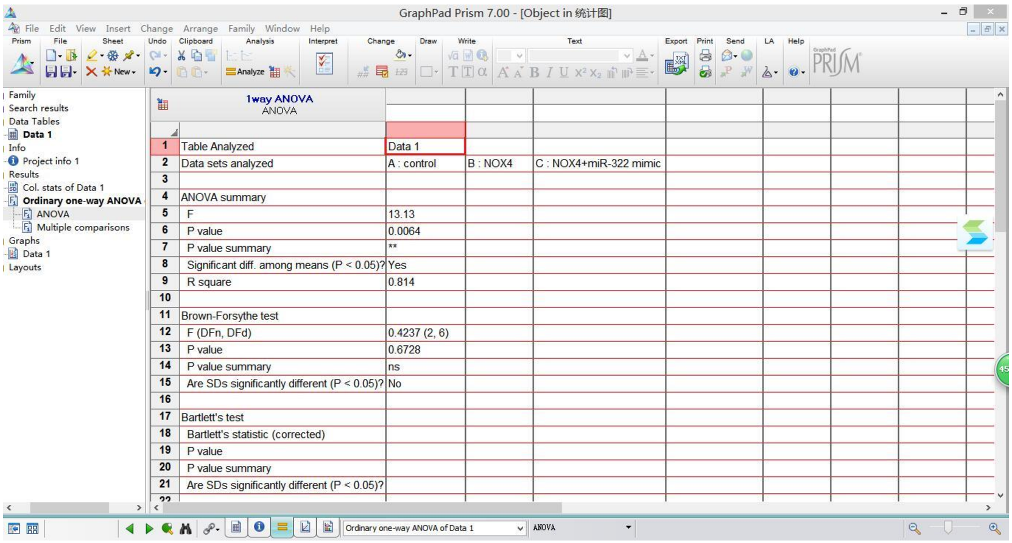

1way ANOVA / ANOVA			
1 Table Analyzed	Data 1		
2 Data sets analyzed	A : control	B : NOX4	C : NOX4+miR-322 mimic
4 ANOVA summary			
5 F	13.13		
6 P value	0.0064		
7 P value summary	**		
8 Significant diff. among means (P < 0.05)?	Yes		
9 R square	0.814		
11 Brown-Forsythe test			
12 F (DFn, DFd)	0.4237 (2, 6)		
13 P value	0.6728		
14 P value summary	ns		
15 Are SDs significantly different (P < 0.05)?	No		
17 Bartlett's test			
18 Bartlett's statistic (corrected)			
19 P value			
20 P value summary			
21 Are SDs significantly different (P < 0.05)?			

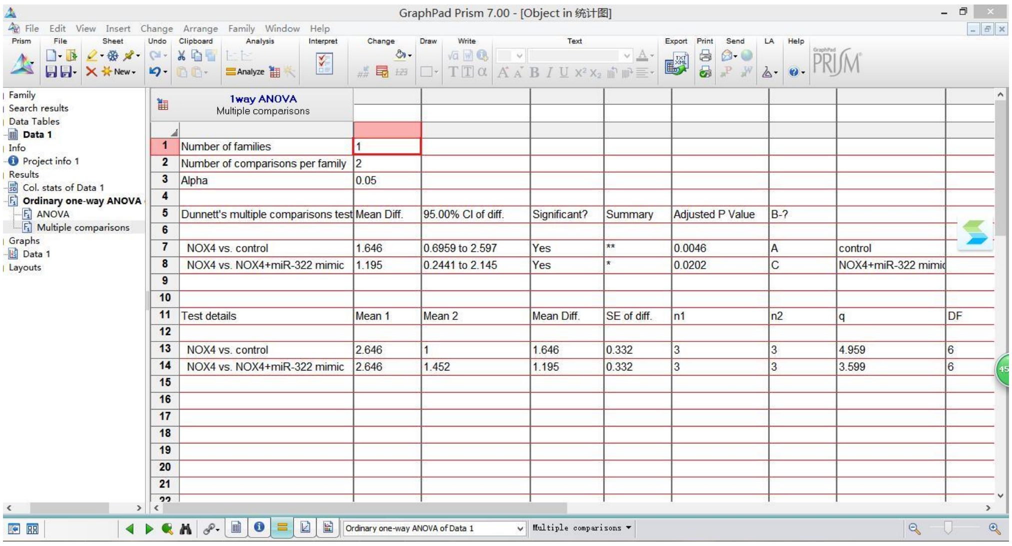

	1way ANOVA Multiple comparisons								
1	Number of families	1							
2	Number of comparisons per family	2							
3	Alpha	0.05							
4									
5	Dunnett's multiple comparisons test	Mean Diff.	95.00% CI of diff.	Significant?	Summary	Adjusted P Value	B-?		
6									
7	NOX4 vs. control	1.646	0.6959 to 2.597	Yes	**	0.0046	A	control	
8	NOX4 vs. NOX4+miR-322 mimic	1.195	0.2441 to 2.145	Yes	*	0.0202	C	NOX4+miR-322 mimic	
9									
10									
11	Test details	Mean 1	Mean 2	Mean Diff.	SE of diff.	n1	n2	q	DF
12									
13	NOX4 vs. control	2.646	1	1.646	0.332	3	3	4.959	6
14	NOX4 vs. NOX4+miR-322 mimic	2.646	1.452	1.195	0.332	3	3	3.599	6
15									
16									
17									
18									
19									
20									
21									

Figure 4B (Cleaved Caspase-3)

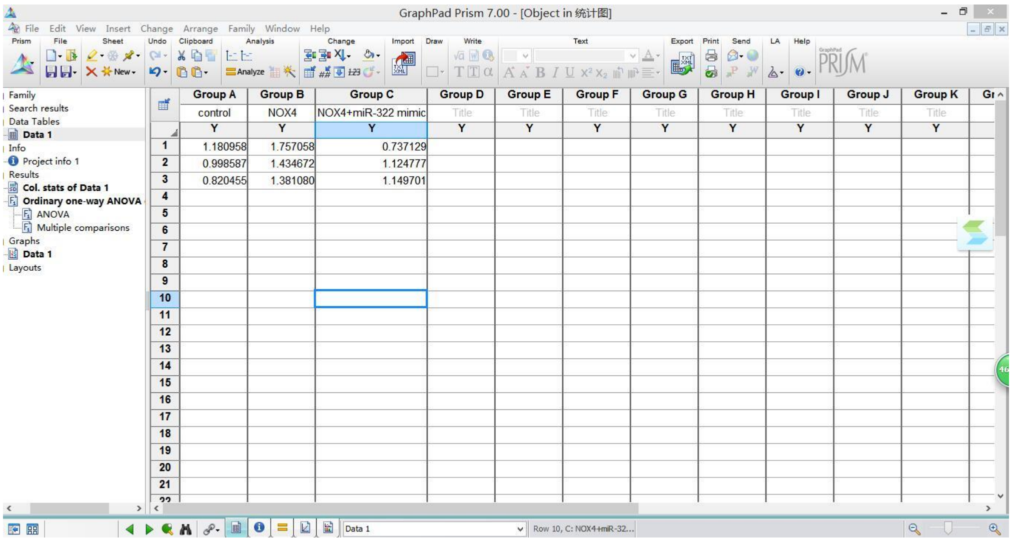

	Group A	Group B	Group C
	control	NOX4	NOX4+miR-322 mimic
	Y	Y	Y
1	1.180958	1.757058	0.737129
2	0.998587	1.434672	1.124777
3	0.820455	1.381080	1.149701

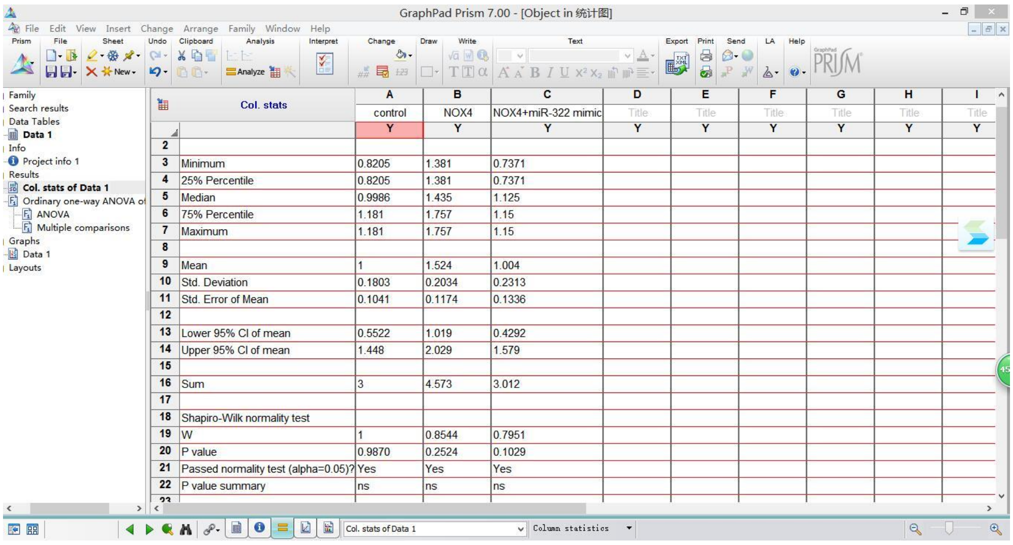

	Col. stats	A	B	C
		control	NOX4	NOX4+miR-322 mimic
		Y	Y	Y
3	Minimum	0.8205	1.381	0.7371
4	25% Percentile	0.8205	1.381	0.7371
5	Median	0.9986	1.435	1.125
6	75% Percentile	1.181	1.757	1.15
7	Maximum	1.181	1.757	1.15
9	Mean	1	1.524	1.004
10	Std. Deviation	0.1803	0.2034	0.2313
11	Std. Error of Mean	0.1041	0.1174	0.1336
13	Lower 95% CI of mean	0.5522	1.019	0.4292
14	Upper 95% CI of mean	1.448	2.029	1.579
16	Sum	3	4.573	3.012
18	Shapiro-Wilk normality test			
19	W	1	0.8544	0.7951
20	P value	0.9870	0.2524	0.1029
21	Passed normality test (alpha=0.05)?	Yes	Yes	Yes
22	P value summary	ns	ns	ns

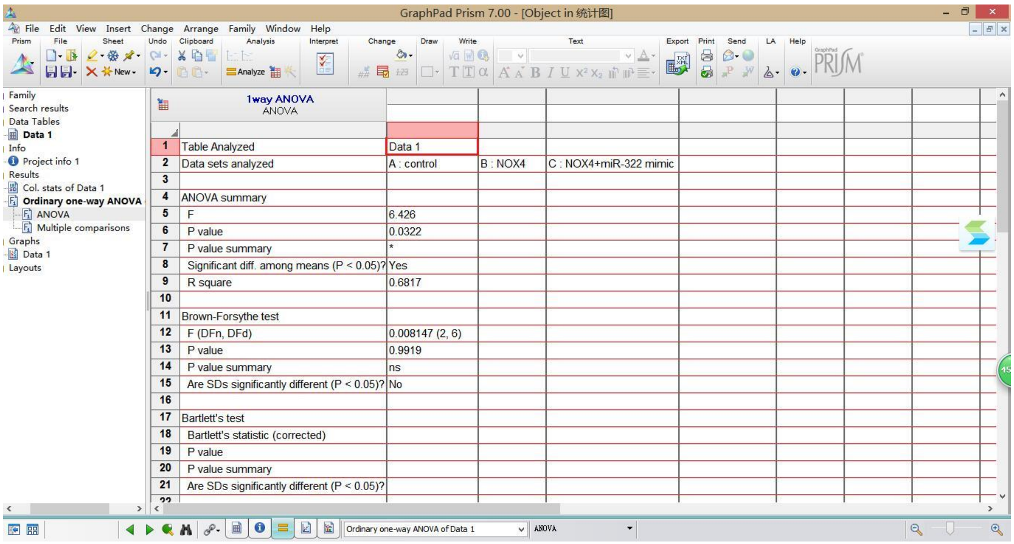

	1way ANOVA ANOVA			
1	Table Analyzed	Data 1		
2	Data sets analyzed	A : control	B : NOX4	C : NOX4+miR-322 mimic
4	ANOVA summary			
5	F	6.426		
6	P value	0.0322		
7	P value summary	*		
8	Significant diff. among means (P < 0.05)?	Yes		
9	R square	0.6817		
11	Brown-Forsythe test			
12	F (DFn, DFd)	0.008147 (2, 6)		
13	P value	0.9919		
14	P value summary	ns		
15	Are SDs significantly different (P < 0.05)?	No		
17	Bartlett's test			
18	Bartlett's statistic (corrected)			
19	P value			
20	P value summary			
21	Are SDs significantly different (P < 0.05)?			

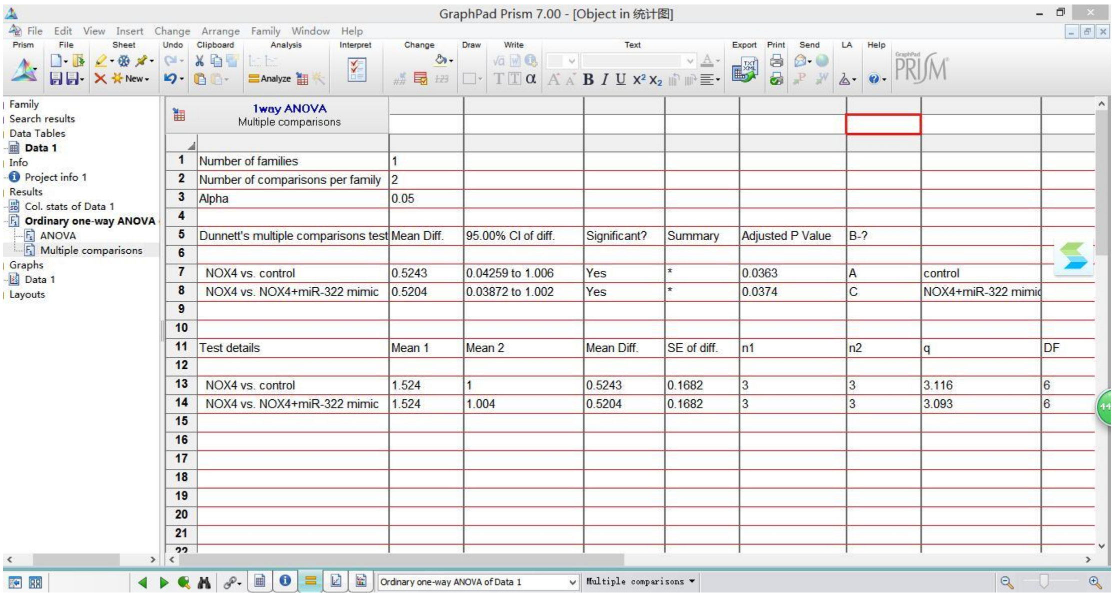

1way ANOVA
Multiple comparisons

1	Number of families	1							
2	Number of comparisons per family	2							
3	Alpha	0.05							
4									
5	Dunnett's multiple comparisons test	Mean Diff.	95.00% CI of diff.	Significant?	Summary	Adjusted P Value	B-?		
6									
7	NOX4 vs. control	0.5243	0.04259 to 1.006	Yes	*	0.0363	A	control	
8	NOX4 vs. NOX4+miR-322 mimic	0.5204	0.03872 to 1.002	Yes	*	0.0374	C	NOX4+miR-322 mimic	
9									
10									
11	Test details	Mean 1	Mean 2	Mean Diff.	SE of diff.	n1	n2	q	DF
12									
13	NOX4 vs. control	1.524	1	0.5243	0.1682	3	3	3.116	6
14	NOX4 vs. NOX4+miR-322 mimic	1.524	1.004	0.5204	0.1682	3	3	3.093	6
15									
16									
17									
18									
19									
20									
21									

Figure 4B (Bax/Bcl-2)

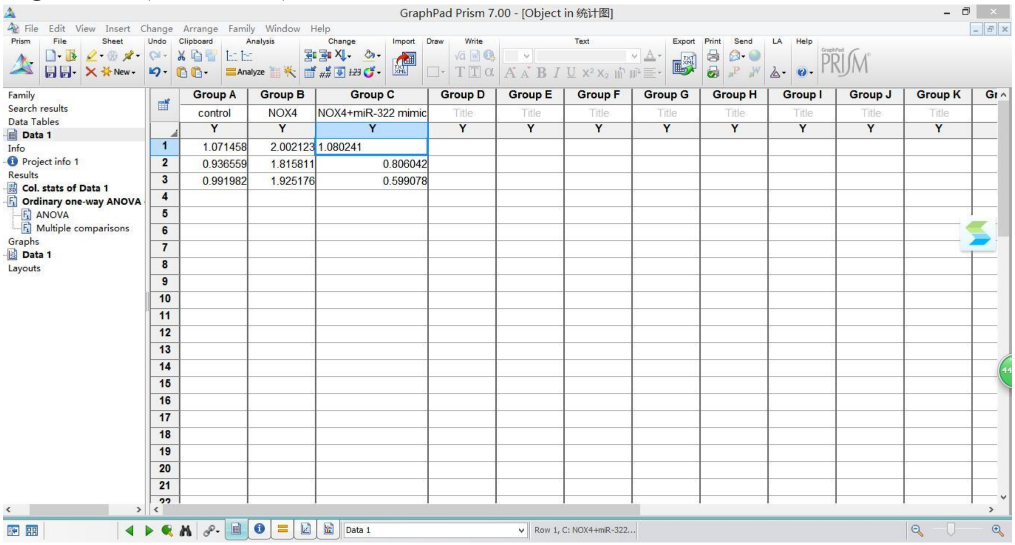

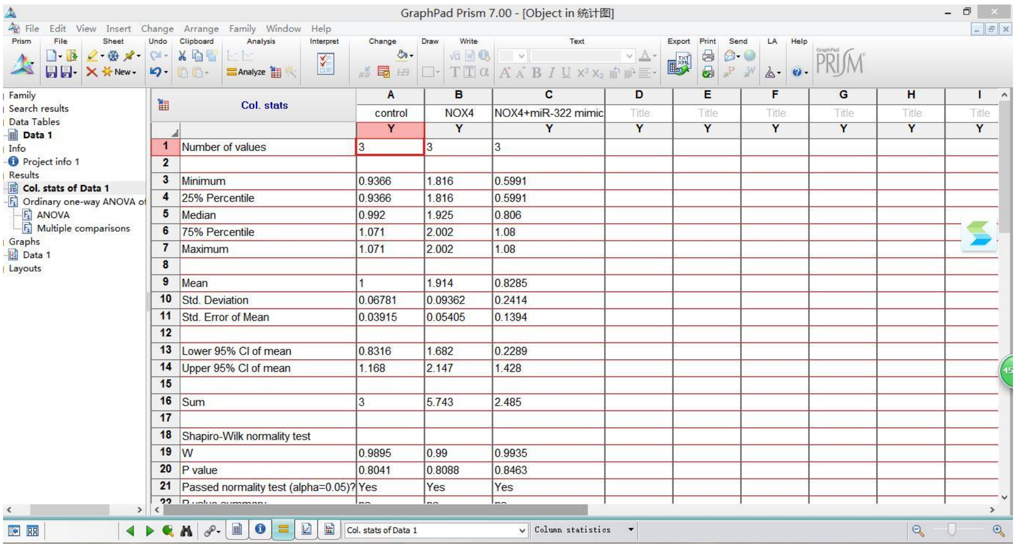

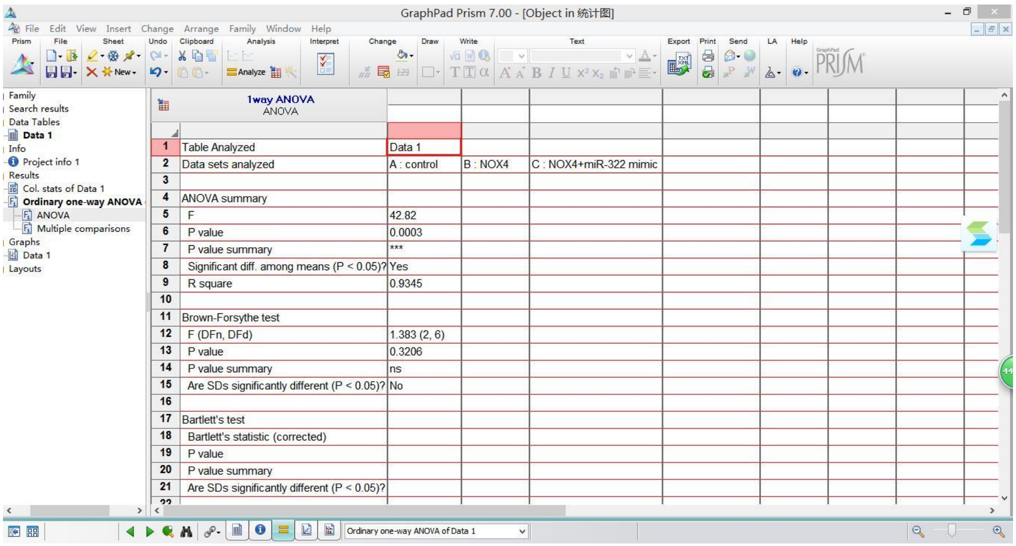

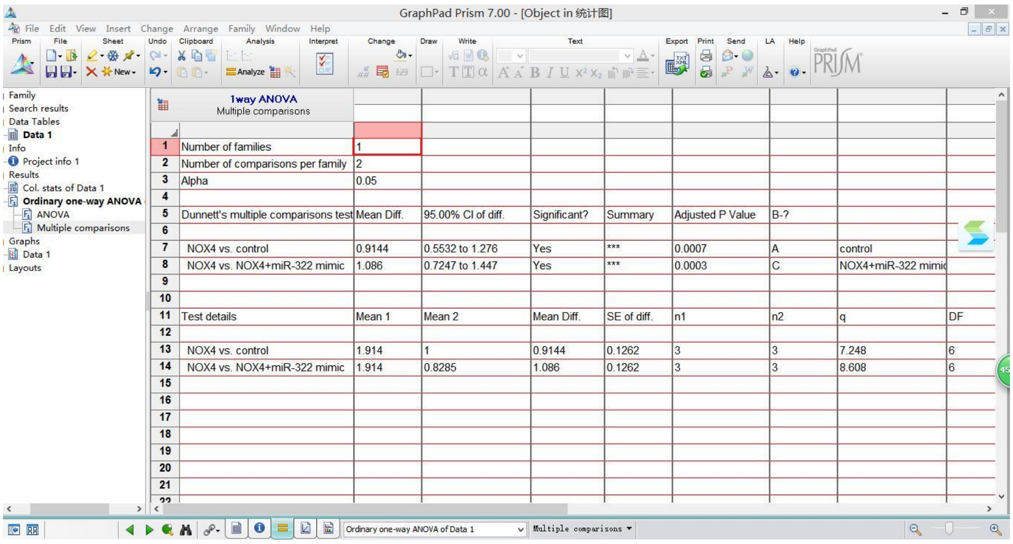

	1way ANOVA Multiple comparisons								
1	Number of families	1							
2	Number of comparisons per family	2							
3	Alpha	0.05							
4									
5	Dunnett's multiple comparisons test	Mean Diff.	95.00% CI of diff.	Significant?	Summary	Adjusted P Value	B-?		
6									
7	NOX4 vs. control	0.9144	0.5532 to 1.276	Yes	***	0.0007	A	control	
8	NOX4 vs. NOX4+miR-322 mimic	1.086	0.7247 to 1.447	Yes	***	0.0003	C	NOX4+miR-322 mimi	
9									
10									
11	Test details	Mean 1	Mean 2	Mean Diff.	SE of diff.	n1	n2	q	DF
12									
13	NOX4 vs. control	1.914	1	0.9144	0.1262	3	3	7.248	6
14	NOX4 vs. NOX4+miR-322 mimic	1.914	0.8285	1.086	0.1262	3	3	8.608	6

Figure 4C

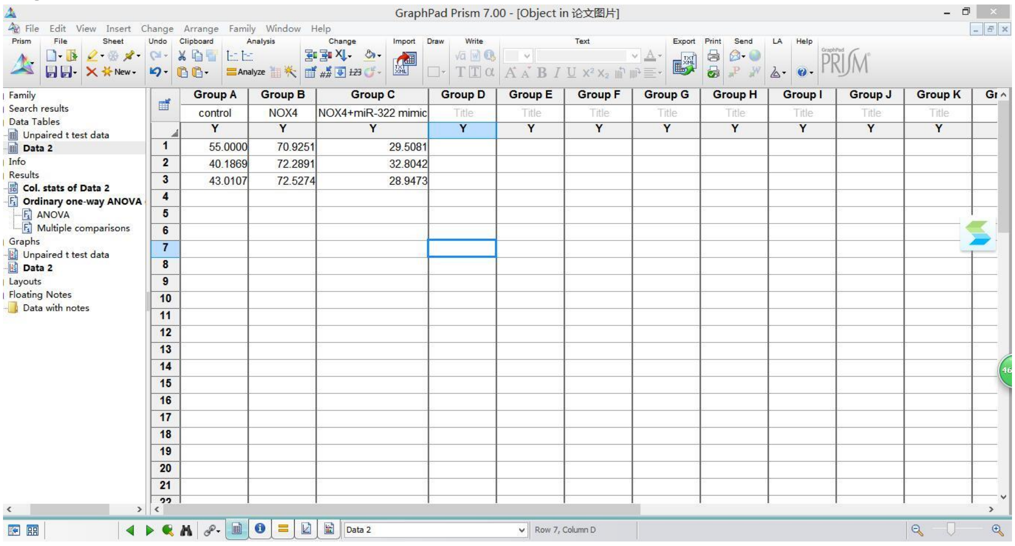

GraphPad Prism 7.00 - [Object in 论文图片]

	Group A	Group B	Group C
	control	NOX4	NOX4+miR-322 mimic
	Y	Y	Y
1	55.0000	70.9251	29.5081
2	40.1869	72.2891	32.8042
3	43.0107	72.5274	28.9473

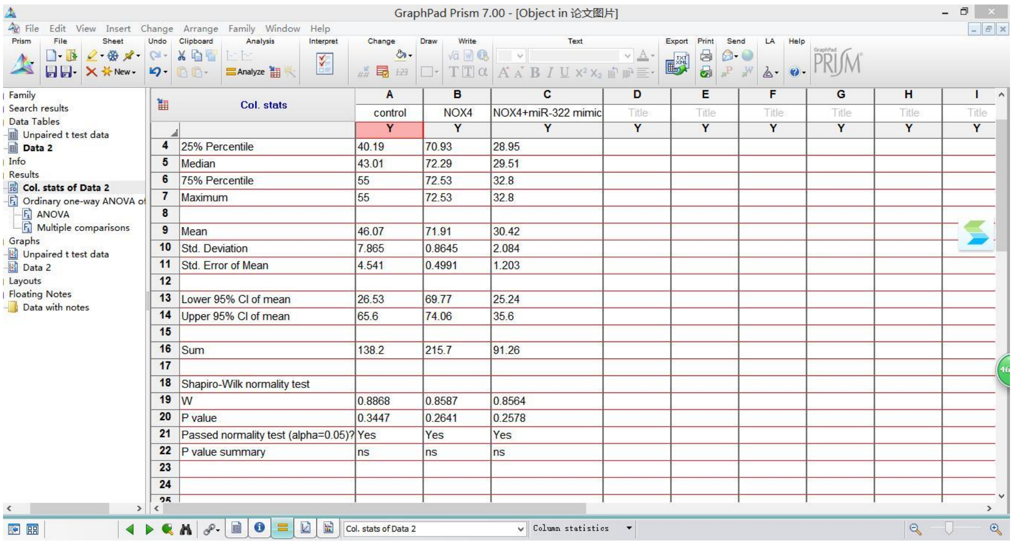

GraphPad Prism 7.00 - [Object in 论文图片]

	Col. stats	A	B	C
		control	NOX4	NOX4+miR-322 mimic
		Y	Y	Y
4	25% Percentile	40.19	70.93	28.95
5	Median	43.01	72.29	29.51
6	75% Percentile	55	72.53	32.8
7	Maximum	55	72.53	32.8
8				
9	Mean	46.07	71.91	30.42
10	Std. Deviation	7.865	0.8645	2.084
11	Std. Error of Mean	4.541	0.4991	1.203
12				
13	Lower 95% CI of mean	26.53	69.77	25.24
14	Upper 95% CI of mean	65.6	74.06	35.6
15				
16	Sum	138.2	215.7	91.26
17				
18	Shapiro-Wilk normality test			
19	W	0.8868	0.8587	0.8564
20	P value	0.3447	0.2641	0.2578
21	Passed normality test (alpha=0.05)?	Yes	Yes	Yes
22	P value summary	ns	ns	ns

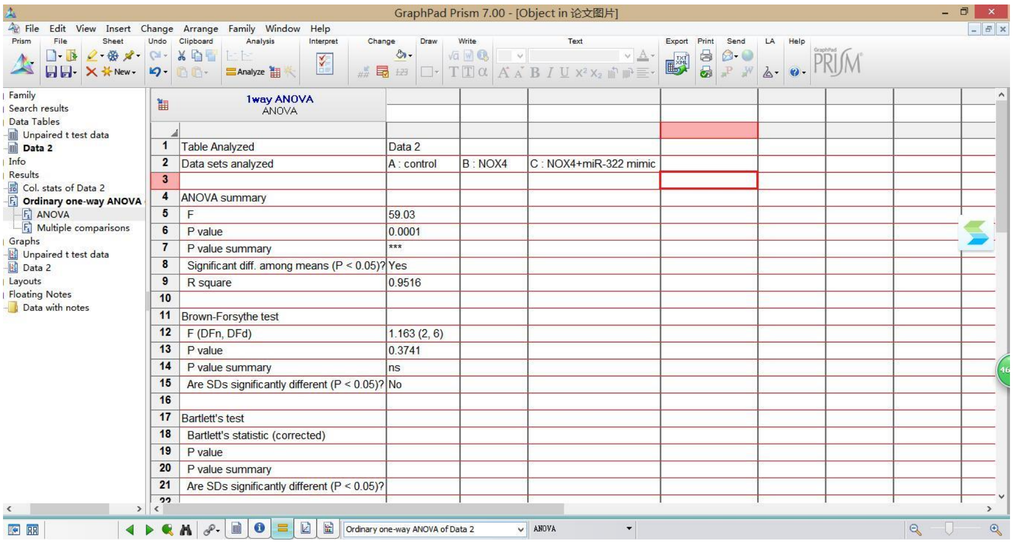

GraphPad Prism 7.00 - [Object in 论文图片]

	1way ANOVA<br>ANOVA			
1	Table Analyzed	Data 2		
2	Data sets analyzed	A : control	B : NOX4	C : NOX4+miR-322 mimic
3				
4	ANOVA summary			
5	F	59.03		
6	P value	0.0001		
7	P value summary	***		
8	Significant diff. among means (P < 0.05)?	Yes		
9	R square	0.9516		
10				
11	Brown-Forsythe test			
12	F (DFn, DFd)	1.163 (2, 6)		
13	P value	0.3741		
14	P value summary	ns		
15	Are SDs significantly different (P < 0.05)?	No		
16				
17	Bartlett's test			
18	Bartlett's statistic (corrected)			
19	P value			
20	P value summary			
21	Are SDs significantly different (P < 0.05)?			

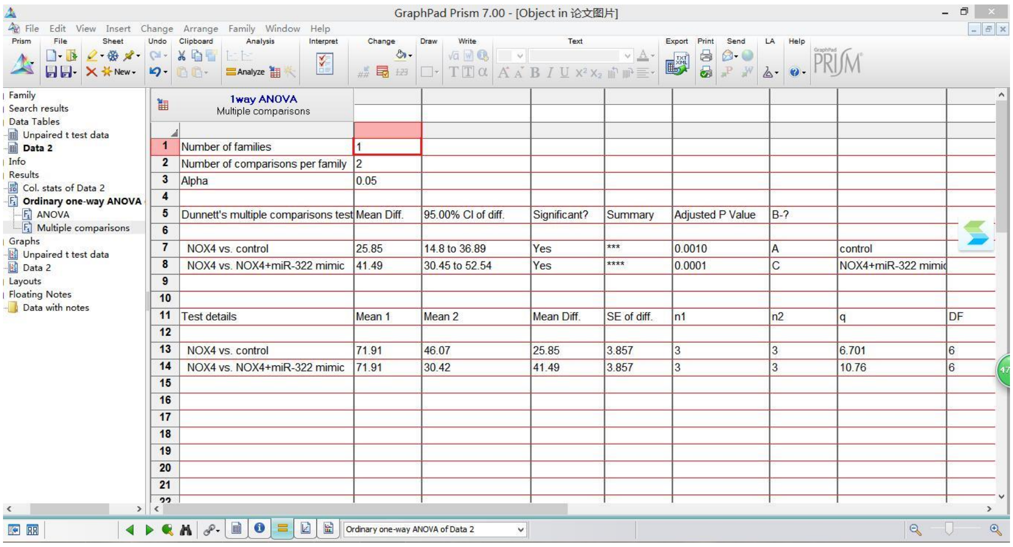

	1way ANOVA Multiple comparisons								
1	Number of families	1							
2	Number of comparisons per family	2							
3	Alpha	0.05							
4									
5	Dunnett's multiple comparisons test	Mean Diff.	95.00% CI of diff.	Significant?	Summary	Adjusted P Value	B-?		
6									
7	NOX4 vs. control	25.85	14.8 to 36.89	Yes	***	0.0010	A	control	
8	NOX4 vs. NOX4+miR-322 mimic	41.49	30.45 to 52.54	Yes	****	0.0001	C	NOX4+miR-322 mimic	
9									
10									
11	Test details	Mean 1	Mean 2	Mean Diff.	SE of diff.	n1	n2	q	DF
12									
13	NOX4 vs. control	71.91	46.07	25.85	3.857	3	3	6.701	6
14	NOX4 vs. NOX4+miR-322 mimic	71.91	30.42	41.49	3.857	3	3	10.76	6

Figure 5B

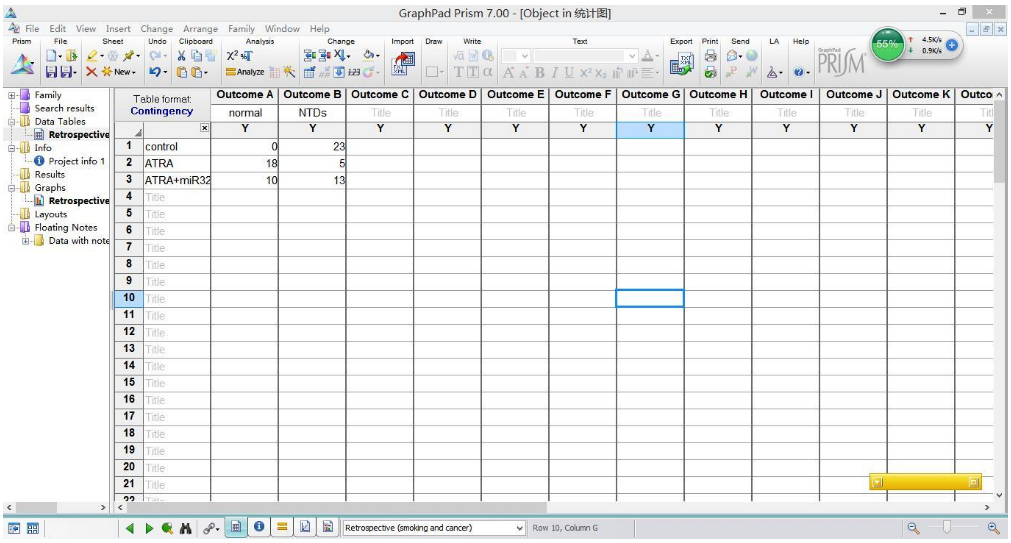

GraphPad Prism 7.00 - [Object in 统计图]
Table format: Contingency
Outcome A normal
Outcome B NTDs
1 control 0 23
2 ATRA 18 5
3 ATRA+miR32 10 13
Retrospective (smoking and cancer)
Row 10, Column G

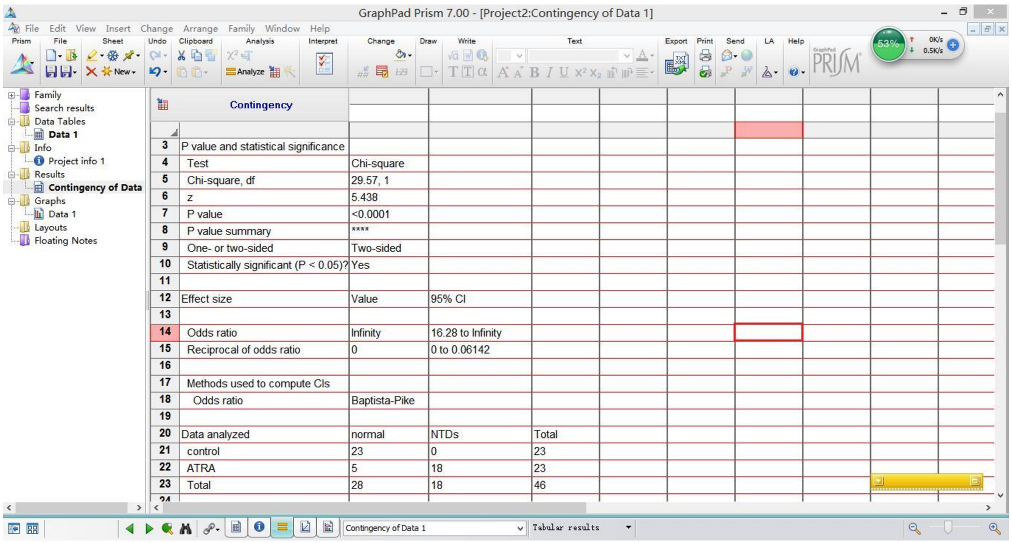

GraphPad Prism 7.00 - [Project2:Contingency of Data 1]
Contingency
3 P value and statistical significance
4 Test Chi-square
5 Chi-square, df 29.57, 1
6 z 5.438
7 P value <0.0001
8 P value summary ****
9 One- or two-sided Two-sided
10 Statistically significant (P < 0.05)? Yes
12 Effect size Value 95% CI
14 Odds ratio Infinity 16.28 to Infinity
15 Reciprocal of odds ratio 0 0 to 0.06142
17 Methods used to compute CIs
18 Odds ratio Baptista-Pike
20 Data analyzed normal NTDs Total
21 control 23 0 23
22 ATRA 5 18 23
23 Total 28 18 46
Contingency of Data 1
Tabular results

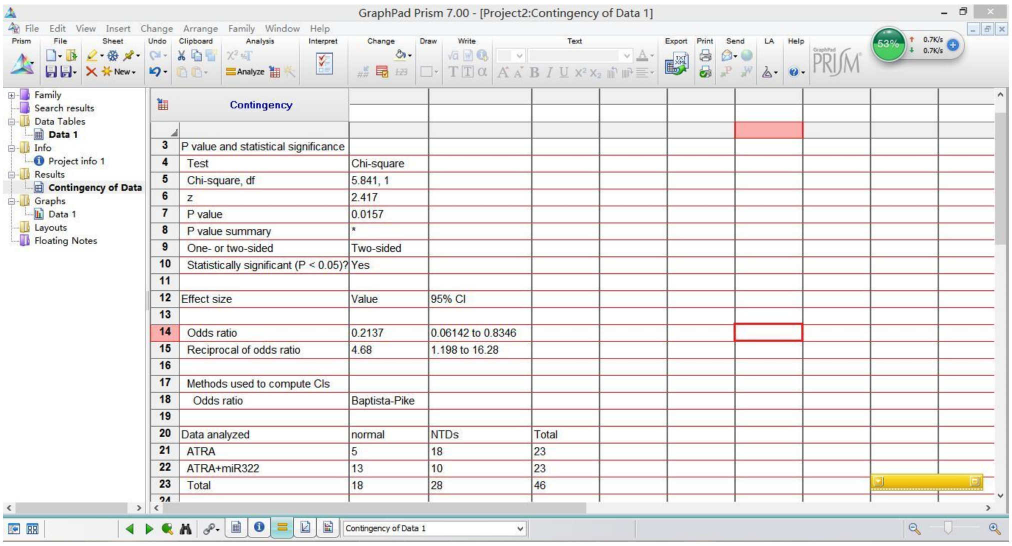

GraphPad Prism 7.00 - [Project2:Contingency of Data 1]
Contingency
3 P value and statistical significance
4 Test Chi-square
5 Chi-square, df 5.841, 1
6 z 2.417
7 P value 0.0157
8 P value summary *
9 One- or two-sided Two-sided
10 Statistically significant (P < 0.05)? Yes
12 Effect size Value 95% CI
14 Odds ratio 0.2137 0.06142 to 0.8346
15 Reciprocal of odds ratio 4.68 1.198 to 16.28
17 Methods used to compute CIs
18 Odds ratio Baptista-Pike
20 Data analyzed normal NTDs Total
21 ATRA 5 18 23
22 ATRA+miR322 13 10 23
23 Total 18 28 46
Contingency of Data 1

Figure 5C

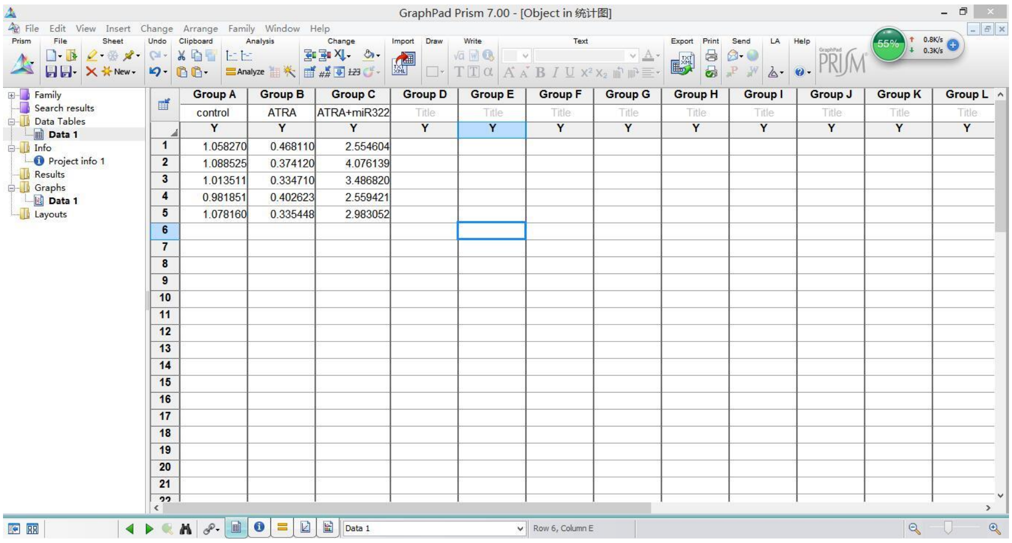

	Group A	Group B	Group C
	control	ATRA	ATRA+miR322
	Y	Y	Y
1	1.058270	0.468110	2.554604
2	1.088525	0.374120	4.076139
3	1.013511	0.334710	3.486820
4	0.981851	0.402623	2.559421
5	1.078160	0.335448	2.983052

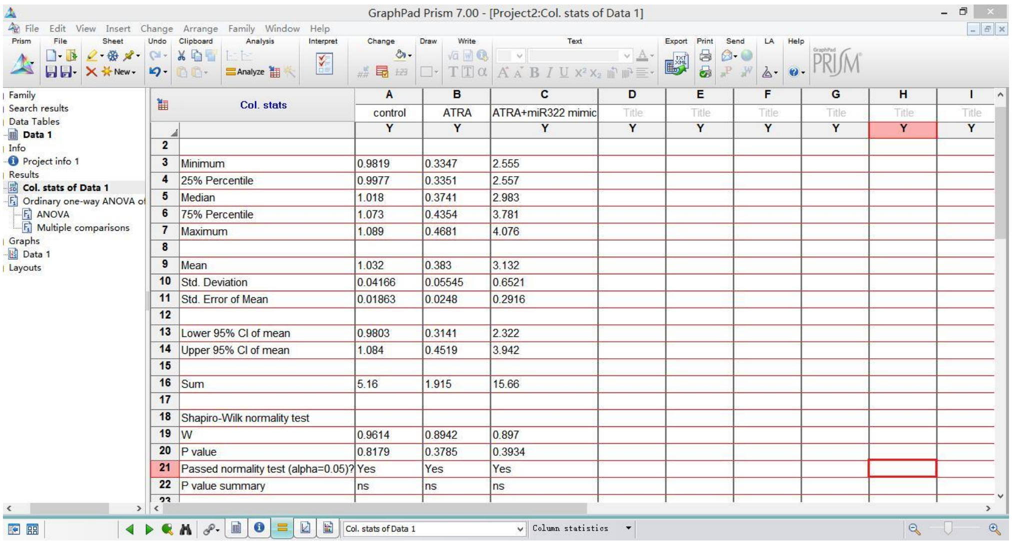

	Col. stats	A	B	C
		control	ATRA	ATRA+miR322 mimic
		Y	Y	Y
3	Minimum	0.9819	0.3347	2.555
4	25% Percentile	0.9977	0.3351	2.557
5	Median	1.018	0.3741	2.983
6	75% Percentile	1.073	0.4354	3.781
7	Maximum	1.089	0.4681	4.076
9	Mean	1.032	0.383	3.132
10	Std. Deviation	0.04166	0.05545	0.6521
11	Std. Error of Mean	0.01863	0.0248	0.2916
13	Lower 95% CI of mean	0.9803	0.3141	2.322
14	Upper 95% CI of mean	1.084	0.4519	3.942
16	Sum	5.16	1.915	15.66
18	Shapiro-Wilk normality test			
19	W	0.9614	0.8942	0.897
20	P value	0.8179	0.3785	0.3934
21	Passed normality test (alpha=0.05)?	Yes	Yes	Yes
22	P value summary	ns	ns	ns

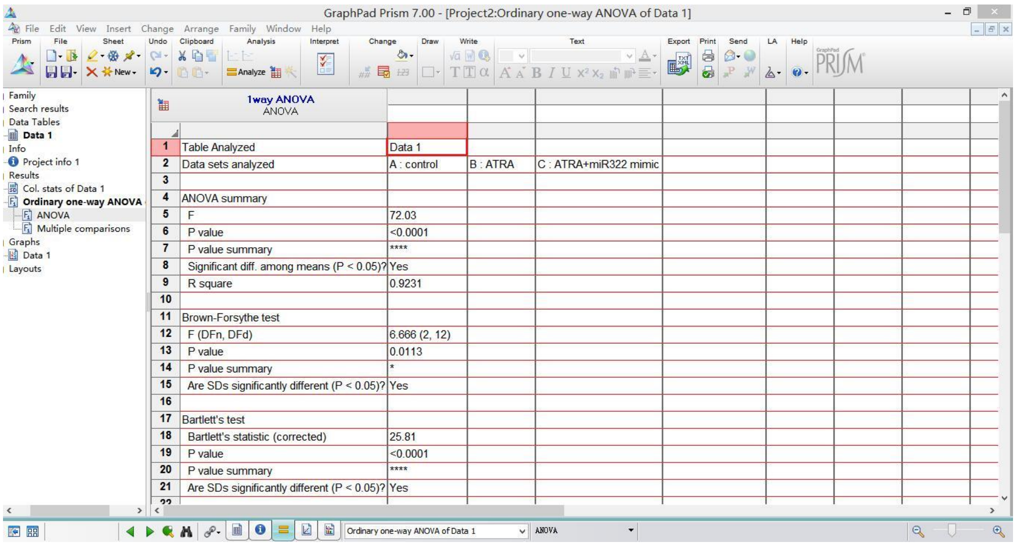

	1way ANOVA ANOVA			
1	Table Analyzed	Data 1		
2	Data sets analyzed	A : control	B : ATRA	C : ATRA+miR322 mimic
4	ANOVA summary			
5	F	72.03		
6	P value	<0.0001		
7	P value summary	****		
8	Significant diff. among means (P < 0.05)?	Yes		
9	R square	0.9231		
11	Brown-Forsythe test			
12	F (DFn, DFd)	6.666 (2, 12)		
13	P value	0.0113		
14	P value summary	*		
15	Are SDs significantly different (P < 0.05)?	Yes		
17	Bartlett's test			
18	Bartlett's statistic (corrected)	25.81		
19	P value	<0.0001		
20	P value summary	****		
21	Are SDs significantly different (P < 0.05)?	Yes		

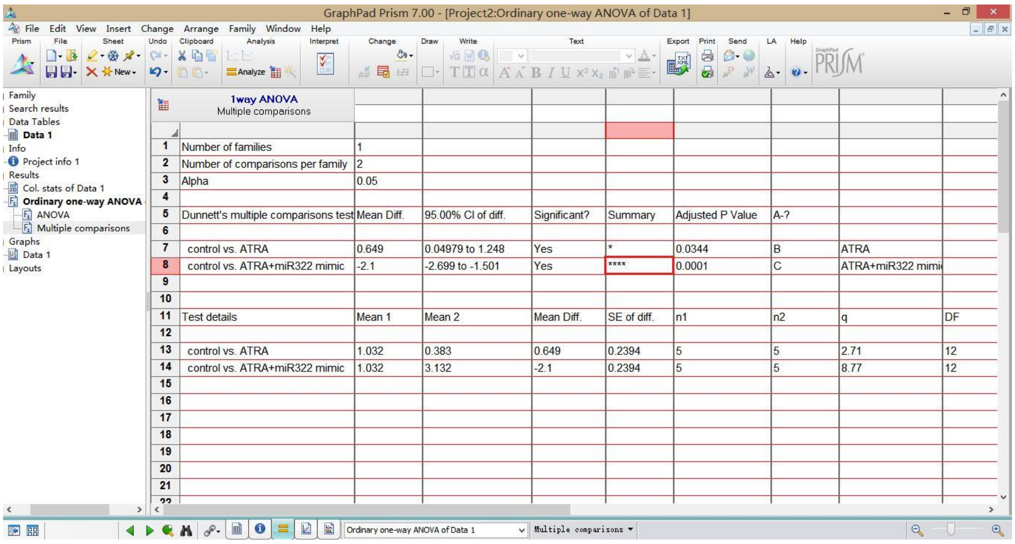

1	Number of families	1							
2	Number of comparisons per family	2							
3	Alpha	0.05							
4									
5	Dunnett's multiple comparisons test	Mean Diff.	95.00% CI of diff.	Significant?	Summary	Adjusted P Value	A-?		
6									
7	control vs. ATRA	0.649	0.04979 to 1.248	Yes	*	0.0344	B	ATRA	
8	control vs. ATRA+miR322 mimic	-2.1	-2.699 to -1.501	Yes	****	0.0001	C	ATRA+miR322 mimi	
9									
10									
11	Test details	Mean 1	Mean 2	Mean Diff.	SE of diff.	n1	n2	q	DF
12									
13	control vs. ATRA	1.032	0.383	0.649	0.2394	5	5	2.71	12
14	control vs. ATRA+miR322 mimic	1.032	3.132	-2.1	0.2394	5	5	8.77	12

Figure 5D (NOX4)

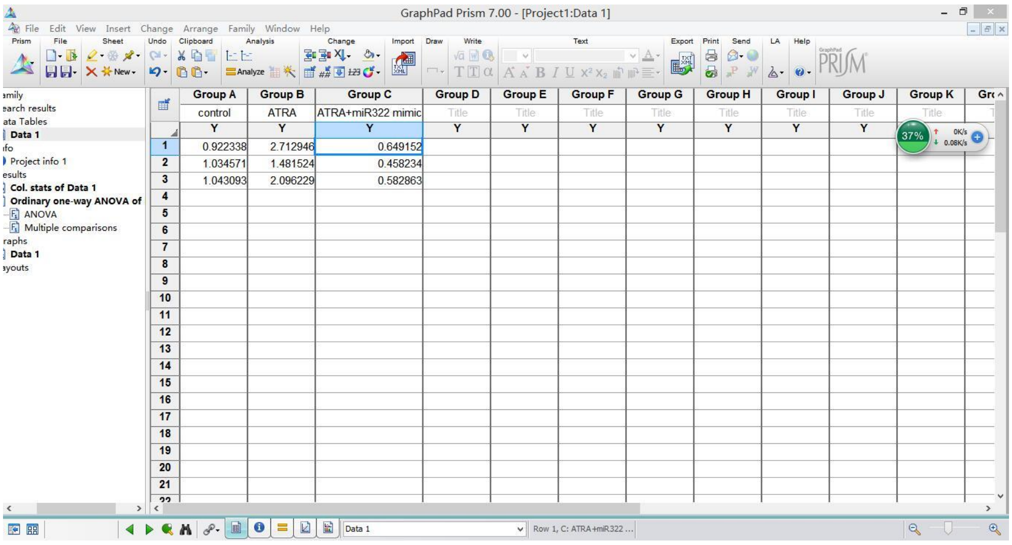

GraphPad Prism 7.00 - [Project1:Data 1]

	Group A	Group B	Group C
	control	ATRA	ATRA+miR322 mimic
	Y	Y	Y
1	0.922338	2.712946	0.649152
2	1.034571	1.481524	0.458234
3	1.043093	2.096229	0.582863

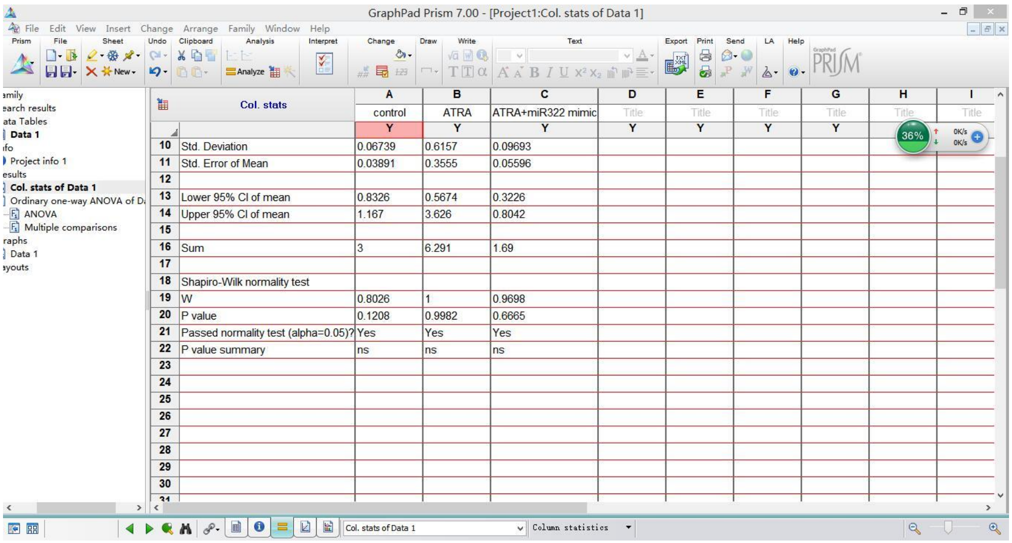

GraphPad Prism 7.00 - [Project1:Col. stats of Data 1]

Col. stats	A	B	C
	control	ATRA	ATRA+miR322 mimic
	Y	Y	Y
10 Std. Deviation	0.06739	0.6157	0.09693
11 Std. Error of Mean	0.03891	0.3555	0.05596
12			
13 Lower 95% CI of mean	0.8326	0.5674	0.3226
14 Upper 95% CI of mean	1.167	3.626	0.8042
15			
16 Sum	3	6.291	1.69
17			
18 Shapiro-Wilk normality test			
19 W	0.8026	1	0.9698
20 P value	0.1208	0.9982	0.6665
21 Passed normality test (alpha=0.05)?	Yes	Yes	Yes
22 P value summary	ns	ns	ns

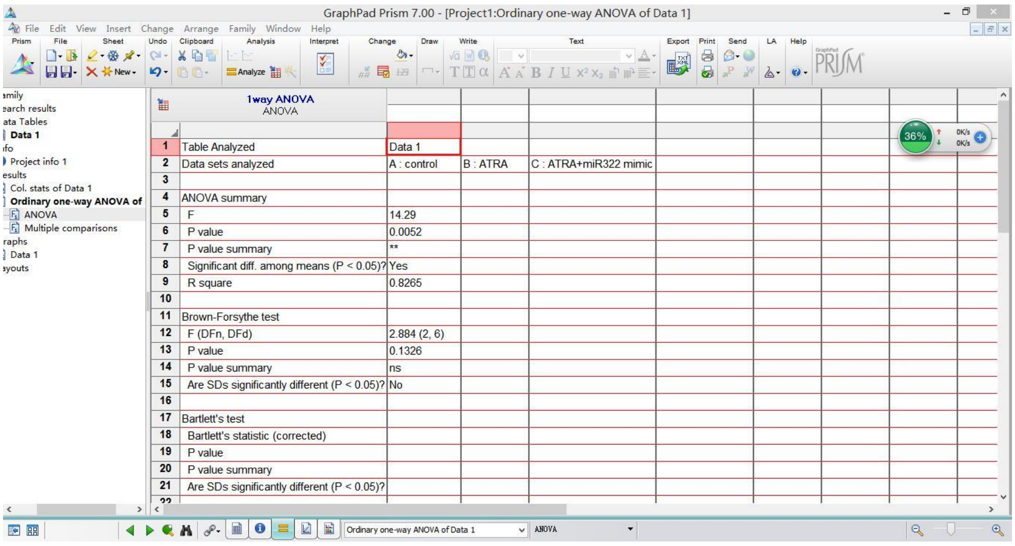

GraphPad Prism 7.00 - [Project1:Ordinary one-way ANOVA of Data 1]

1way ANOVA / ANOVA			
1 Table Analyzed	Data 1		
2 Data sets analyzed	A : control	B : ATRA	C : ATRA+miR322 mimic
3			
4 ANOVA summary			
5 F	14.29		
6 P value	0.0052		
7 P value summary	**		
8 Significant diff. among means (P < 0.05)?	Yes		
9 R square	0.8265		
10			
11 Brown-Forsythe test			
12 F (DFn, DFd)	2.884 (2, 6)		
13 P value	0.1326		
14 P value summary	ns		
15 Are SDs significantly different (P < 0.05)?	No		
16			
17 Bartlett's test			
18 Bartlett's statistic (corrected)			
19 P value			
20 P value summary			
21 Are SDs significantly different (P < 0.05)?			

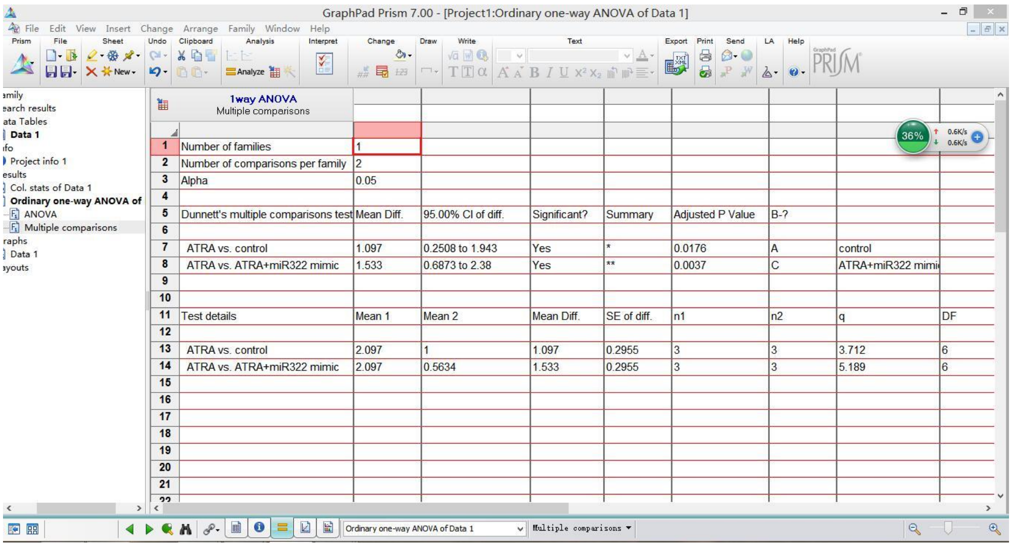

1	Number of families	1							
2	Number of comparisons per family	2							
3	Alpha	0.05							
4									
5	Dunnett's multiple comparisons test	Mean Diff.	95.00% CI of diff.	Significant?	Summary	Adjusted P Value	B-?		
6									
7	ATRA vs. control	1.097	0.2508 to 1.943	Yes	*	0.0176	A	control	
8	ATRA vs. ATRA+miR322 mimic	1.533	0.6873 to 2.38	Yes	**	0.0037	C	ATRA+miR322 mimi	
9									
10									
11	Test details	Mean 1	Mean 2	Mean Diff.	SE of diff.	n1	n2	q	DF
12									
13	ATRA vs. control	2.097	1	1.097	0.2955	3	3	3.712	6
14	ATRA vs. ATRA+miR322 mimic	2.097	0.5634	1.533	0.2955	3	3	5.189	6

Figure 5D (Cleaved Caspase-3)

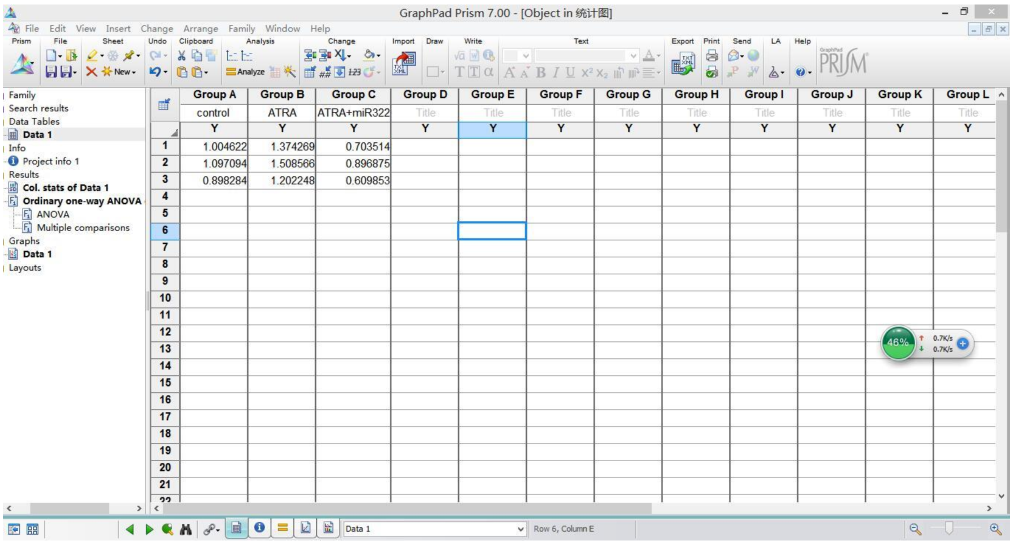

GraphPad Prism 7.00 - [Object in 统计图]

	Group A	Group B	Group C
	control	ATRA	ATRA+miR322
	Y	Y	Y
1	1.004622	1.374269	0.703514
2	1.097094	1.508566	0.896875
3	0.898284	1.202248	0.609853

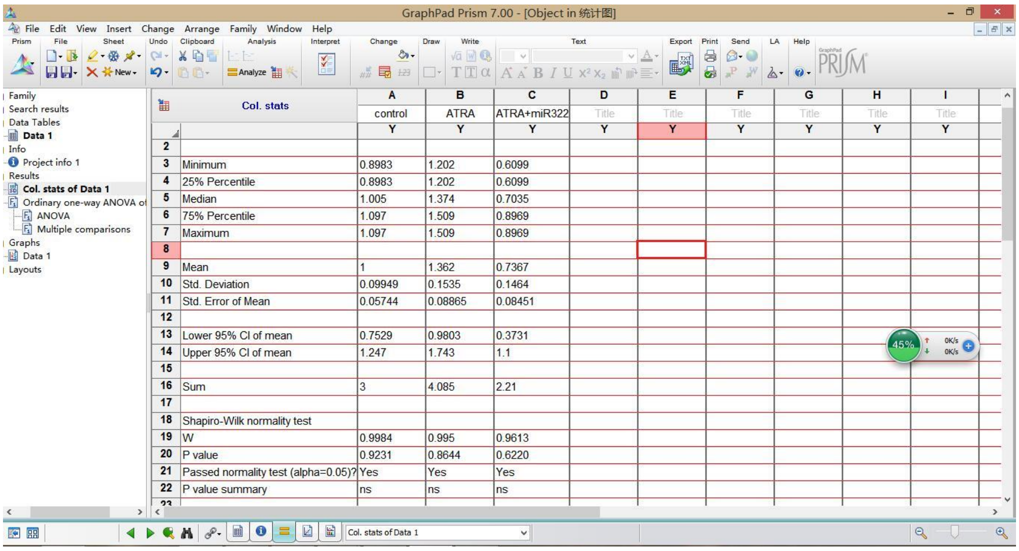

GraphPad Prism 7.00 - [Object in 统计图]

	Col. stats	A	B	C
		control	ATRA	ATRA+miR322
		Y	Y	Y
2				
3	Minimum	0.8983	1.202	0.6099
4	25% Percentile	0.8983	1.202	0.6099
5	Median	1.005	1.374	0.7035
6	75% Percentile	1.097	1.509	0.8969
7	Maximum	1.097	1.509	0.8969
8				
9	Mean	1	1.362	0.7367
10	Std. Deviation	0.09949	0.1535	0.1464
11	Std. Error of Mean	0.05744	0.08865	0.08451
12				
13	Lower 95% CI of mean	0.7529	0.9803	0.3731
14	Upper 95% CI of mean	1.247	1.743	1.1
15				
16	Sum	3	4.085	2.21
17				
18	Shapiro-Wilk normality test			
19	W	0.9984	0.995	0.9613
20	P value	0.9231	0.8644	0.6220
21	Passed normality test (alpha=0.05)?	Yes	Yes	Yes
22	P value summary	ns	ns	ns

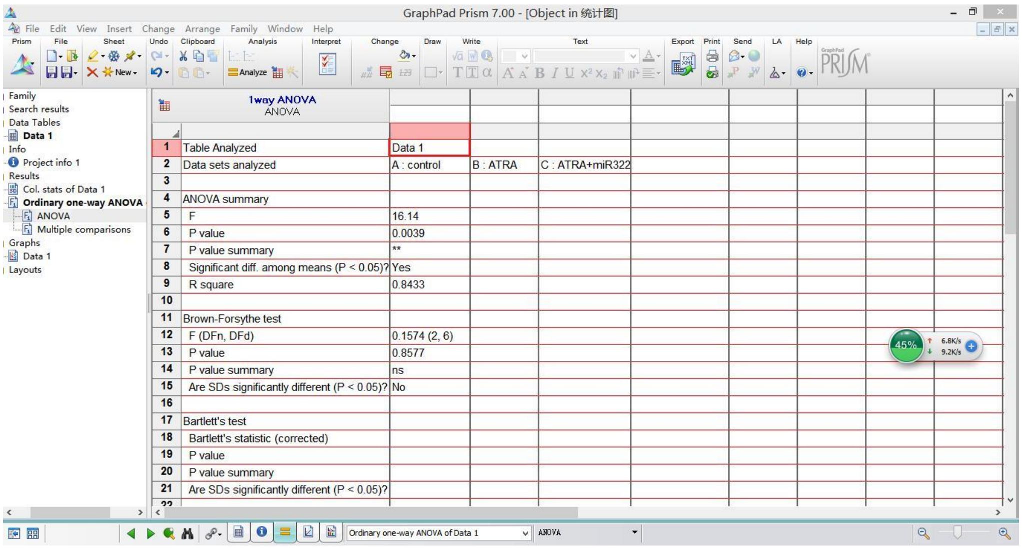

GraphPad Prism 7.00 - [Object in 统计图]

	1way ANOVA ANOVA			
1	Table Analyzed	Data 1		
2	Data sets analyzed	A : control	B : ATRA	C : ATRA+miR322
3				
4	ANOVA summary			
5	F	16.14		
6	P value	0.0039		
7	P value summary	**		
8	Significant diff. among means (P < 0.05)?	Yes		
9	R square	0.8433		
10				
11	Brown-Forsythe test			
12	F (DFn, DFd)	0.1574 (2, 6)		
13	P value	0.8577		
14	P value summary	ns		
15	Are SDs significantly different (P < 0.05)?	No		
16				
17	Bartlett's test			
18	Bartlett's statistic (corrected)			
19	P value			
20	P value summary			
21	Are SDs significantly different (P < 0.05)?			

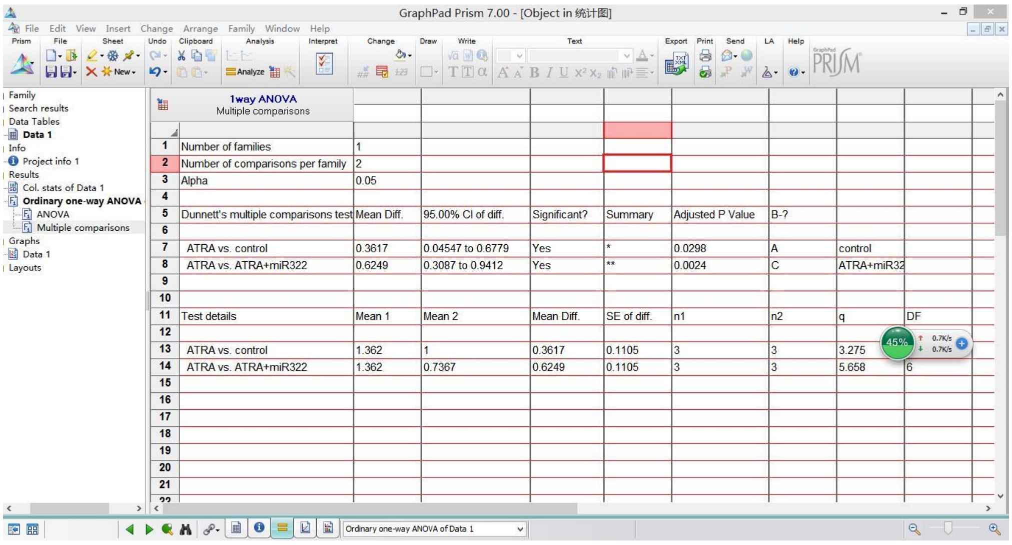

1	Number of families	1							
2	Number of comparisons per family	2							
3	Alpha	0.05							
4									
5	Dunnett's multiple comparisons test	Mean Diff.	95.00% CI of diff.	Significant?	Summary	Adjusted P Value	B-?		
6									
7	ATRA vs. control	0.3617	0.04547 to 0.6779	Yes	*	0.0298	A	control	
8	ATRA vs. ATRA+miR322	0.6249	0.3087 to 0.9412	Yes	**	0.0024	C	ATRA+miR32	
9									
10									
11	Test details	Mean 1	Mean 2	Mean Diff.	SE of diff.	n1	n2	q	DF
12									
13	ATRA vs. control	1.362	1	0.3617	0.1105	3	3	3.275	[illegible]
14	ATRA vs. ATRA+miR322	1.362	0.7367	0.6249	0.1105	3	3	5.658	6

Figure 5D (Bax/Bcl-2)

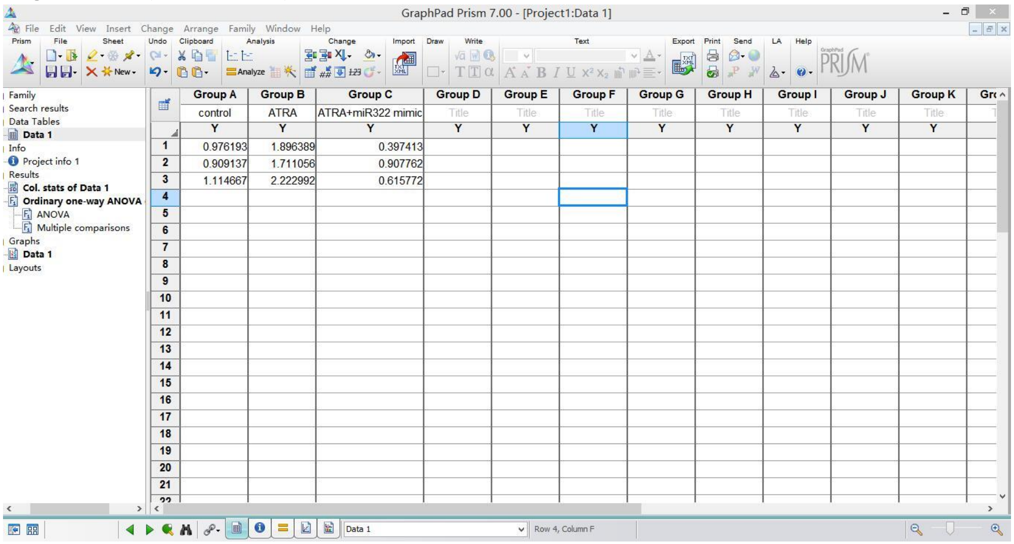

	Group A	Group B	Group C
	control	ATRA	ATRA+miR322 mimic
	Y	Y	Y
1	0.976193	1.896389	0.397413
2	0.909137	1.711056	0.907762
3	1.114667	2.222992	0.615772

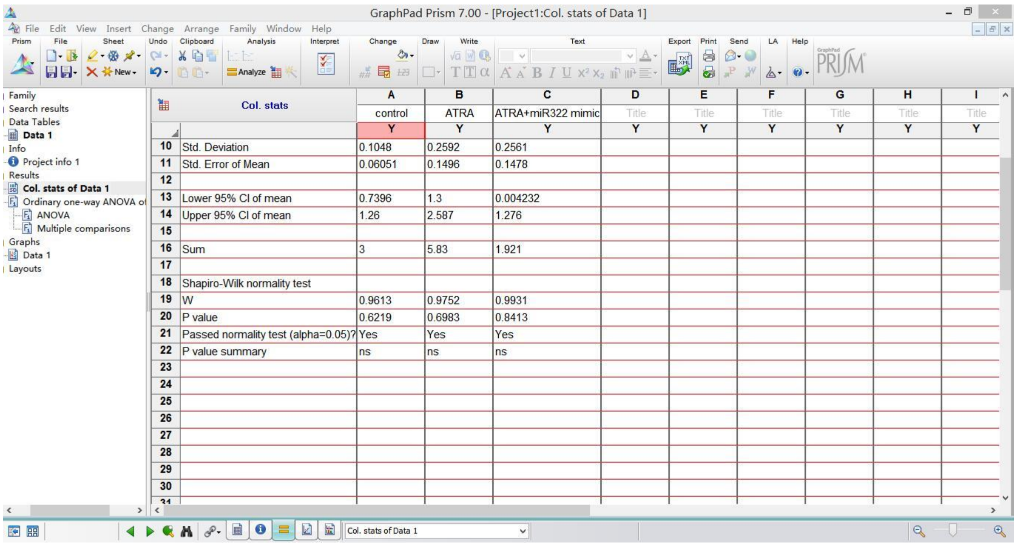

	Col. stats	A	B	C
		control	ATRA	ATRA+miR322 mimic
		Y	Y	Y
10	Std. Deviation	0.1048	0.2592	0.2561
11	Std. Error of Mean	0.06051	0.1496	0.1478
12				
13	Lower 95% CI of mean	0.7396	1.3	0.004232
14	Upper 95% CI of mean	1.26	2.587	1.276
15				
16	Sum	3	5.83	1.921
17				
18	Shapiro-Wilk normality test			
19	W	0.9613	0.9752	0.9931
20	P value	0.6219	0.6983	0.8413
21	Passed normality test (alpha=0.05)?	Yes	Yes	Yes
22	P value summary	ns	ns	ns

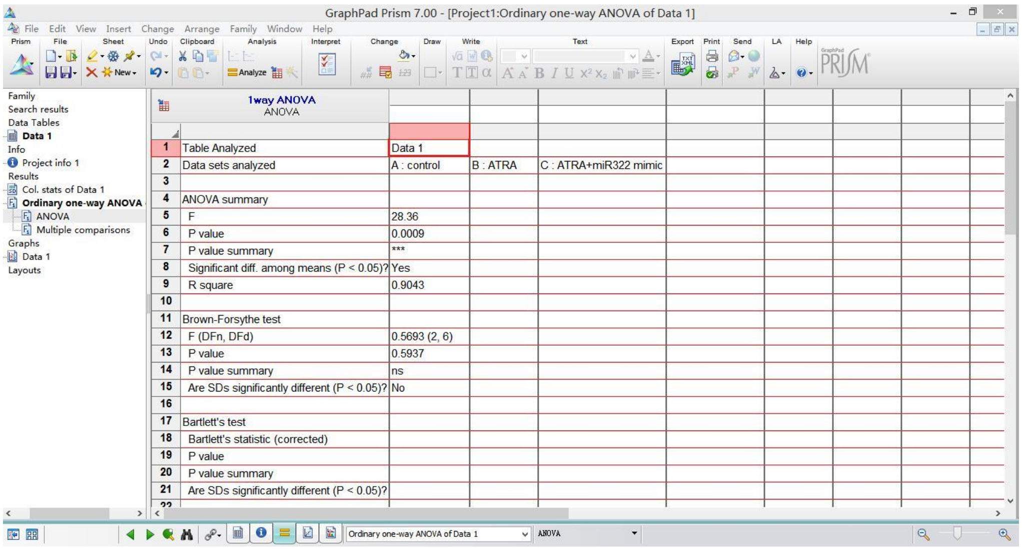

	1way ANOVA<br>ANOVA			
1	Table Analyzed	Data 1		
2	Data sets analyzed	A : control	B : ATRA	C : ATRA+miR322 mimic
3				
4	ANOVA summary			
5	F	28.36		
6	P value	0.0009		
7	P value summary	***		
8	Significant diff. among means (P < 0.05)?	Yes		
9	R square	0.9043		
10				
11	Brown-Forsythe test			
12	F (DFn, DFd)	0.5693 (2, 6)		
13	P value	0.5937		
14	P value summary	ns		
15	Are SDs significantly different (P < 0.05)?	No		
16				
17	Bartlett's test			
18	Bartlett's statistic (corrected)			
19	P value			
20	P value summary			
21	Are SDs significantly different (P < 0.05)?			

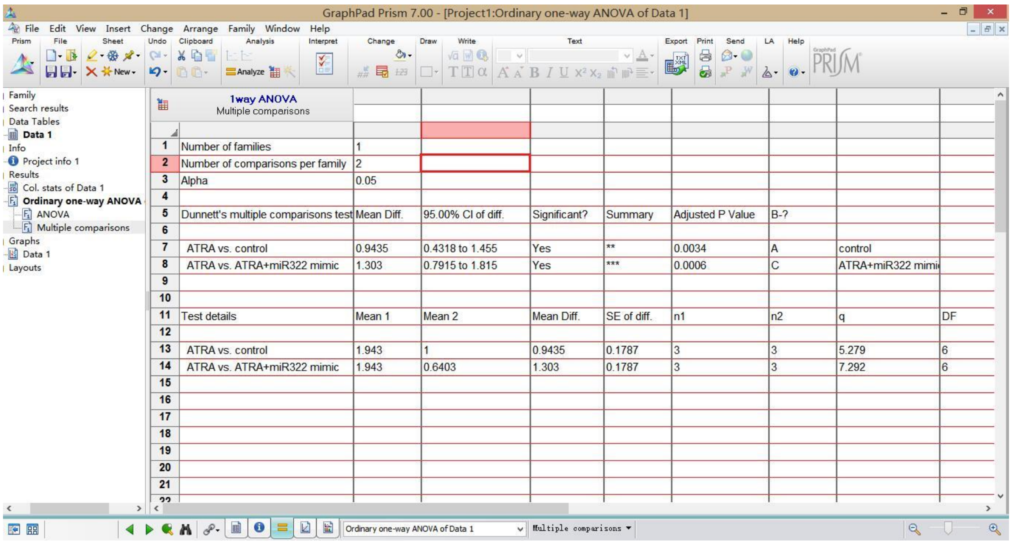

GraphPad Prism 7.00 - [Project1:Ordinary one-way ANOVA of Data 1]

1way ANOVA
Multiple comparisons

1	Number of families	1							
2	Number of comparisons per family	2							
3	Alpha	0.05							
4									
5	Dunnett's multiple comparisons test	Mean Diff.	95.00% CI of diff.	Significant?	Summary	Adjusted P Value	B-?		
6									
7	ATRA vs. control	0.9435	0.4318 to 1.455	Yes	**	0.0034	A	control	
8	ATRA vs. ATRA+miR322 mimic	1.303	0.7915 to 1.815	Yes	***	0.0006	C	ATRA+miR322 mimi	
9									
10									
11	Test details	Mean 1	Mean 2	Mean Diff.	SE of diff.	n1	n2	q	DF
12									
13	ATRA vs. control	1.943	1	0.9435	0.1787	3	3	5.279	6
14	ATRA vs. ATRA+miR322 mimic	1.943	0.6403	1.303	0.1787	3	3	7.292	6
15									
16									
17									
18									
19									
20									
21									

Figure 5E

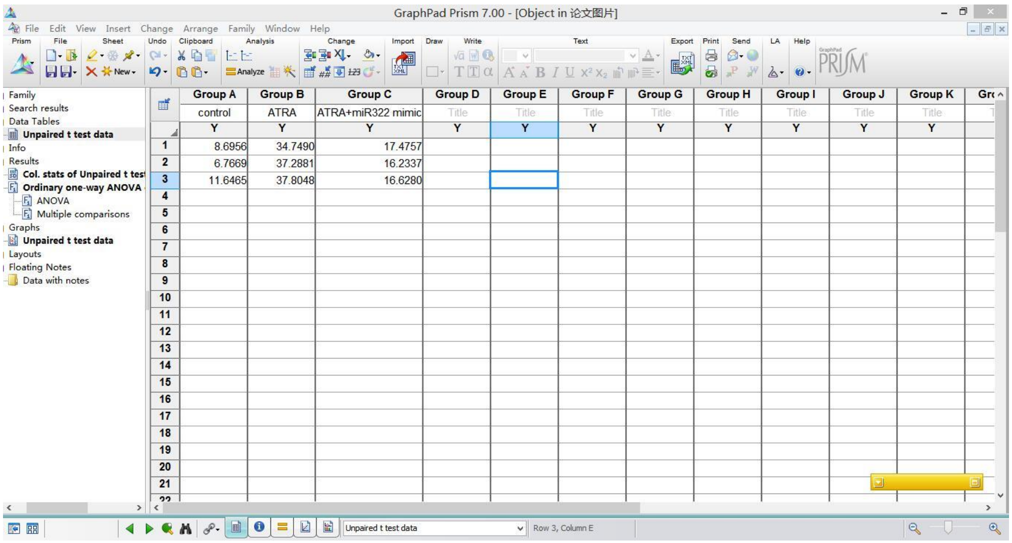

GraphPad Prism 7.00 - [Object in 论文图片]

	Group A	Group B	Group C
	control	ATRA	ATRA+miR322 mimic
	Y	Y	Y
1	8.6956	34.7490	17.4757
2	6.7669	37.2881	16.2337
3	11.6465	37.8048	16.6280

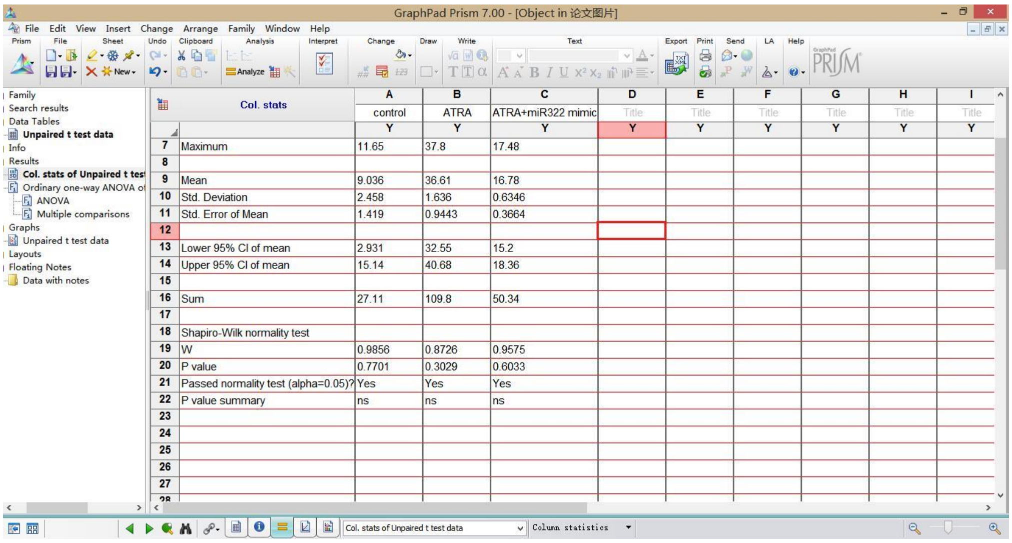

GraphPad Prism 7.00 - [Object in 论文图片]

	Col. stats	A	B	C
		control	ATRA	ATRA+miR322 mimic
		Y	Y	Y
7	Maximum	11.65	37.8	17.48
8				
9	Mean	9.036	36.61	16.78
10	Std. Deviation	2.458	1.636	0.6346
11	Std. Error of Mean	1.419	0.9443	0.3664
12				
13	Lower 95% CI of mean	2.931	32.55	15.2
14	Upper 95% CI of mean	15.14	40.68	18.36
15				
16	Sum	27.11	109.8	50.34
17				
18	Shapiro-Wilk normality test			
19	W	0.9856	0.8726	0.9575
20	P value	0.7701	0.3029	0.6033
21	Passed normality test (alpha=0.05)?	Yes	Yes	Yes
22	P value summary	ns	ns	ns

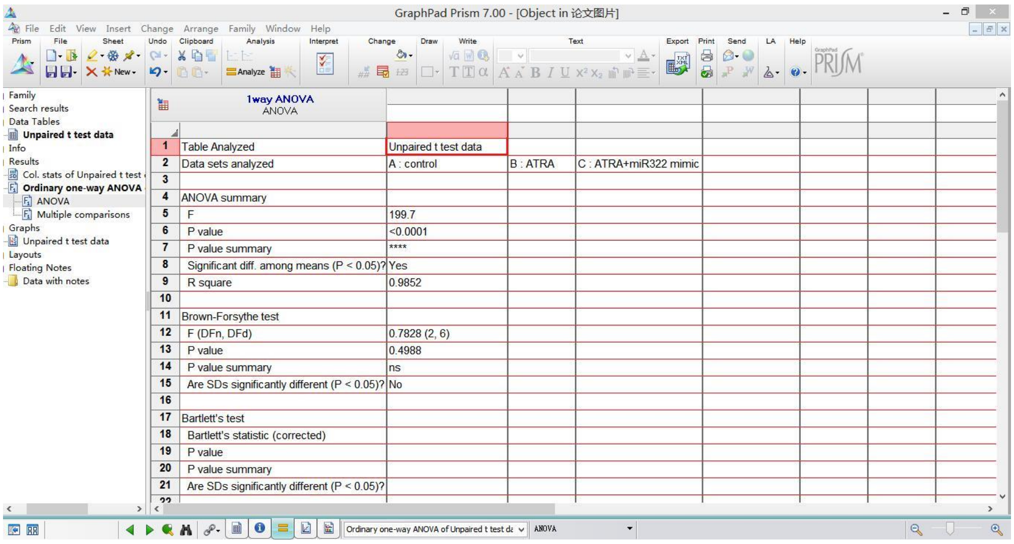

GraphPad Prism 7.00 - [Object in 论文图片]

	1way ANOVA ANOVA			
1	Table Analyzed	Unpaired t test data		
2	Data sets analyzed	A : control	B : ATRA	C : ATRA+miR322 mimic
3				
4	ANOVA summary			
5	F	199.7		
6	P value	<0.0001		
7	P value summary	****		
8	Significant diff. among means (P < 0.05)?	Yes		
9	R square	0.9852		
10				
11	Brown-Forsythe test			
12	F (DFn, DFd)	0.7828 (2, 6)		
13	P value	0.4988		
14	P value summary	ns		
15	Are SDs significantly different (P < 0.05)?	No		
16				
17	Bartlett's test			
18	Bartlett's statistic (corrected)			
19	P value			
20	P value summary			
21	Are SDs significantly different (P < 0.05)?			

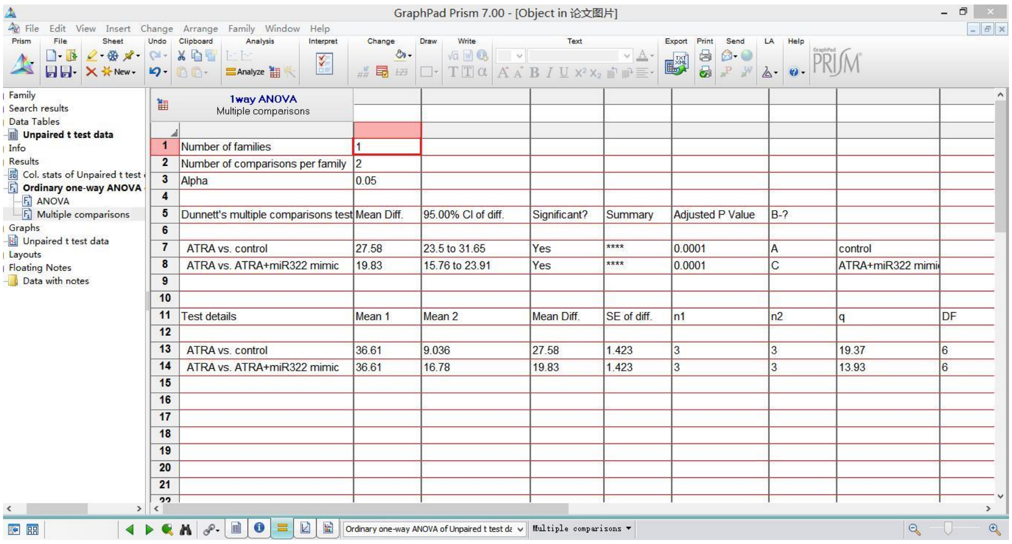
GraphPad Prism 7.00 - [Object in 论文图片]

1way ANOVA
Multiple comparisons

1	Number of families	1							
2	Number of comparisons per family	2							
3	Alpha	0.05							
4									
5	Dunnett's multiple comparisons test	Mean Diff.	95.00% CI of diff.	Significant?	Summary	Adjusted P Value	B-?		
6									
7	ATRA vs. control	27.58	23.5 to 31.65	Yes	****	0.0001	A	control	
8	ATRA vs. ATRA+miR322 mimic	19.83	15.76 to 23.91	Yes	****	0.0001	C	ATRA+miR322 mimi	
9									
10									
11	Test details	Mean 1	Mean 2	Mean Diff.	SE of diff.	n1	n2	q	DF
12									
13	ATRA vs. control	36.61	9.036	27.58	1.423	3	3	19.37	6
14	ATRA vs. ATRA+miR322 mimic	36.61	16.78	19.83	1.423	3	3	13.93	6
15									
16									
17									
18									
19									
20									
21									
